# Large-scale discovery, analysis and design of protein energy landscapes

**DOI:** 10.1038/s41586-026-10465-z

**Published:** 2026-05-13

**Authors:** Állan J. R. Ferrari, Sugyan M. Dixit, Jane Thibeault, Mario Garcia, Scott Houliston, Robert W. Ludwig, Pascal Notin, Claire M. Phoumyvong, Cydney M. Martell, Michelle D. Jung, Kotaro Tsuboyama, Lauren Carter, Cheryl H. Arrowsmith, Miklos Guttman, Gabriel J. Rocklin

**Affiliations:** 1 Department of Pharmacology, Northwestern University Feinberg School of Medicine, Chicago, IL USA; 2 Center for Synthetic Biology, Northwestern University Feinberg School of Medicine, Chicago, IL USA; 3 Structural Genomics Consortium, University of Toronto, Toronto, Ontario Canada; 4 Princess Margaret Cancer Centre, University of Toronto, Toronto, Ontario Canada; 5 Department of Medical Biophysics, University of Toronto, Toronto, Ontario Canada; 6 Department of Systems Biology, Harvard Medical School, Boston, MA USA; 7 Department of Biochemistry, University of Washington, Seattle, WA USA; 8 Department of Medicinal Chemistry, University of Washington, Seattle, WA USA; 9 Robert H. Lurie Comprehensive Cancer Center, Northwestern University Feinberg School of Medicine, Chicago, IL USA; 10 Present Address: Institute of Industrial Science, The University of Tokyo, Tokyo, Japan

**Keywords:** Biophysical methods, Protein design, Computational biophysics

## Abstract

All folded proteins continuously fluctuate between their low-energy native structures and higher-energy conformations that can be partially or fully unfolded. These rare states influence protein function^1,2^, interactions^3^, aggregation^4–7^ and immunogenicity^8,9^, yet they remain far less understood than protein native states. Although native protein structures are now often predictable with impressive accuracy, conformational fluctuations and their energies remain largely invisible^10^ and unpredictable^11–14^, and experimental challenges have prevented large-scale measurements that could improve machine learning and physics-based modelling. Here we introduce a multiplexed experimental approach to analyse the energies of conformational fluctuations for hundreds of protein domains in parallel using intact protein hydrogen–deuterium exchange mass spectrometry. We analysed 5,778 domains 28–64 amino acids in length, revealing hidden variation in conformational fluctuations, even between sequences sharing the same fold and global folding stability. Site-resolved hydrogen exchange nuclear magnetic resonance analysis of 13 domains showed that these fluctuations often involve entire secondary structural elements with lower stability than the overall fold. Computational modelling of our domains identified structural features that correlated with the experimentally observed fluctuations, enabling us to design mutations that stabilized low-stability structural segments. Our dataset enables new machine-learning-based analysis of protein energy landscapes, and our experimental approach promises to profile these landscapes at considerable scale.

## Abstract (web summary)

An analysis of 5,778 domains 28–64 amino acids in length reveals hidden variation in conformational fluctuations, even between sequences sharing the same fold and global folding stability.

## Main

All proteins continuously move between different conformational states, including (typically) a low-energy native folded state, a higher-energy unfolded state and diverse excited states with different levels of native-like structure. Although these higher-energy states are only sparsely populated, they have large impacts across biology and protein engineering, influencing protein function^1,2^, interactions^3^, aggregation^4–7^ and immunogenicity^8,9^. The rare and often transient nature of high-energy states makes them challenging to study experimentally, and these states are often described as invisible to traditional structural biology^10^. Consequently, far less is known about protein excited states compared with protein native states, with no comparable resource to the Protein Data Bank to guide the development of artificial intelligence (AI) and machine learning methods (but see ref. ^15^). AI methods trained to predict native (lowest energy) protein structures have shown little ability to predict protein folding stabilities or the energies of different conformational states without additional data^11–14^.

Understanding high-energy states is also challenging because they are highly sequence specific: every protein has its own conformational energy landscape describing the energies (and therefore populations) of its different conformational states. Energy landscapes can vary considerably between structurally similar proteins, and single mutations can strongly perturb energy landscapes without altering the native protein structure^4,7,16,17^. AI methods for predicting native structures rely on structural conservation across highly diverged sequences, but this conservation does not hold for energy landscapes^18–20^. To develop next-generation AI models that can predict and engineer conformational energy landscapes, we need new experimental methods that can characterize energy landscapes across sequence space and reveal the rules for how protein sequences determine their energy landscapes in a particular environment. Recent advances in measuring global folding stability at scale^21,22^ have accelerated the use of AI methods in biophysics^23–25^. However, these methods do not yet have the ability to resolve the details of conformational fluctuations or identify the range of excited states populated by each protein sequence.

Here we introduce a multiplexed hydrogen–deuterium exchange mass spectrometry (mHDX-MS) strategy to investigate protein energy landscapes for hundreds of protein domains simultaneously. In contrast to experiments that probe only global stability^21,22,26–29^, HDX measures the energies of residue-level transitions between closed conformations (typically in secondary structure) and higher-energy open conformations. Within the same protein, different residues open by accessing different states on the energy landscape, including nearly native states that expose only a few additional residues, alternative folded conformations, partially unfolded states and the globally unfolded state. This enables HDX experiments to measure energies for states that are invisible to other approaches. Traditionally, HDX experiments have been limited to studying one or a few purified proteins at a time^1–4,6–9,17,30^, although recent studies have used simplified cell lysates^31,32^. To enable large-scale HDX analysis of both natural and designed protein domains, we used DNA oligo pool synthesis to produce customized synthetic proteomes comprising up to 1,300 small protein domains per mixture (28–64 amino acids each). Analysing these mixtures using mHDX-MS revealed the exchange rate distributions and approximate opening energy (Δ*G*_open_) distributions for each protein domain.

Overall, we measured the opening energy distributions of 5,778 protein domains from ten families under identical experimental conditions (3,590 after removing low-stability domains). Our dataset revealed wide variation in energy landscapes across sequences with the same overall fold, differences in landscapes between domains sharing the same global folding stability, and systematic differences between domain families. The unique scale of our data (to our knowledge, over 500-fold larger than previous comparative studies of energy landscapes)^4,33–36^ enabled us to use machine learning to identify common determinants of energy landscapes across a broad range of sequences. Our analysis also enabled us to design mutations that enhanced local stability by dampening conformational fluctuations, demonstrating the potential of data-driven approaches to modulate protein energy landscapes.

## The mHDX-MS method

**Fig. 1:**
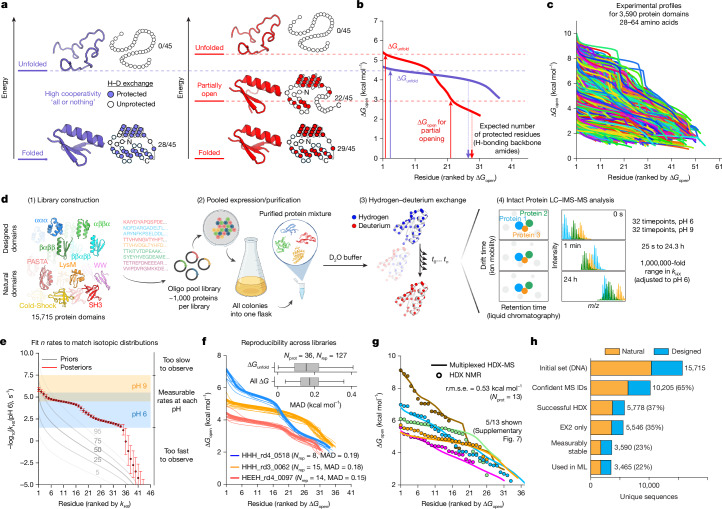
Multiplex HDX-MS to profile energy landscapes. **a**, Idealized energy landscapes for a perfectly two-state protein (left) and a protein with a partially open state (right). Residues are protected from exchange (filled circles) in the folded state and exchange (open) through conformational fluctuations to higher-energy states. **b**, mHDX-MS opening energy profiles for two proteins resembling the idealized landscapes. Opening energies are shown ranked from highest to lowest (mHDX-MS does not resolve which Δ*G*_open_ corresponds to which residue). The two-step Δ*G*_open_ distribution (red) reveals many residues opening through low-energy fluctuations (not global unfolding). **c**, Diverse opening energy profiles identified using mHDX-MS. **d**, Protein domains encoded in a DNA oligo pool are expressed together, purified as a mixture and analysed using LC–IMS-MS across 64 timepoints. Signal identity is defined by constant LC retention and IMS drift times. The diagram was created using BioRender; Phoumyvong, C. https://BioRender.com/8pfr3ys (2026). **e**, Bayesian inference of *n* rates (*k*_HX_) for *n* exchangeable residues (Extended Data Fig. 1). Rates are pH dependent due to *k*_chem_ (ref. ^67^); slower rates are resolved at pH 9 and converted to pH 6 scale. The error bars show the 95% credible intervals. **f**, Reproducibility across *N*_rep_ libraries. Inset: the MAD (Supplementary Fig. 4). For the box plots, the centre lines show the median; the box limits show the 25th–75th percentiles and the whiskers show 1.5 × interquartile range (to data limits). **g**, mHDX-MS results are consistent with HDX NMR (Supplementary Fig. 5). **h**, Experimental outcomes of all sequences. ML, machine learning.

Protein domains have individual energy landscapes that influence how backbone amide hydrogens exchange for deuterium. In an idealized two-state protein with no conformational fluctuations, all protected amides (typically those donating hydrogen bonds in secondary structure, but not always)^37,38^ exchange only from the globally unfolded state (Fig. 1a (left)). This produces a uniform distribution of opening free energies (Δ*G*_open_) because all protected amides exchange from the same state (Fig. 1b (blue)). If the energy landscape includes additional states at intermediate energies that open a subset of amides (Fig. 1a (right)), the distribution of Δ*G*_open_ will be less uniform (Fig. 1b (red)). We developed mHDX-MS to measure these opening energy distributions for hundreds of protein domains in parallel (Fig. 1c). For visualization, we depict Δ*G*_open_ distributions as opening energy profiles ranking measurable residues in a domain from highest/most stable to lowest/least stable (yet measurable) Δ*G*_open_ (Fig. 1b,c).

Our approach begins by constructing customized mixtures of protein domains. Each sample of 108–1,334 domains is encoded as a synthetic DNA oligo pool, cloned into a vector, and expressed and purified as a mixture from a single *Escherichia coli* culture (Fig. 1d). We then incubate the mixture in deuterium oxide (D_2_O) for timepoints from 25 s to 24 h, quench the exchange and analyse each timepoint (32 at pH 6 and 32 at pH 9) using liquid chromatography ion mobility mass spectrometry (LC–IMS-MS; Fig. 1d). Using a customized computational pipeline, we extract full isotopic distributions for each domain at each timepoint. Our pipeline incorporates a tensor factorization approach to resolve overlapping isotopic distributions and an algorithm that identifies the most likely signal for each domain at each timepoint (Supplementary Fig. 1). All analyses are fully automated, with no manual intervention beyond establishing global data quality thresholds.

**Extended Data Fig. 1:**
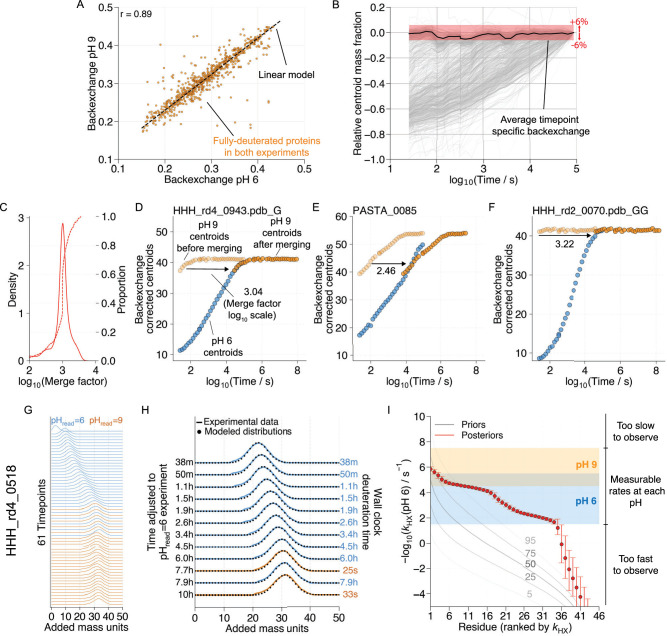
Inferring exchange rates (*k*_HX_) from mHDX-MS data. (**A**) Protein-specific back exchange normalization: we determine the average back exchange for all proteins based on the total deuteration in the last five timepoints, if the deuteration level is no longer increasing. For proteins that do not reach full deuteration at pH 6, we infer their pH 6 level of back exchange based on the back exchange observed at pH 9. We use a linear model to infer pH 6 back exchange based on pH 9 back exchange, parameterized using proteins identified as fully deuterated under both conditions. (**B**) Timepoint-specific back exchange correction**:** injection-to-injection variability leads to a different overall level of back exchange at each timepoint for all proteins. We identify the central trend (black line) using the subset of proteins that reach full deuteration by the fifth-to-last timepoint, with the red shaded area indicating the mass tolerance used to include centroids in computing the trend. This timepoint-specific back exchange is added to the protein-specific back exchange when we infer exchange rates. (**C**) Distribution of merge factors across proteins for which both pH 6 and pH 9 data were combined to infer exchange rates; pH 9 is expected to accelerate observed rates by a factor of 10³^67,81,82^. (**D**–**F**) Representative centroid trajectories highlighting three examples: (**D**) HHH_rd4_0943.pdb_G with a merge factor near 10³. (**E**) PASTA_0085 with a lower factor (2.43), and (F) HHH_rd2_0070.pdb_GG with a higher factor (3.22). (**G**) The merged isotopic distributions (pH 6 in blue, pH 9 in orange) for HHH_rd4_0518, illustrating how timepoints from both conditions form a continuous series when properly merged. (**H**) A closer view of the overlapping timepoint region, where the observed distributions (dots) are compared with theoretical distributions based on the EX2 model (equations (1)–(4)) and inferred rates. (**I**) Ranked exchange rates (*k*_HX_) obtained via Bayesian inference, with the credible interval (5–95%) shown as error bars. The shaded blue and orange areas indicate the dynamic range covered by pH 6 and pH 9 experiments, respectively. The Bayesian priors are shown as grey lines. Error bars indicate 95% credible intervals extracted from the posterior distributions.

We use Bayesian inference to infer each domain’s set of exchange rates (*k*_HX_, *n* rates for *n* exchangeable residues in a domain) based on that domain’s isotopic distributions at each timepoint (equations (1)–(4), Fig. 1e and Extended Data Fig. 1). Although our analysis infers *n* rates for each domain, our measurements on intact domains do not resolve which rates stem from which residues. The Bayesian procedure naturally infers the experimental uncertainty for each rate, and these uncertainties are typically low because many timepoints are measured. Rates that fall outside our measurable timescales show higher uncertainty, as expected (Fig. 1e). We then compute an approximate opening energy (Δ*G*_open_) distribution for each domain based on its *k*_HX_ distribution and its expected exchange rates in the unfolded state (*k*_chem_, equation (2) and Supplementary Fig. 2). These Δ*G*_open_ distributions also reveal the global stability (Δ*G*_unfold_) of each domain from the most stable residues^39,40^; to reduce noise we average the five most stable residues (equation (5) and Supplementary Fig. 3). Note that residual HDX protection in the unfolded state may bias these estimates, although empirically this effect is small (<1 kcal mol^−1^)^41^. Our experiments and analysis produce reproducible *k*_HX_, Δ*G*_open_ and Δ*G*_unfold_ measurements when the same domains are analysed in different libraries, with opening energy profiles typically reproducible within 0.2 kcal mol^−1^ mean absolute deviation (MAD; Fig. 1f and Supplementary Fig. 4).

1 $${k}_{\mathrm{HX},i}=\frac{{k}_{\mathrm{chem},i}\mathrm{(pH)}}{\mathrm{exp}\,\left(\frac{\Delta {G}_{\mathrm{open},i}}{{\rm{R}}T}\right)+1}$$

2 $$\Delta {G}_{{\rm{o}}{\rm{p}}{\rm{e}}{\rm{n}},i}={\rm{R}}T\times {\rm{l}}{\rm{n}}\,\left(\frac{{k}_{{\rm{c}}{\rm{h}}{\rm{e}}{\rm{m}},i}\mathrm{(pH)}}{{k}_{{\rm{H}}{\rm{X}},i}}-1\right)$$

3 $${P}_{{\rm{e}}{\rm{x}}}({\rm{s}}{\rm{e}}{\rm{q}}\,s,{\rm{t}}{\rm{i}}{\rm{m}}{\rm{e}}\,t,{\rm{r}}{\rm{e}}{\rm{s}}{\rm{i}}{\rm{d}}{\rm{u}}{\rm{e}}\,i)=(1-{{\rm{e}}}^{-{k}_{{\rm{H}}{\rm{X}},i}\times t})\times (1-{\rm{B}}{\rm{a}}{\rm{c}}{\rm{k}}{\rm{E}}{\rm{x}}{\rm{c}}{\rm{h}}{\rm{a}}{\rm{n}}{\rm{g}}{\rm{e}}\,(s,t))\times {x}_{{{\rm{D}}}_{2}{\rm{O}}}$$

4 $${{\rm{D}}{\rm{i}}{\rm{s}}{\rm{t}}}_{{\rm{E}}{\rm{X}}2}(s,t,n\,{\rm{t}}{\rm{o}}{\rm{t}}{\rm{a}}{\rm{l}}\,{\rm{r}}{\rm{e}}{\rm{s}}{\rm{i}}{\rm{d}}{\rm{u}}{\rm{e}}{\rm{s}})={\rm{N}}{\rm{a}}{\rm{t}}{\rm{u}}{\rm{r}}{\rm{a}}{\rm{l}}{\rm{A}}{\rm{b}}{\rm{u}}{\rm{n}}{\rm{d}}{\rm{a}}{\rm{n}}{\rm{c}}{\rm{e}}(s)\, \circledast \,{\rm{P}}{\rm{o}}{\rm{i}}{\rm{B}}{\rm{i}}{\rm{n}}({P}_{{\rm{e}}{\rm{x}},s,t,1},\ldots ,{P}_{{\rm{e}}{\rm{x}},s,t,n})$$

5 $$\Delta {G}_{\mathrm{unfold},{{\rm{D}}}_{2}{\rm{O}}}\approx \max (\Delta {G}_{\mathrm{open}})\approx \mathrm{avg}(\Delta {{\rm{G}}}_{\mathrm{open},\mathrm{rank}1},\ldots ,\Delta {G}_{\mathrm{open},\mathrm{rank}5})$$

Residue exchange rates *k*_HX_ for each residue *i* are determined by equilibrium opening free energies Δ*G*_open_ (EX2 regime) and pH-dependent open-state exchange rates *k*_chem_ (equations (1) and (2))^42^. Residue exchange probabilities *P*_ex_ at time *t* depend on *k*_HX_, the back-exchange probability and the mole fraction ${x}_{{{\rm{D}}}_{2}{\rm{O}}}$(equation (3)). Mass distributions over time are modelled as a convolution of natural isotopic abundance and a Poisson binomial (PoiBin) distribution based on all residues’ *P*_ex_ (equation (4)). We measure the mass distributions over time, then infer all *k*_HX_ and all Δ*G*_open_ using an approximate *k*_chem_. Δ*G*_unfold_ is computed from the most stable residues^39^ (note that proline isomerization is not considered) (equation (5)).

**Extended Data Fig. 2:**
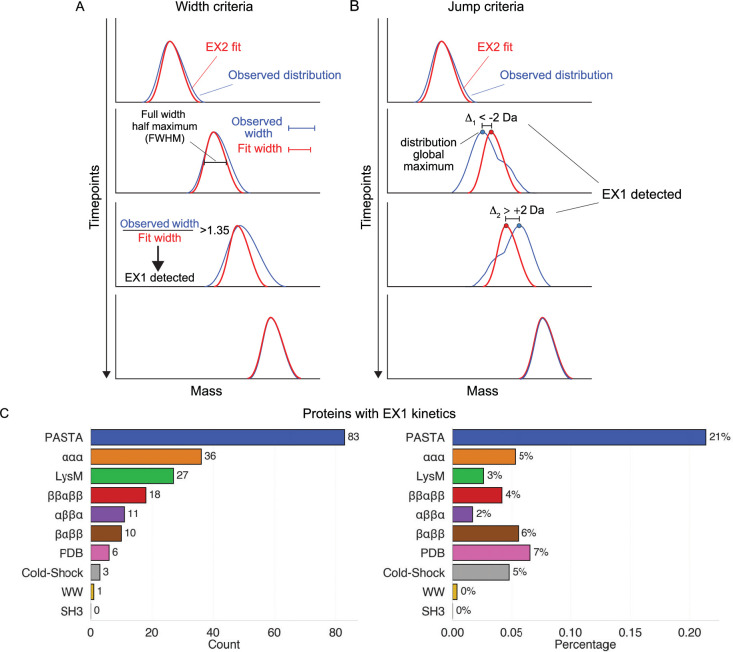
Detection and filtering of EX1 kinetics. (**A**) Illustration of the width criterion: the observed isotopic distribution widths (black) are compared with fitted reference distributions assuming EX2 kinetics (red), and the width ratio is computed during the exchange process (80–90% exchange). Samples with an average ratio >1.25 and a maximum ratio >1.35 are flagged as potential EX1 by the width criterion. (**B**) Illustration of the jump criterion: the difference between the centroid of the fitted EX2 distribution and the maximum peak of the experimental distribution is tracked over time, with abrupt shifts exceeding a predefined range (from <–2 Da to >+2 Da) before 90% exchange indicating EX1 behaviour. (**C**) Summary of EX1 cases identified in our dataset, shown as (left) counts and (right) percentages.

Our inferred *k*_HX_ and Δ*G*_open_ distributions depend on several assumptions. First, we assume all exchanges are independent and follow first-order kinetics (EX2 assumption; equations (3) and (4)), EX1 data are filtered and removed; Extended Data Fig. 2). Second, for many domains, we combine measurements collected at pH 6 and pH 9 into a single model; we assume this pH shift does not alter the energy landscape (measuring exchange at each pH expands the measurable range of Δ*G*_open_ by modulating *k*_*c*hem_; equation (1)). Third, we assume all exchanged residues have equal probabilities to lose deuterium during LC–IMS-MS analysis (back exchange). Finally, our Δ*G*_open_ distributions are only approximate because they depend on estimated *k*_chem_ values (Supplementary Fig. 2). Although these assumptions will not hold for every domain, their overall validity is supported by (1) consistency between our observed and modelled data (all raw data and fits are provided in Supplementary Table 1 (dataset 8)); and (2) consistency between mHDX-MS, HDX NMR and cDNA display proteolysis data (see below), including for domains with unusual energy landscapes and unique dynamics initially identified by mHDX-MS.

## mHDX-MS measurements are accurate

**Extended Data Fig. 3:**
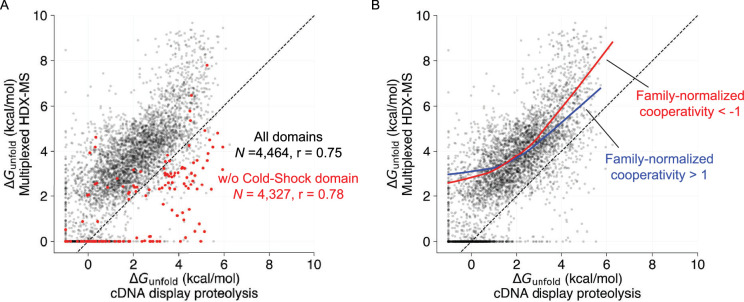
Correlation between Δ*G*_unfold_ measurements by mHDX-MS and cDNA display proteolysis. (**A**) Scatterplots show the Δ*G*_unfold_ values obtained from both methods for 4,464 domains, with an overall Pearson correlation coefficient (r) of 0.75. Cold-Shock domains (highlighted in red), which are known to bind DNA, systematically exhibit greater stability in the cDNA display proteolysis assay. Excluding these domains (*N* = 4,327) improves the correlation to r = 0.78. (**B**) Same plot as in (A), with two LOWESS (locally weighted scatterplot smoothing, Cold-Shock domains excluded) trends highlighted for protein subgroups with family-normalized cooperativity > 1 (blue) and <−1 (red). Proteins with lower cooperativity tend to appear more stable in mHDX-MS than in cDNA display proteolysis within the same cDNA display proteolysis Δ*G*_unfold_, particularly in the high-stability regime. This suggests that cooperative unfolding may impact the relative sensitivity of each method. In both plots, Δ*G*_unfold_ values are clipped to 0 kcal/mol for mHDX-MS and to −1 kcal/mol for cDNA display proteolysis. The black dashed lines indicate y = x, where Δ*G*_unfold_ values would be identical by both methods.

We validated mHDX-MS accuracy using HDX NMR and cDNA display proteolysis. HDX NMR measures gold-standard values for *k*_HX_ and Δ*G*_open_ for individual proteins, whereas cDNA display proteolysis measures Δ*G*_unfold_ for up to 900,000 domains in parallel^22^. Across 13 different domains, mHDX-MS results closely matched HDX NMR measurements, with root mean squared error (r.m.s.e.) of 1.9-fold for *k*_HX_ distributions and 0.53 kcal mol^−1^ for Δ*G*_open_ distributions (Fig. 1g; all 13 domains are shown in Supplementary Fig. 5). These domains span a range of topologies, folding stabilities and Δ*G*_open_ distributions. Discrepancies between mHDX-MS measurements and HDX NMR measurements stem from the assumptions above as well as small experimental differences in temperature, pH, the fraction of D_2_O during exchange and the exact protein constructs. Global stabilities (Δ*G*_unfold_) measured for 4,464 domains by mHDX-MS (in D_2_O) and cDNA display proteolysis (in H_2_O) were also strongly correlated (*r* = 0.78; Extended Data Fig. 3). Stabilities were typically 1.6 kcal mol^−1^ higher in mHDX-MS experiments, probably due to the stabilizing effect of D_2_O^43,44^. mHDX-MS measurements also resolved a wider folding stability range (approximately 2–9 kcal mol^−1^ in mHDX-MS compared to 0–5 kcal mol^−1^ in cDNA display proteolysis; Extended Data Fig. 3). Comparing mHDX-MS and cDNA display proteolysis revealed that nucleic-acid-binding domains have inflated folding stability in cDNA display proteolysis, probably due to stabilization conferred by binding DNA (Extended Data Fig. 3).

## Profiling energy landscapes across families

**Extended Data Fig. 4:**
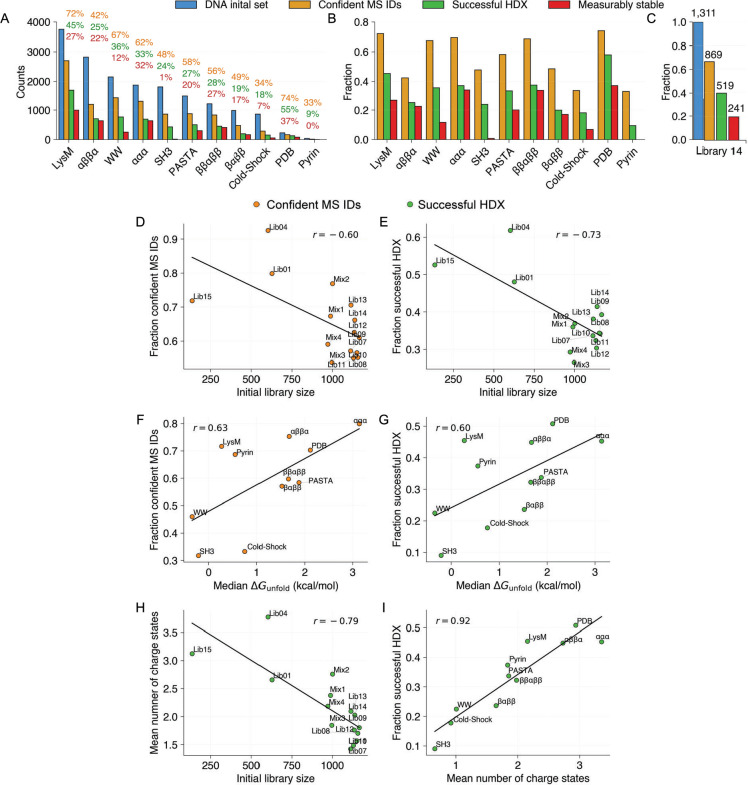
Success rate of mHDX-MS data processing across protein families. (**A**) Raw counts of protein families at successive stages of the analysis pipeline: the set of confident protein identifications (Confident IDs), proteins with successful HDX measurements (Successful HDX), and proteins classified as measurably stable. (**B**) The same three categories are expressed as percentages relative to the initial DNA set, illustrating the attrition at each processing stage. (**C**) A detailed breakdown for Library 14, displaying the counts for confident IDs, successful HDX, and measurably stable proteins obtained in one single experiment. (**D**–**E**) Relationship between technical scale and success rate, showing the fraction of (**D**) confident MS IDs and (**E**) successful HDX measurements as a function of the initial library size. (**F**–**G**) Relationship between biophysical properties and success rate, showing the fraction of (**F**) confident MS IDs and (**G**) successful HDX measurements as a function of the median global stability Δ*G*_unfold_ per protein family, as evaluated by independent cDNA display proteolysis. (**H**) Impact of spectral complexity on data quality, showing the mean number of charge states per library as a function of library size; larger libraries exhibit decreased spectral quality due to increased m/z redundancy and ion suppression. (**I**) Impact of spectral quality on HDX success, showing the fraction of successful HDX per family as a function of the mean number of charge states. For panels H and I, non-identified proteins are assigned a number of charge states value of zero. Linear regression lines are shown in black; r indicates the Pearson correlation coefficient.

**Extended Data Fig. 5:**
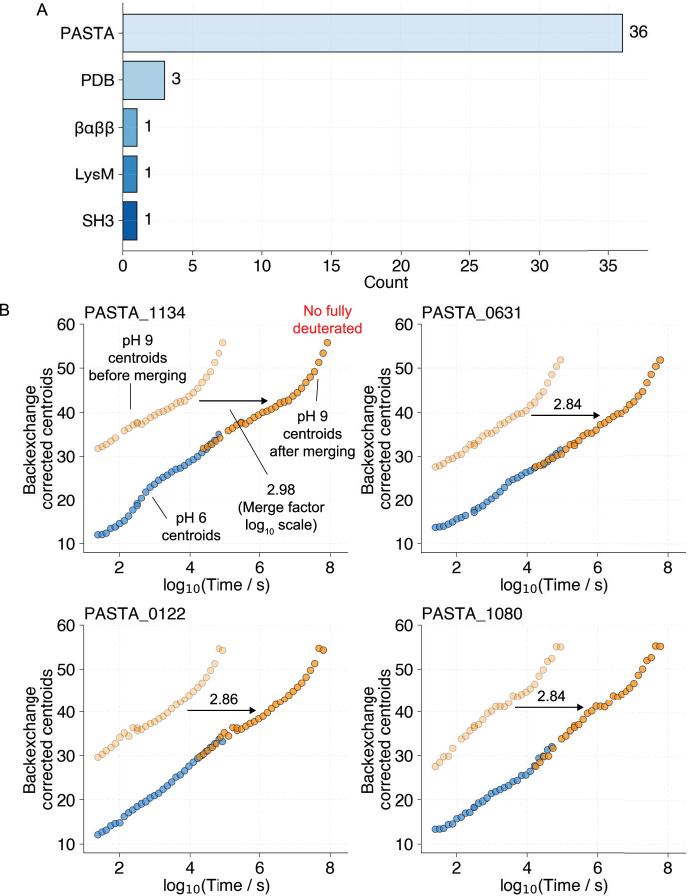
Incomplete deuteration in protein domains. (**A**) Number of proteins exhibiting a difference greater than 2 Da between the centroid masses measured at the last and fifth-to-last timepoints during the pH 9 experiment, indicating incomplete deuteration. (**B**) Four PASTA domains with the highest mass differences between the last and fifth-to-last timepoints, suggestive of very slow exchange rates. Blue (pH 6) and transparent orange (pH 9) points indicating the centroid of the mass envelope (after correcting for back exchange and the mole fraction of D_2_O) are shown at the true experimental timepoint. Solid orange points (pH 9) are shown at the inferred time point on a pH 6 scale after merging the pH 6 and pH 9 data together, with the merge factor indicating the adjustment made to the pH 9 timepoints (on a log_10_ scale). A merge factor of 3 (i.e. a 1,000-fold difference in *k*_HX_) is expected between pH 6 and pH 9 in the ideal case where pH only modulates *k*_chem_ according to^67,81,82^.

From an initial pool of 15,715 sequences, we successfully analysed 5,778 domains by mHDX-MS. These domains came from four families of de novo designed sequences (ααα, βαββ, αββα and ββαββ)^11,21^, six natural domain families from the Pfam database^45^ (LysM, PASTA, WW, SH3, pyrin and cold-shock^45^ and additional small domains from the Protein Data Bank (PDB). Within each family, pairwise sequence identities averaged 35–47% (Supplementary Fig. 6). Domains were assayed in 18 separate libraries containing 108–1,334 sequences. Different sequences failed at different stages owing to differences in protein expression, signal intensity or HDX data quality (Fig. 1h and Extended Data Fig. 4). Success also varied by protein family (Extended Data Fig. 4). Among natural domains, 54% had Δ*G*_unfold_ < 2 kcal mol^−1^, compared with only 10% of designed domains, which were preselected for known experimental stability^11,21^. The largest number of domains successfully analysed in one library was 519, from a library with 1,311 initial sequences (Extended Data Fig. 4c). Nearly all domains became fully deuterated after 24 h at pH 9 except for 42 extremely stable domains (mainly PASTA domains; Extended Data Fig. 5).

## Quantifying opening cooperativity

**Fig. 2:**
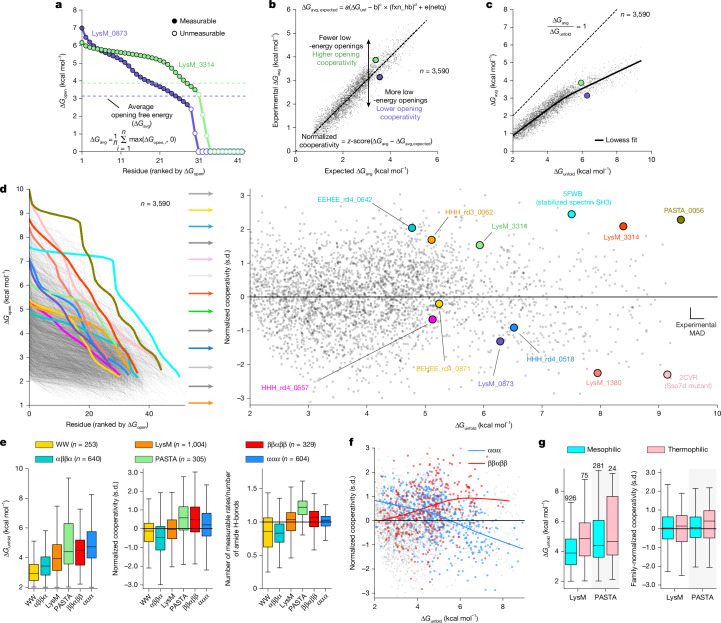
Small domains vary in stability and opening cooperativity. **a**, We calculate the average opening energy (Δ*G*_avg_) over all exchangeable residues as a proxy measure for the energies of partially open states. Low-stability residues with unmeasurably fast exchange rates are included in the average and set at 0 kcal mol^−1^. **b**, Five-parameter empirical model to predict Δ*G*_avg_ from (1) Δ*G*_unfold_; (2) the fraction of backbone amide residues donating hydrogen bonds (fxn_hb); and (3) the protein net charge (netq). We define normalized cooperativity as the standardized difference (*z* score) between each protein’s observed Δ*G*_avg_ and the model’s expected Δ*G*_avg_. The green and blue circles show the protein domains from **a**. **c**, Experimental Δ*G*_avg_ increases sublinearly with Δ*G*_unfold_, highlighting the proteins in **a**. **d**, Opening energy profiles for all domains shown in full (left) or simplified to two dimensions: Δ*G*_unfold_ (*x* axis) and normalized cooperativity (*y* axis). Twelve proteins are highlighted and shown in the same colour in each plot. The scale bars show experimental MADs between replicates measured in different libraries (0.2 kcal mol^−1^ for stability and 0.3 s.d. for normalized cooperativity). **e**, Distributions of Δ*G*_unfold_ (left), normalized cooperativity (middle), and the ratio of the number of measurable rates to the number of backbone amide H-bonds (right) for stable domains in the six families with the most experimental data. **f**, Normalized cooperativity trends with Δ*G*_unfold_ for two protein families. The coloured lines show Lowess fits. **g**, Distributions of Δ*G*_unfold_ (left) and family-normalized cooperativity (right) for domains from mesophilic and thermophilic organisms. For **e**–**g**, the box plots show the median (centre lines), 25th–75th percentiles (box limits) and 1.5× the interquartile range (to data limits) (whiskers).

Our mHDX-MS experiments revealed diverse Δ*G*_open_ distributions across our set of 3,590 stable domains. In contrast to large-scale measurements of fitness^46–48^ or global stability^21,22,26–29^, Δ*G*_open_ distributions are inherently multidimensional. One axis of variation is global stability^39,40^ (Δ*G*_unfold_ measured based on the most stable residues; equation (5)), which varied from below 2 to around 9 kcal mol^−1^ (in D_2_O). However, most residues exchange through conformational fluctuations that are lower in energy than Δ*G*_unfold_ (Figs. 1c and 2a). To quantify this and reduce the dimensionality of our data, we computed the average Δ*G*_open_ over all exchangeable residues for each domain (Δ*G*_avg_, unmeasurably fast residues were set to a lower bound of 0 kcal mol^−1^; Fig. 2a). Domains with similar Δ*G*_unfold_ and native structures often differed substantially in Δ*G*_avg_ (Fig. 2a). In some cases, this reflects differences in hydrogen bonding, because residues lacking amide H-bonds typically have low Δ*G*_open_ regardless of conformational stability (these residues are open in the native state)^37,38^. The remaining variation in Δ*G*_avg_ (after hydrogen bonding has been accounted for) reveals hidden differences in conformational fluctuations between different domains—even between domains with similar native structures and similar global stability.

To isolate differences in fluctuations between domains, we built a five-parameter empirical model of Δ*G*_avg_ based on Δ*G*_unfold_, hydrogen bonding and protein net charge (included for technical reasons; Methods). This model explains 89% of Δ*G*_avg_ variance (Fig. 2b). The remaining variance in Δ*G*_avg_ (the model residuals) results from differences in conformational fluctuations. Domains with a positive residual have higher conformational stability (higher Δ*G*_open_) across many residues compared to the typical domain in our dataset with similar global stability (Fig. 2a,b (green)), whereas domains with a negative residual (Fig. 2a,b (blue)) have more low-energy fluctuations (low Δ*G*_open_) compared with what our model expects given Δ*G*_unfold_ and hydrogen bonding. We define each domain’s normalized residual (*z* score; Methods) as its normalized cooperativity; a metric reflecting the level of conformational fluctuations relative to domains with similar global stability. In protein folding, proteins are considered to be cooperative when they fold in a two-state, all-or-nothing manner. Hydrogen-exchange studies have complicated our understanding of cooperativity by revealing numerous partially folded states across many proteins that previously appeared to be two-state^49–53^. Our normalized cooperativity metric describes a continuum between domains where partially open states are relatively rare and high in energy, approximate ideal two-state behaviour (Fig. 2a (green)) and domains with widespread low-energy partial openings (Fig. 2a (blue)). We refer to this continuum as opening cooperativity, reflecting the degree of all-or-nothing character in the opening energy landscape.

**Extended Data Fig. 6:**
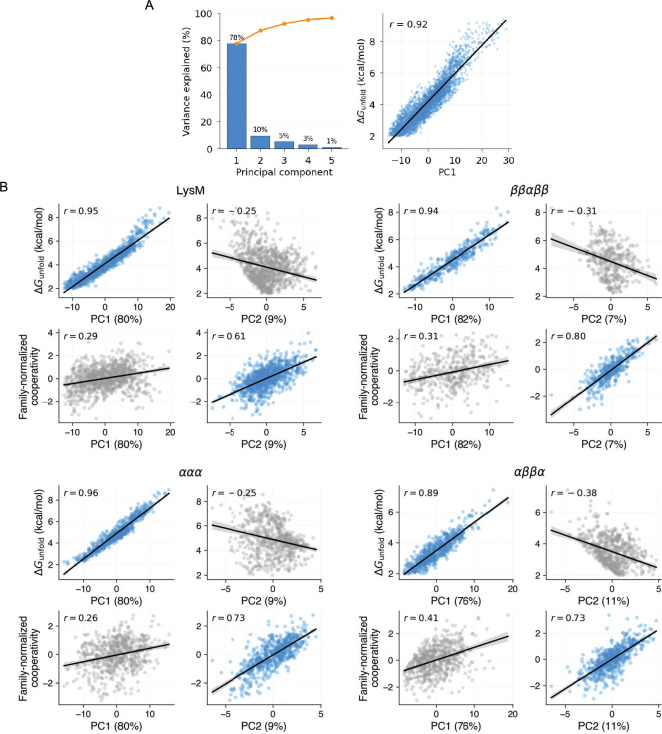
Principal component analysis of opening energy profiles. Principal component analysis (PCA) was applied to opening free-energy profiles derived from mHDX-MS. When PCA is performed across all proteins, the first principal component (PC1) dominates the variance and is strongly correlated with the global folding free energy, Δ*G*_unfold_ (r = 0.92; panel **A**). In contrast, when PCA is performed separately within the four most populated protein families, PC1 remains correlated with Δ*G*_unfold_ (r = 0.89-0.96, panel **B**), while the second principal component (PC2) is strongly correlated with family-normalized cooperativity (r = 0.61-0.80; panel **B**). These results indicate that normalized cooperativity captures the dominant variation in opening energy profiles after removing the contribution of global stability.

We next examined how opening cooperativity relates to folding stability. Across 3,590 stable domains, Δ*G*_avg_ increases sublinearly with Δ*G*_unfold_ (Fig. 2c and Supplementary Fig. 7), indicating higher stability proteins generally have significant openings occurring at much lower energies. This has been described previously: highly stable proteins often appear less cooperative in hydrogen-exchange experiments^51^ because they have a wider range of energies below Δ*G*_unfold_ where partially open states can be found^52^. Our empirical model (Fig. 2b) captures this dependence, making normalized cooperativity nearly orthogonal to Δ*G*_unfold_. By defining normalized cooperativity relative to domains with similar stability using our empirical model, we decouple normalized cooperativity from stability (Fig. 2d). Principle component analysis (PCA) of opening energy profiles yields similar results: the first principal component correlates with Δ*G*_unfold_ (*r* = 0.89–0.96), and the second is strongly correlated with normalized cooperativity (*r* = 0.61–0.80; Extended Data Fig. 6). However, in contrast to PCA, normalized cooperativity isolates the differences in conformational fluctuations between proteins by explicitly removing variation in opening energy profiles caused by differences in protein length, hydrogen bonding and net charge.

We observed systematic differences in stability and cooperativity between families (Fig. 2e,f), although intrafamily variation in cooperativity was larger than interfamily differences. This indicates that the individual sequences strongly influence cooperativity beyond the average tendency of the overall fold. Of the six families with the most data, PASTA domains (an α/β fold) and de novo designed ββαββ domains showed the highest average cooperativity (Fig. 2e), possibly owing to their β-sheet architecture. The highly cooperative PASTA domain family also showed a relatively high number of protected residues compared with the number of predicted H-bonding amides from reference structures (Fig. 2e; cases of unexpected protection have been found previously^37,38^). Although normalized cooperativity is (intentionally) nearly orthogonal to stability across all 3,590 stable domains (Fig. 2d), certain domain families showed clear relationships between stability and cooperativity within the family (Fig. 2f). To better analyse intrafamily trends, we defined an additional family-normalized cooperativity metric by fitting the empirical model from Fig. 2b separately for each different family (Supplementary Fig. 7 and Supplementary Table 2). Finally, comparing mesophiles and thermophiles within the LysM and PASTA families (the only families with enough data from thermophiles^54^; Fig. 2g) revealed that LysM domains from thermophiles have significantly higher global stability on average (mean difference of 0.8 ± 0.4 kcal mol^−1^, mean ± 95% confidence intervals (CI) from bootstrapping). PASTA domains showed a similar but statistically insignificant difference (0.4 ± 0.9 kcal mol^−1^). We did not observe significant differences in normalized cooperativity (mean differences of 0.0 ± 0.2 s.d. and 0.2 ± 0.5 s.d. for the LysM and PASTA domains, respectively).

## The spatial distribution of stability

**Fig. 3:**
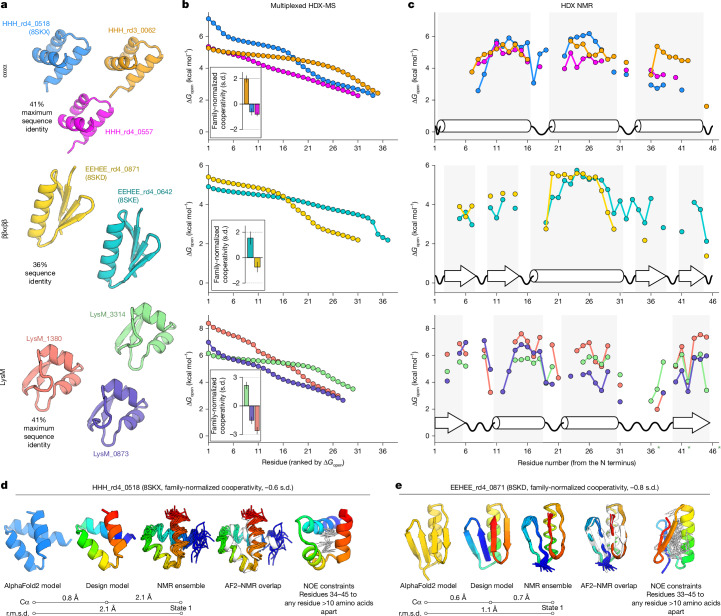
Low-cooperativity domains show clustering of unstable residues. **a**, AlphaFold models of exemplar domains. **b**, mHDX-MS opening energy profiles for exemplar domains. The insets show family-normalized cooperativity; the confidence intervals show MADs from 8–15 replicates in different libraries for ααα and ββαββ domains and average experimental MAD across 36 proteins for LysM domains. **c**, Opening energies for exemplar domains from HDX NMR collected under multiple pH and temperature conditions as needed (Extended Data Fig. 7, Supplementary Figs. 5 and 8 and Supplementary Table 1 (dataset 4)). Secondary structures are shown at bottom and shaded; some residues in the secondary structure (helical first turns and edge-pointing strand residues) do not donate H-bonds and are not expected to be protected. For LysM_3314, residues 35–45 are plotted at sites 36–46 (green asterisks) based on structural alignment to the other LysM domains. **d**, Solution NMR structure of the low-cooperativity de novo protein HHH_rd4_0518 compared with the AlphaFold 2 and computational design models; in the NMR-derived structure, α3 adopts the correct fold and tertiary contacts despite its low stability. NOE, nuclear Overhauser effect; r.m.s.d., root mean squared deviation. **e**, Solution NMR structure as in **d**, for the low-cooperativity de novo protein EEHEE_rd4_0871. The C-terminal hairpin is correctly folded in the NMR ensemble, where it contacts the helix and N-terminal hairpin.

**Extended Data Fig. 7:**
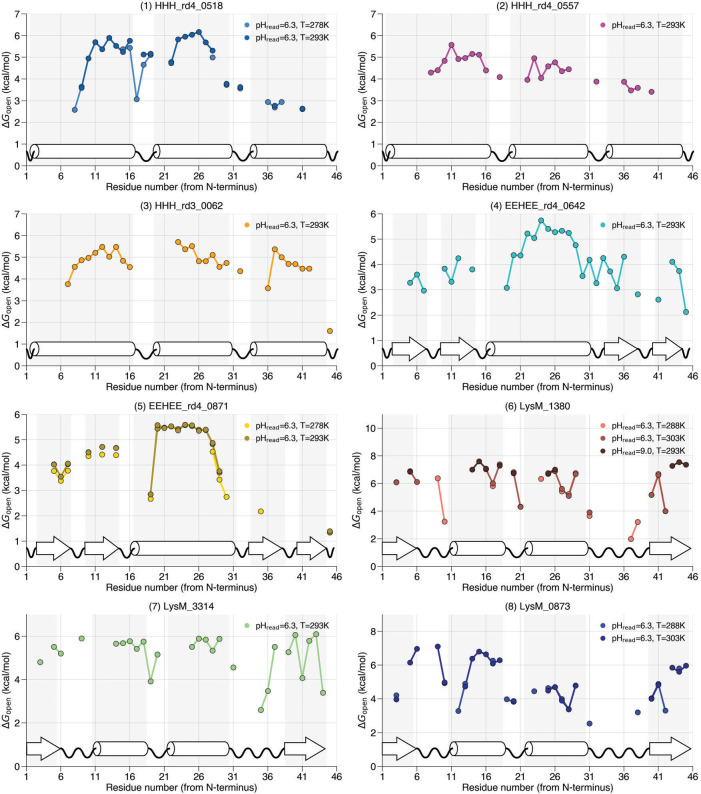
Residue-level HDX NMR profiles for the eight proteins shown in Fig. 3, measured under multiple experimental conditions. For each protein, coloured scatters and lines represent data acquired at distinct pH and temperature conditions, with each shade corresponding to a specific experimental setup.

Highly cooperative domains are all alike; every less-cooperative domain fluctuates in its own way. Low-stability residues might be dispersed throughout the structure (for example, secondary structures fraying at their ends) or clustered in specific unstable elements of the structure. To investigate the spatial distribution stability, we used site-resolved HDX NMR to analyse five low-cooperativity proteins and three high-cooperativity contrasting examples (Fig. 3a,b, Extended Data Fig. 7 and Supplementary Fig. 8). In four out of five low-cooperativity domains, unstable residues were clustered in specific structural regions (Fig. 3c). For example, the de novo designed protein HHH_rd4_0557 (magenta, family-normalized cooperativity −0.8 s.d.) showed a tiered stability landscape for its three helices (α1 > α2 > α3). The design HHH_rd4_0518 (blue, −0.6 s.d.) showed larger differences in stability between helices: the cores of α1 and α2 open near 6 kcal mol^−1^, whereas α3 opens below 3 kcal mol^−1^ (Fig. 3c). We solved the solution structure of HHH_rd4_0518 by NMR, which closely matched the designed structure and AlphaFold model, with the unstable α3 helix correctly folded in the native state (Fig. 3d and Extended Data Table 1). Thus, the faster exchange from α3 occurs through a (relatively low energy) excited state, not the native state. As expected, the highly cooperative HHH_rd3_0062 (orange, +2.0 s.d.) showed uniform opening energies across all helices (Fig. 3c).

The low-cooperativity design EEHEE_rd4_0871 (Fig. 3 (yellow), −0.8 s.d.) and the low-cooperativity natural domain LysM_0873 (purple, −1.5 s.d.) also showed clustering of low-stability residues. In EEHEE_rd4_0871, the C-terminal β-hairpin is much less stable than the rest of the structure and is essentially unmeasurable by NMR (Fig. 3c). We solved the structure of both EEHEE_rd4_0871 and the high-cooperativity example EEHEE_rd4_0642, which both matched the designed models (Fig. 3e and Extended Data Table 1). As with HHH_rd4_0518, the unstable C-terminal β-hairpin of EEHEE_rd4_0871 folds as designed despite its low-energy fluctuations. In LysM_0873, unstable residues cluster in α2 and β2 (Fig. 3c). The highly cooperative domains EEHEE_rd4_0642 (teal, +1.5 s.d.) and LysM_3314 (green, +2.1 s.d.) showed more uniform stabilities across secondary structures.

The fifth low-cooperativity domain LysM_1380 (Fig. 3 (red); −2.6 s.d.) did not show significant clustering of unstable residues. Instead, the variation within each secondary structure was greater than in the other LysM domains. Many residues in LysM_1380 (such as positions 6, 9, 10, 17, 27, 28 and 38) showed lower stability than homologous positions in the other two LysM domains despite the higher global stability of LysM_1380 (Fig. 3c).

Overall, these results show that low opening cooperativity typically results from specific unstable structural elements, although not always. Bimodal Δ*G*_open_ distributions like those seen for HHH_rd4_0518, EEHEE_rd4_0871 and LysM_0873 may be evidence of spatial clustering of stability, but this is not required. Importantly, residues sharing the same Δ*G*_open_ may open together or through different isoenergetic partially open states. Finally, our results illustrate that highly stable small domains can still have relatively unstable structural elements: HHH_rd4_0518, EEHEE_rd4_0871 and LysM_0873 are in the 76–94th percentiles for global stability in their families despite low energy openings in specific structural elements.

## Structural determinants of cooperativity

**Fig. 4:**
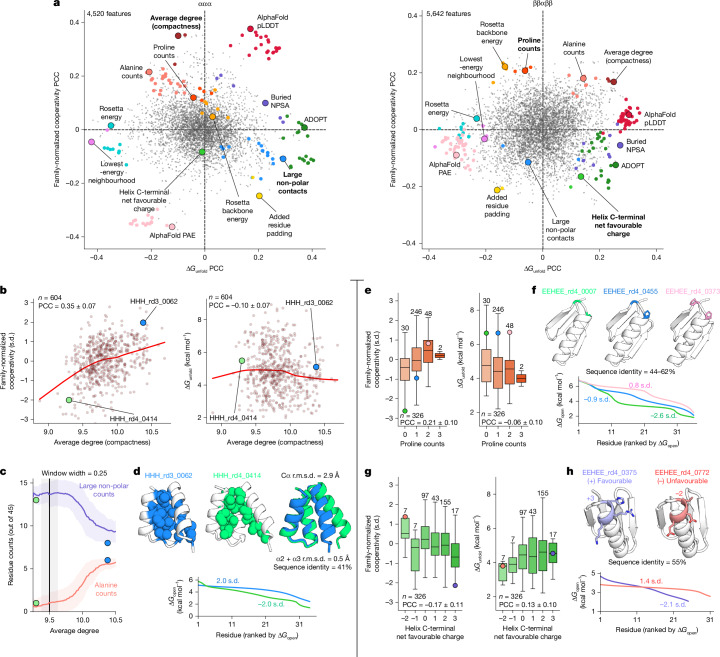
Protein features correlating with cooperativity. **a**, The PCC between protein features and family-normalized cooperativity (*y* axis) or Δ*G*_unfold_ (*x* axis) for ααα (left) and ββαββ (right) protein families. The large coloured circles highlight notable features; the small coloured circles show features that are closely related to the same-colour-labelled feature (interfeature PCC > 0.75); features in bold are highlighted below. **b**, Average degree compactness (average Cα count within 9.5 Å of each Cα) positively correlates with family-normalized cooperativity in the ααα family but has a small negative correlation with Δ*G*_unfold_. The large circles highlight two proteins shown in **d**. PCC 95% CIs were bootstrapped. **c**, Moving average (±s.d., shading) of alanine counts (pink) and large non-polar (FILMVWY) counts (purple) across average degree. The large circles show the two domains in **d**. **d**, Examples of high-compactness (blue) and low-compactness (green) domains. Top, AlphaFold models with hydrophobic core residues highlighted. Bottom, opening energy profiles and family-normalized cooperativity. Differences are illustrative; multiple factors influence cooperativity. **e**, Proline counts positively correlate with family-normalized cooperativity in the ββαββ family but negatively correlate with Δ*G*_unfold_. The circles highlight proteins in **f**. The numbers indicate examples per bin. **f**, Example ββαββ domains with zero (green), one (blue) and two (pink) proline residues. Top, AlphaFold models. Bottom, opening energy profiles and family-normalized cooperativity. **g**, Helix C-terminal net favourable charge (His+Lys+Arg count minus Asp+Glu count in the last three helical residues) negatively correlates with family-normalized cooperativity in the ββαββ family but positively correlates with Δ*G*_unfold_. The circles highlight two example proteins shown in **h**. **h**, Example ββαββ domains with three favourable (+) charges (blue) and two unfavourable (−) charges (red). Top, AlphaFold models. Bottom, opening energy profiles and family-normalized cooperativity. For the box plots in **e**–**g**, the centre lines show the median, the box limits show the 25th–75th percentiles and the whiskers show 1.5× the interquartile range (to data limits).

**Extended Data Fig. 8:**
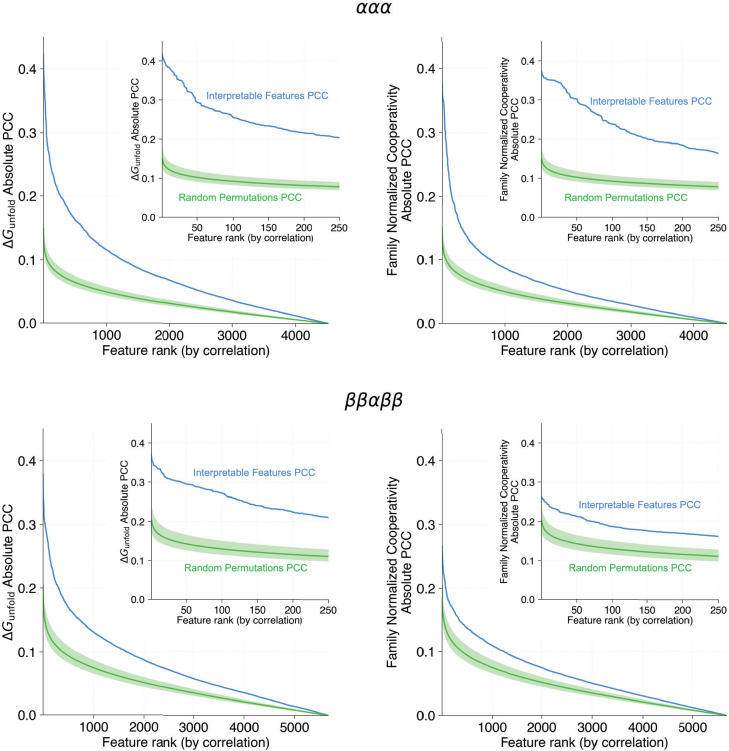
Permutation experiment for feature importance. In this analysis, 10,000 random permutations of the target variables - global stability (Δ*G*_unfold_) on the left and family-normalized cooperativity on the right - were generated for the ααα (top panels) and ββαββ (bottom panels) topologies. For each permutation, the Pearson correlation coefficients (PCCs) were computed between each manually engineered interpretable feature and the randomized target, and the features were then ranked according to these PCCs. This procedure essentially derives the order statistics of the random correlations, with the highest correlation value (first order statistic), the second highest, and so on, recorded to form a null distribution for each rank. Blue curves indicate the PCCs computed from the actual interpretable features, while green curves show the mean and 95% confidence intervals (shaded) of the corresponding null distributions. These comparisons demonstrate that the manually engineered features yield significantly higher correlations than expected by chance.

To identify biophysical determinants of opening cooperativity, we modelled the structures to compute thousands of sequence- and structure-based features for each domain. We then analysed Pearson correlation coefficients (PCCs) between each feature and our experimental measurements of global stability and family-normalized cooperativity (Fig. 4a). Features included primary sequence properties (such as amino acid composition), Rosetta energetic terms^55^ and machine-learning-derived metrics from tools such as AlphaFold^56^, secondary structure predictors and disorder predictors. To account for the large (and partially redundant) feature set, we compared results with permuted data; many correlations significantly exceeded the 95th percentile expected by chance (Extended Data Fig. 8). We focused on analysing the ααα and ββαββ families, where correlations were strongest (Fig. 4a and Supplementary Fig. 9). No single feature dominated the correlations (maximum absolute PCCs with cooperativity 0.38 ± 0.07 for ααα domains and 0.27 ± 0.09 for ββαββ domains, mean ± 95% CI from bootstrapping), suggesting that cooperativity is influenced by multiple factors, or that important determinants are missing from our set.

We identified protein features that correlated with cooperativity alone, stability alone or both (Fig. 4a). In both families, the Rosetta model’s total energy score^55^, buried non-polar surface area and the ADOPT disorder predictor score^57^ correlate relatively strongly with global stability, but weakly with cooperativity (Fig. 4a). By contrast, proline counts show minimal correlation with folding stability but shows one of the strongest correlations with cooperativity in the ββαββ family (with a similar but weaker trend in ααα). AlphaFold2’s pLDDT confidence metric positively correlated with both stability and cooperativity in both families. Overall, most features had directionally similar (but different strength) correlations between the two families.

Many features had opposite relationships with cooperativity and global stability. In the ααα family, the average degree compactness metric (average Cα count within 9.5 Å of each Cα) had one of the strongest positive correlations with cooperativity, but a negative correlation with global stability (Fig. 4b). Alanine count exhibited a similar pattern (Fig. 4a). Together these suggest an underlying trade-off: increased compactness promotes opening cooperativity, but this compactness is often achieved through greater alanine content and fewer large non-polar amino acids (Fig. 4c,d), which is modestly destabilizing.

In the ββαββ family, proline counts had one of the strongest positive correlations with cooperativity but a negative correlation with global stability (Fig. 4e). Prolines were consistently located in the same two positions in the loop connecting the second strand to the helix (Fig. 4f). Whereas positive charges in the final turn stabilize helices by counteracting the backbone dipole^58^—a trend observed in our 326 ββαββ domains (PCC, 0.13 ± 0.10; Fig. 4g)—this feature had a stronger negative correlation with cooperativity (PCC, −0.17 ± 0.11; Fig. 4g; examples are shown in Fig. 4h). This suggests that these charges primarily stabilized the helix with minimal cooperative increase in the stability of the sheet. If the helix has residual stability in the unfolded state^59^, this would also lower our inferred cooperativity by increasing the stability difference between the helix and the remaining residues.

While these correlations provide insights, they depend on our dataset and may not represent all folded domains. Correlations between different features (including correlations introduced by Berkson’s paradox and the minimal requirement of folding) also make it difficult to infer causal relationships. Still, the scale of our dataset enabled the discovery and quantification of correlates of opening cooperativity across a uniquely broad range of sequences. The consistency of many trends between the ααα and ββαββ families also suggests that these trends may generalize further.

## Predicting stability and cooperativity

Individual features were only modestly correlated with global stability (Δ*G*_unfold_) and family-normalized cooperativity, indicating that these properties are governed by multiple factors. To examine this, we trained machine learning models to predict Δ*G*_unfold_ and family-normalized cooperativity for the four most data-rich families (ββαββ, αββα, ααα and LysM). We used the engineered features from Fig. 4, feature expansion and selection strategies, and protein language models (PLMs) embeddings to train Lasso and Ridge regression models, which we evaluated using fivefold cross-validation. Models were family specific (trained and tested on a single fold family), but domains within families were clustered by sequence identity and clusters were assigned to the same fold (30–55% maximum identity between folds).

**Extended Data Fig. 9:**
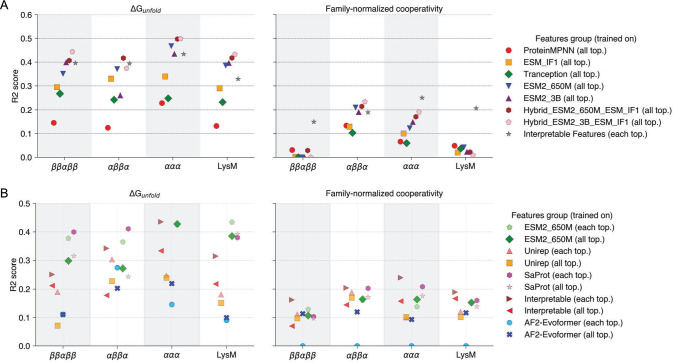
Performance of machine learning models. (**A**) First-generation models: R² scores for predictions of Δ*G*_unfold_ (left) and family-normalized cooperativity (right) are shown for models trained using protein language model embeddings derived from ProteinMPNN, ESM_IF1, Tranception, and ESM2 (650 M and 3B parameters), as well as hybrid combinations, and also using interpretable features. In this initial approach, sequences were randomly assigned to 5-folds without identity-based clustering. (**B**) Second-generation models: R² scores for the same targets are presented for models incorporating dynamic clustering via MMseqs2 to group similar sequences, ensuring that each fold is as distinct as possible while maintaining balanced representation across protein families. In both cases, models were trained using 5-fold cross-validation over three independent random splits, and performance was evaluated in the aggregated predictions within each family-specific predictions. Models were trained either using examples from all four topologies (labelled as ‘all top.’) or specifically with examples from the targeted topology (labelled as ‘each top.’). For visualization purposes, R² values below zero were clipped to zero.

Prediction accuracy for family-normalized cooperativity was relatively low, with the best coefficients of determination (*R*^2^) ranging from 0.16 to 0.24 on unseen data (Extended Data Fig. 9b). Still, these multifeature models had higher correlations (even on unseen data) than the strongest single features examined in Fig. 4a (highest single-feature *R*^2^ of 0.05–0.14 without cross-validation). By contrast, models trained to predict Δ*G*_unfold_ performed better (*R*^2^, 0.40–0.53), highlighting that fine differences in opening energy profiles are harder to predict than global stability. While PLM embeddings yielded the most accurate stability predictions, manually engineered interpretable features based on explicitly modelled structures produced the highest accuracy for cooperativity across all families (Extended Data Fig. 9b).

## Designed mutations increase cooperativity

**Fig. 5:**
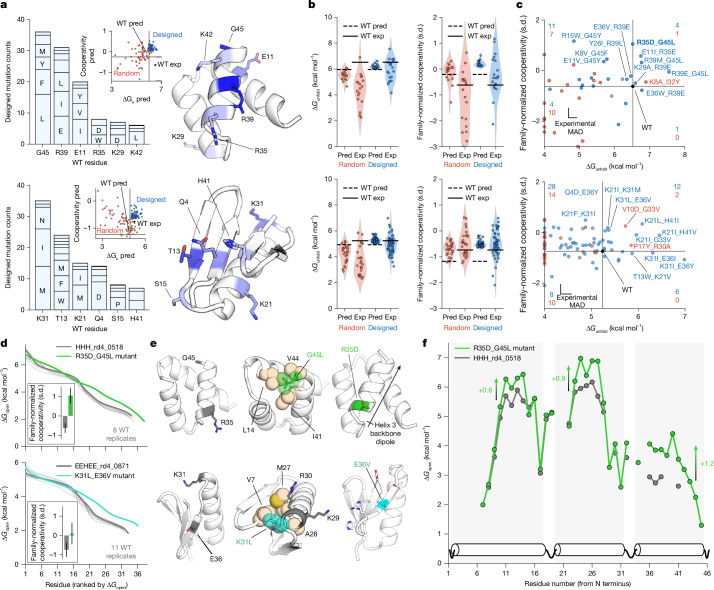
Data-driven design of improved cooperativity. **a**, The mutation frequency in designed double-mutant libraries of HHH_rd4_0518 (top) and EEHEE_rd4_0871 (bottom) at the top six mutated sites (left). Structures (coloured by mutation frequency) are AlphaFold models. Insets: predicted Δ*G*_unfold_ (*x* axis) and family-normalized cooperativity (*y* axis) for designed (blue) and randomly chosen (red) double mutants from linear machine learning models. The lines show the predicted values for the (held-out) wild-type sequence (WT pred) from the same models; experimental wild-type (WT exp) values are marked by a black diamond. **b**, Comparison of predicted (pred) and experimental (exp) stability (left) and cooperativity (right) measurements for designed (blue) and random (red) double mutants of HHH_rd4_0518 (top) and EEHEE_rd4_0871 (bottom). Each wild-type (WT) sequence is shown as a black line. **c**, The experimental results of stable designed (blue) and random (red) double mutants of HHH_rd4_0518 (top) and EEHEE_rd4_0871 (bottom). WT sequences are shown in black, and single mutants are shown in grey. Sequences outside the plot domain are shown on the edges. Quadrants show designed and random mutants counts. Experimental MADs are shown and computed from replicates of other sequences (Supplementary Fig. 4). Results organized by mutation are shown in Supplementary Fig. 11. **d**, The opening energy profiles of double mutants with improved family-normalized cooperativity (colours). Replicates of WT measurements from different libraries are shown in grey with the highest quality measurement as a thick line. Inset: family-normalized cooperativity for WT and mutant sequences. The confidence intervals indicate MADs from replicates of the WT in different libraries or the MAD across our whole dataset for the mutants. **e**, The structural effects of the mutations from **d** shown on AlphaFold structures (see the main text). **f**, HDX NMR analysis of HHH_rd4_0518 (grey) and the G45L/R35D double mutant (green). Stability differences between the WT and mutant were calculated by averaging the three highest Δ*G*_open_ values per helix.

To understand sequence–cooperativity relationships at a more granular level, we examined how specific residues in individual proteins influenced opening cooperativity. We chose the proteins HHH_rd4_0518 and EEHEE_rd4_0871 as exemplars of low cooperativity (Fig. 3a–c). Both proteins adopt fully folded native structures consistent with their computationally designed models and predicted structures (Fig. 3d,e). Using our family-specific models, we identified double mutations predicted to increase opening cooperativity while preserving or increasing stability (Fig. 5a and Supplementary Fig. 10). Such mutations were predicted to be rare (only 4–6% of all possible mutations), making their identification a substantial prospective test of the models. In EEHEE_rd4_0871, the most frequently designed mutations were spread across the whole protein but, in HHH_rd4_0518, proposed mutations mainly clustered on the unstable C-terminal helix (Fig. 5a). We selected 70 top-ranking and 70 random double mutants for each wild type to evaluate machine-learning-guided design.

We experimentally analysed these 280 variants in small libraries and successfully measured opening energy profiles for 38 HHH_rd4_0518 variants (20 designed, 18 random) and 80 EEHEE_rd4_0871 variants (54 designed, 26 random). Designed variants typically showed increased opening cooperativity, although often at the cost of lower global stability (Fig. 5b). Still, five HHH_rd4_0518 variants (four designed, one random) and 14 EEHEE_rd4_0871 variants (12 designed, 2 random) achieved improvements in both properties (Fig. 5c,d). In HHH_rd4_0518, mutations at Arg35, Gly45 and Arg39 (all in the unstable C-terminal helix) repeatedly yielded increases in both properties, as did mutations at Lys21, His41 and Lys31 in EEHEE_rd4_0871 (Fig. 5c and Supplementary Fig. 11). In HHH_rd4_0518, G45L probably stabilizes the C terminus through new hydrophobic interactions, whereas R35D/E converts an unfavourable interaction with the helix backbone dipole^58^ into a favourable one (Fig. 5e). In EEHEE_rd4_0871, K31L probably stabilizes the hydrophobic core, E36V increases the strand propensity in the unstable C-terminal hairpin and K21I/L introduces new hydrophobic contacts between the helix and the unstable hairpin, similar to those present with the N-terminal hairpin (Fig. 5e).

To map changes in fluctuations at the residue level, we analysed one variant of each protein using HDX NMR (Fig. 5d,f). In EEHEE_rd4_0871_K31L_E36V, we observed increased stability at the C-terminal end of the helix and in several residues of the unstable C-terminal hairpin, although most of the unstable hairpin still exchanged too quickly to resolve (Supplementary Fig. 12). In HHH_rd4_0518_R35D_G45L, the mutations stabilized the entire protein, with the greatest stabilization in the least-stable helix α3 (+1.2 kcal mol^−1^ in α3, by comparing the three most stable residues between the mutant and wild type, compared with +0.9 for α2 and +0.6 for α1; Fig. 5f). This increased stability in the most labile segment explains the improved opening cooperativity observed by mHDX-MS.

Overall, these results illustrate how residues in unstable segments (for example, R35D and G45L in HHH_rd4_0518) and outside them (such as Glu11 in HHH_rd4_0518 or Lys21 and Lys31 in EEHEE_rd4_0871) can modulate local stability. As expected from the modest correlations, the machine learning results were not quantitatively accurate. However, they improved our exploration of sequence space by identifying rare mutations that could increase opening cooperativity while maintaining folding stability, with improved success compared with random mutations. This illustrates the potential for rational data-driven engineering of protein energy landscapes.

## Discussion

It is widely appreciated that proteins are constantly in motion, sampling numerous conformational states across their energy landscapes. Yet the energetic details of these states—and the sequence features mediating them—remain almost entirely unclear. The mHDX-MS method makes it possible to experimentally analyse these fluctuations on a far larger scale than was previously possible, enabling new approaches to understand and predict fluctuations across sequence space. Several key findings emerge. First, our experiments revealed hidden variation in energy landscapes across 3,590 designed and natural domains, including substantial variation between related sequences. In fact, within-family variation due to sequence differences often exceeded the average difference between fold families (Fig. 2e). Second, we found that low opening cooperativity domains in our set often had entire pieces of secondary structure that were much less stable than the overall fold (Fig. 3). These domains were not especially unusual: they represent the lowest 25% percentile (HHH_rd4_0518), 22% percentile (EEHEE_rd4_0871) and 6% percentile (LysM_0873) opening cooperativity scores in their families. Hundreds of domains in our set showed similarly low opening cooperativity, which is largely invisible to methods that predict native protein structures. Third, we found that global stability and local stability were partially uncoupled even in these small, compact domains: the domains with the highest folding stability (Δ*G*_unfold_) in our set were not necessarily the most stable throughout their entire structures, as indicated by Δ*G*_open_ distributions that ‘cross over’ in Figs. 2d and 3b. Finally, we found that conformational fluctuations remain challenging to predict even given substantial experimental data. Although we found general protein properties with statistically significant correlations with cooperativity, our best models could predict only a limited fraction (16–24%) of the variance in opening cooperativity. Given experimental noise, we would expect perfect models to show correlations of *R*^2^ = 0.74–0.78, indicating how much remains to be discovered.

Our approach has important limitations. Our inferred rates depend on approximations about back exchange and the validity of combining data measured at different pH. Although our HDX NMR and cDNA display proteolysis measurements, data quality filters and experimental reproducibility indicate that our high-throughput measurements are reliable, automated data processing can still introduce inaccuracies. To verify individual results, all raw MS data, extracted signals for each protein and Bayesian fits are provided (Data availability and Supplementary Table 1 (dataset 8)), along with quality metrics for each step. The conformational details of our observed partially open states also remain unknown, and our multiplexed measurements do not localize the high- and low-stability segments of each domain. Resolving these details remains a key next step, especially for the hundreds of low-cooperativity domains identified by our large-scale analysis.

Despite these limitations, we expect multiplexed HDX-MS to transform our ability to measure, predict and model protein conformational fluctuations. Improved MS technology will increase throughput even further. Larger domains should be amenable to our approach as well^4^. Combining our approach with bottom-up or top-down fragmentation strategies^31,32,60,61^ should enable library-scale measurement with enhanced spatial resolution of conformational fluctuations. Profiling conformational fluctuations at scale will also reveal new links between these high energy states and protein function, aggregation, immunogenicity and other critical properties. These fluctuations remain difficult to predict computationally^62,63^, and large-scale mHDX-MS data offer a new route to directly optimize physics-based models^63^, machine learning potentials^64–66^ and generative AI approaches^25^ to accurately model energy landscapes. Large-scale measurements, computational modelling and machine learning make a powerful combination that have already transformed our understanding of protein native states^56^. Multiplexed HDX-MS offers a powerful approach for mapping the far larger space of non-native states to empower data-driven modelling, discover new biology and accelerate protein engineering.

## Methods

### Library design

The initial set of 15,715 domain sequences was organized into five batches and further divided into 18 libraries (mix 1–4, libraries 1 and 4; libraries 7–15; and mutants 2–4): (1) mix 1–4: de novo designed ααα, βαββ and ββαββ sequences^21^; (2) libraries 1 and 4: de novo designed αββα proteins^11^; (3) libraries 7–14: natural domains from the Pfam database, including LysM, PASTA, WW, SH3, pyrin and cold-shock; (4) library 15: PDB-derived monomeric proteins devoid of cysteine residues and metal cofactors; (5) mutant libraries containing single and double mutants from EEHEE_rd4_0871 and HHH_rd4_0518 low-cooperativity proteins. Sequences were randomly assigned to libraries within each batch, ensuring a minimum mass difference of 50 ppm between nearest-neighbour sequences for mass spectrometry compatibility (except library 15 where two sequences are 36 ppm apart). After SUMO cleavage (see below), all proteins begin with the dipeptide HM (the scar from the NdeI ligation). Some sequences were modified with C-terminal padding (G, S, GG or GS) to optimize mass spacing. All sequences were reverse-translated and codon-optimized for *E. coli* using DNAworks (v.2.0)^68^. To standardize amplification efficiency, a ‘GGS’ sequence was appended after the stop codon. Oligo libraries encoding the original 15,715 sequences were purchased from Agilent Technologies, while the 280 designed mutations were sourced from Twist Bioscience.

### Cloning of Twist oligo libraries into the pGR02 plasmid

Oligo libraries were resuspended and amplified by quantitative PCR (qPCR) for restriction enzyme cloning. A preliminary qPCR run determined optimal amplification cycles, preventing overamplification by terminating reactions at around 50% of maximum fluorescence intensity. Purified qPCR products were digested with XhoI and NdeI and ligated into the pGR02 plasmid, which encodes an N-terminal 10×His-SUMO tag. Ligated constructs were electroporated into 10-β electrocompetent *E. coli* (New England Biolabs) and recovered in SOC medium at 37 °C for 1 h before plating onto selective MDAG-11 + B1 + kanamycin agar plates^69^. Serial dilutions determined transformation efficiency, and all colonies were pooled to maximize sequence diversity. Plasmid DNA was extracted from pooled cultures using the QIAprep Spin Miniprep Kit (Qiagen).

### Library expression and purification

Each library’s plasmid pool (5 μl) was electroporated into 25 μl BL21(DE3) electrocompetent *E. coli* (Sigma-Aldrich), recovered in SOC medium (1 ml, 37 °C, 1 h), and plated onto selective MDAG-11 + B1 + kanamycin agar plates. Colonies were pooled and used to inoculate 2–4 l of LB broth with 50 μg ml^−1^ kanamycin. Cultures were grown at 37 °C until an optical density at 600 nm (OD_600_) of 0.6, then induced with 1 mM IPTG and incubated at 16 °C overnight (~16 h). Cells were collected by centrifugation and resuspended in lysis buffer (20 mM Tris, 500 mM NaCl, 30 mM imidazole, 0.25% CHAPS, 1 mg ml^−1^ lysozyme, 10 U ml^−1^ Benzonase, 1× Pierce protease inhibitor cocktail, pH 8.0). Sonication (QSonica, 5 min total, 60% amplitude, 1 min on/off cycles) was followed by centrifugation (12,500*g*, 30 min, 4 °C; repeated at 14,000*g* for clarification). The soluble fraction was purified through Ni-NTA agarose gravity columns (Qiagen). After washing with buffer (20 mM Tris, 500 mM NaCl, 30 mM imidazole, 0.25% CHAPS, 5% glycerol, pH 8.0), proteins were eluted (20 mM Tris, 300 mM NaCl, 500 mM imidazole, 5% glycerol, pH 8.0). Eluted proteins were dialysed overnight into PBS, and SUMO tags were cleaved using a 1:100 molar ratio of ULP1 (4 °C, ~20 h). A second Ni-NTA purification removed SUMO and ULP1, collecting cleaved proteins in the flow-through. Proteins were concentrated (3 kDa Amicon Ultra filters) and further purified by Superdex 75 10/300 GL size-exclusion chromatography (Cytiva) on the NGC FPLC system (Bio-Rad). The monomeric fractions were pooled, reconcentrated, filtered (0.22 μm Millex-GP filter), flash-frozen in liquid nitrogen and stored at −80 °C until use.

### Labelled protein expression and purification for NMR analysis

We selected 13 proteins for individual expression, purification and NMR analysis. The DNA sequences were codon-optimized for *E. coli* and cloned into pET-28a(+) (thrombin cleavage site) from Twist Biosciences or pET-28a(+)-TEV from GenScript. The plasmids were transformed into chemically competent BL21(DE3) cells. A small starter culture (5 ml) was inoculated in LB Miller broth with 50 μg ml^−1^ kanamycin and grown overnight at 37 °C, 220 rpm. The starter culture (25 μl) was then diluted into 50 ml of labelled M9 medium (42 mM Na_2_HPO_4_, 22 mM KH_2_PO_4_, 8.6 mM NaCl, 8.6 mM 15NH_4_Cl (Cambridge Isotope), 11 mM d-glucose (^13^C, Cambridge Isotope), 1 mM MgSO_4_, 0.2 mM CaCl_2_, 0.15 mM thiamine, 1% (v/v) trace elements (3 mM FeCl_3_, 0.37 mM ZnCl_2_, 0.074 mM CuCl_2_, 0.042 mM CoCl_2_·H2O, 0.162 mM H_3_BO_3_, 6.84 mM MnCl_2_·H_2_O)) with 50 μg ml^−1^ kanamycin and grown overnight at 37 °C, 220 rpm. Larger cultures of M9 medium were inoculated with overnight M9 small culture (50 ml per 1 l) and grown at 37 °C, 220 rpm to OD_600_ of around 0.6. Expression was induced with 0.5 mM IPTG, and cells were incubated at 16 °C overnight (around 16–18 h). Cells were collected, resuspended in lysis buffer (20 mM Tris, 500 mM NaCl, 30 mM imidazole, 0.25% CHAPS, pH 8.0, 1 mg ml^−1^ lysozyme, 10 U ml^−1^ Benzonase, 1× Pierce protease inhibitor EDTA-free) and lysed by sonication. The lysates were clarified by centrifugation (13,000*g*, 30 min). Proteins were purified by immobilized metal affinity chromatography (IMAC) using Ni-NTA agarose. The column was washed with buffer (20 mM Tris, 500 mM NaCl, 30 mM imidazole, 0.25% CHAPS, 5% glycerol, pH 8.0), and proteins were eluted in elution buffer (20 mM Tris, 300 mM NaCl, 500 mM imidazole, 5% glycerol, pH 8.0). Eluted proteins were dialysed into buffer (50 mM Tris, 200 mM NaCl, 5% glycerol, pH 8.0) using Pur-A-Lyzer dialysis tubes (Sigma-Aldrich). His-tags were cleaved using either TEV protease (produced in-house, pRK793 plasmid; Addgene, 8827) or thrombin CleanCleave kit (Sigma-Aldrich), depending on the construct. TEV protease was added at a protease:target protein ratio of 1:10 with 0.5 mM DTT and incubated overnight at room temperature. Thrombin cleavage followed the manufacturer’s protocol, incubating overnight at room temperature. A second IMAC Ni-NTA purification was performed to remove the tag and uncleaved protein. Proteins were further purified by size-exclusion chromatography using a Superdex 75 10/300 column in phosphate-buffered saline. Monomeric fractions were identified based on elution profiles of a standard mixture (BSA, ovalbumin, ribonuclease A, aprotinin and vitamin B12), pooled, and concentrated using Amicon Ultra-4 centrifugal filters. The protein concentration was determined using the Pierce BCA assay (Thermo Fisher Scientific).

### NMR structure determination

NMR spectra for HHH_rd4_0518, EEHEE_rd4_0871 and EEHEE_rd4_0642 structure calculations were acquired at 288 K on Bruker spectrometers operating at 600 and 800 MHz, equipped with TCI cryoprobes with the protein buffered in 20 mM sodium phosphate (pH 7.5, 150 mM NaCl) at concentrations of 0.5 to 1 mM. Resonance assignments for ^15^N/^13^C-labelled proteins were determined using FMCGUI^70^ based on a standard suite of 3D triple- and double-resonance NMR experiments collected as described previously^71^. All 3D spectra were acquired with non-uniform sampling in the indirect dimensions and were reconstructed by the multi-dimensional decomposition software qMDD^72^, interfaced with NMRPipe^73^. Peak picking was performed manually using NMRFAM-Sparky^74^. Torsion angle restraints were derived from TALOS+^75^. Automated NOE assignments and structure calculations were conducted using CYANA (v.2.1)^76^. The best 20 out of 100 CYANA-generated structures were refined with CNSSOLVE^77^ by performing a short restrained molecular dynamics simulation in explicit solvent^78^. The final 20 refined structures comprise the NMR ensemble. Structure quality scores were performed using Procheck analysis^79^ and the PSVS server^80^.

### HDX NMR analysis

NMR HDX rates were measured for 13 proteins. A detailed overview of the experimental conditions, including buffer composition, temperature, pH and protein constructs, is provided in Supplementary Fig. 5 and Supplementary Table 1 (dataset 4). Exchange experiments were conducted at 600 MHz by tracking the decay of amide peak intensities in ^1^H-^15^N HSQC spectra over a 24 h period. Proteins were lyophilized in their respective analysis buffers, and exchange was initiated by dissolving them in an equivalent volume of D_2_O. Each HSQC timepoint required approximately 5 min for acquisition, with the first measurement occurring around 5 min after exchange initiation. Peak intensity data were fitted to a single exponential decay, and opening free energies were derived from these rates as described previously^67,81,82^.

### mHDX-MS analysis

Library samples (0.1–1 mg ml^−1^) were diluted 1:9 into deuterated buffer (95% D_2_O), either 25 mM MES (for pH_read_ ≈ 6) or 25 mM bicine (for pH_read_ ≈ 9). At specified incubation times, 95 µl of the exchange solution was mixed with 25 µl of quench buffer (0.5 M Gly–HCl, pH 2.1–2.3). All sample handling was fully automated using a PAL3 LEAP HDxTool: incubation in D_2_O buffer took place in a chamber at 20 °C, while quenching was performed in a chamber maintained at 0 °C. All buffers were individually adjusted before the experiment so that final pH_read_ values were 6.0 ± 0.05 (MES buffer) or 9.0 ± 0.05 (bicine buffer) and quench conditions were pH_read_ = 2.45 ± 0.05 after diluting with the sample. The quenched samples were then analysed on the Waters Synapt G2-Si Q–TOF mass spectrometer equipped with a Waters HDX Manager. Chromatography separation was performed at 0 °C on a 1 mm × 100 mm BEH C4 column (300 Å, 1.7 µm particles) using a 30 min gradient, with the entire system maintained at 0 °C to minimize back exchange. Each library was measured in two batches—one at pH 6 and one at pH 9—collecting 32 timepoints (log-spaced from 25 s to 24 h) plus three undeuterated replicates distributed throughout the experiment. All MS runs were performed in MS1-only mode at 1 scan per second. Every 10 s, one scan of sodium formate solution was collected for use in post-processing calibration.

### The computational pipeline for mHDX-MS

We developed a computational pipeline for mHDX-MS analysis that is organized into two complementary repositories: mhdx_pipeline (available at https://github.com/Rocklin-Lab/mhdx_pipeline), which handles the processing of LC–IMS-MS data (converted from mzML) through protein identification, signal decomposition through tensor factorization and the assembly of time-dependent mass distributions using a path optimization module; and hdxrate_pipeline (available at https://github.com/Rocklin-Lab/hdxrate_pipeline), which applies back-exchange correction, performs rate fitting via Bayesian optimization and converts these rates into opening free energies. These pipelines are implemented using Snakemake^83,84^, enabling reproducible, scalable and parallel processing across compute clusters, thereby enhancing workflow efficiency and facilitating easy re-execution of the entire analysis. We also made available mhdx_analysis (https://github.com/Rocklin-Lab/mhdx_analysis) to provide readers with useful code for post processing of mHDX-MS results. Our entire codebase is freely available under CC BY 4.0 license. Below we describe the main components of both pipelines.

### Protein identification

We used IMTBX and Grppr to extract the set of protein-like isotopic clusters (ICs) to allow for automated processing of the undeuterated samples^85^. We use the default parameters except that we do not apply automatic mass correction. Calibration was performed as described below. Protein identification is performed solely based on the MS1 intact mass, with our library designed so that each protein exhibits a unique mass (>50 ppm, except library 15, for which two sequences were within 36 ppm). In our pipeline, we have three sequential quality-control steps for protein identification: (1) mass error filtering: proteins are filtered based on their absolute protein monoisotopic mass error <10 ppm of theoretical values; (2) isotopic distribution matching: identified proteins are further filtered based on a dot product of experimental and theoretical isotopic distributions (idotp), with a threshold idotp > 0.98; (3) we also implemented a false-discovery rate (FDR) estimation and control: a target-decoy strategy to estimate and control for FDR. In this strategy, we generate a decoy sequence database that is twice the size of the initial target library. Decoy sequences are sequences randomized within the expected mass range of target sequences (±50 Da) and are designed to be at least 50 ppm apart from their nearest mass neighbour. At this stage, proteins passing the mass error and idotp thresholds often include an expected greater number of target sequences compared to decoy sequences. This prefiltered dataset is then used for FDR estimation. We trained a regularized logistic regression model to discriminate between identifications from the target database and those from the decoy database. We extract 36 features, including charge-based features, ppm deviation, idotp, intensity metrics and retention time (RT) residual errors. RT residual errors are calculated using a linear regression model trained on the target dataset to predict RT based on amino acid composition. The squared difference between the experimental and predicted RT is used as an additional descriptor for our logistic regression model. The trained logistic regression model outputs probabilities for both the target dataset and the held-out decoy set. At each probability threshold, we estimate the FDR as the ratio of decoys to targets above the threshold. For individual identifications, *q* values are calculated as the proportion of decoys relative to targets observed above the given probability threshold. Proteins are filtered based on a *q*-value threshold of 0.025, corresponding to an estimated FDR of 2.5%. In this work, steps 1 and 2 were applied as initial filters for downstream analyses, while step 3 served as a post-processing step. The regularized logistic regression model was trained using all library results (excluding mutant libraries) to derive a unified classification model. This model was then applied to mutant libraries, using the probability threshold (*P* = 0.803) observed to achieve an FDR of 2.5% in the training data.

### MS calibration

To ensure mass accuracy during the analysis, we implemented a lockmass calibration strategy using a reference compound. In this work, we used a solution with sodium formate (1:1:18 0.1 M NaOH:10% formic acid:acetonitrile) as a calibrant. In our pipeline, raw MS data files in .mzML format are parsed to extract individual scans and their respective RTs. For each scan, the experimental spectrum is filtered to retain peaks within a predefined mass-to-charge (*m*/*z*) tolerance of 100 ppm around theoretical reference masses. Filtered peaks with intensities exceeding 500 are grouped into chromatographic time bins (5 bins across a 30 min runtime). This allows localized analysis of reference peaks to account for time-dependent variations. For each reference *m*/*z* value, a Gaussian function is fitted to the experimental peaks in the filtered spectrum. Peaks with residual errors exceeding the defined 100 ppm tolerance or intensities below the threshold are excluded from calibration. For valid peaks, the observed *m*/*z* values are matched to the corresponding theoretical values. We build a linear regression curve to map observed *m*/*z* values to theoretical reference values. The calibration curve coefficients are stored and applied to correct all experimental *m*/*z* values within the dataset either at the stage of protein identification (see the ‘Protein identification’ section) or at the tensor extraction stage (see the ‘Tensor extraction’ section).

### Tensor extraction

To isolate individual protein signals from our LC–IMS-MS data, we represented each experiment as a 3D tensor spanning RT, drift time (DT) and *m*/*z* dimensions. For each protein and charge state at each timepoint, we extracted a sub-tensor centred on the protein’s observed RT and DT (and window of ±0.4 min for RT, ±6% of DT centre for DT), plus *m*/*z* range corresponding to the expected isotopic envelope extended to the maximum possible deuteration. RT and DT centres were empirically defined by averaging what was observed across undeuterated replicates to account for experimental variability. We applied *m*/*z* calibration corrections before extracting each sub-tensor to refine mass accuracy according to the strategy described above.

### Tensor factorization for signal decomposition

We next performed iterative factorization using a rank-decomposition approach (such as non-negative matrix factorization in multiple dimensions; schematics are provided in Supplementary Fig. 1a)^86^. We smoothed the sub-tensor in the RT and DT dimensions using small Gaussian kernels (*σ*_RT_,*σ*_DT_ = (3,1)) to improve the signal-to-noise ratio. Beginning with an initial guess of *k* = 5 factors, if the minimum correlation across RT, DT or *m*/*z* dimensions between any pair of factors exceeded a specified threshold of 0.17, we considered the data over-factorized and reduced *k* accordingly. This adaptive process continued until factors were sufficiently distinct. If factors were initially sufficiently distinct, we increased *k* until we observed over-factorization, keeping the previous iteration. Each resulting factor was further filtered by their Gaussian fit quality in RT and DT (*R*^2^ ≥ 0.90) to ensure that they represented coherent elution or drift profiles. Next, we examined each factor for multiple ICs. We projected each factor’s *m*/*z* dimension to create an integrated mass profile, then used a peak-finding algorithm to locate individual clusters. If more than one cluster was detected, we split the factor accordingly, treating each IC as a distinct signal component. This procedure enabled us to separate closely spaced isotopic envelopes and reduce noise-induced over-segmentation, ultimately yielding the collection of signals for downstream analyses.

### PO for assembly of time-course mass distributions

After extracting and resolving signals for each protein and timepoint, we assembled a consistent, time-resolved mass profile for each protein by selecting the most plausible IC at each timepoint using the path optimizer (PO) module in our pipeline. The resulting path spans the undeuterated (initial) to increasingly deuterated (late) states. All ICs—regardless of their charge state—are analysed together by converting each *m*/*z* signal into a neutral mass representation (that is, baseline-integrated mass distribution). Before path optimization, we derive nine quantitative features for each IC: (1) RT/DT errors: we compute the deviation in each IC’s RT (s) and DT (percentage) relative to the undeuterated reference signal; (2) RT/DT profile similarity: the cross-correlation between each IC’s elution profile (RT or DT) and that of the undeuterated reference quantifies overall shape similarity; (3) peak error: we compare the observed mass to a theoretical isotopic envelope, penalizing large discrepancies from the expected mass; (4) full width at half maximum (FWHM) deviation: we calculate the FWHM difference relative to the undeuterated reference; (5) intensity deviation: each IC’s integrated intensity is compared to the baseline intensity of the undeuterated protein; unusual gains or losses raise suspicion; and (6) neighbour correlation: if a protein exhibits multiple charge states at a given timepoint, we compute a correlation across *m*/*z* and RT dimensions to ensure they represent the same underlying species. ICs lacking other charge states are assigned zero correlation. (7) Signal noise estimation: the integrated mass distribution is fitted to a Gaussian, higher noise or incomplete peaks indicate lower quality, and result in higher values.

To reduce the number of low-quality or redundant ICs before optimization, we apply two complementary filtering strategies: (1) user-defined thresholds: each IC is evaluated against empirical cutoffs for multiple features (for example, maximum RT/DT errors, minimum RT/DT cross-correlation, maximum mass error). ICs exceeding these thresholds (or failing to meet minimum criteria) are excluded outright, ensuring that only clusters within typical experimental bounds proceed to the next stage. (2) Weak Pareto dominance: within each timepoint, we compare ICs that have similar baseline-integrated neutral masses. If one IC is strictly worse than another on multiple metrics (for example, higher RT/DT errors, lower RT/DT profile fits, greater peak error), it is Pareto-dominated and removed. This pruning further refines the set of plausible ICs by discarding those demonstrably inferior across key features.

After prefiltering, we create an array of hypothetical deuteration trajectories, each characterized by a starting fractional uptake and a slope parameter in logarithmic space. For each trajectory, our algorithm selects the best undeuterated IC (based on the dot product with the theoretical isotopic distribution, idotp) and next proceeds across subsequent timepoints, selecting the IC of which the baseline-integrated mass is closest to the trajectory’s expected deuteration level. Each of these initial sampled paths undergoes a greedy local refinement search where we iteratively swap out a single IC at a time if a replacement lowers the path score. Specifically, at each timepoint, we consider all ICs and check whether substituting any single IC yields a better overall path. This iterative process continues until no single substitution can further reduce the path score. Each potential time-course path is evaluated using a multidimensional scoring function that sums several penalty terms that capture low data quality or physically implausible behaviour. The physically implausible behaviour is assessed for whether mass addition between consecutive timepoints does not decrease (no negative uptake) and does not change abruptly (average deuterium uptake does not become faster with time). From the final paths, after the greedy swaps, we choose the one with the lowest score as the winner. This path represents the most coherent set of ICs from undeuterated through late timepoints.

Before downstream analysis, the pipeline runs the PO module in a temporary mode, generating a set of best-fit paths from all proteins to collect statistics (for example, typical RT/DT error, baseline mass deviations). These statistics are aggregated to define empirical thresholds (for example, 2 s.d. above/below the mean) that exclude outlier signals. For each protein, the pipeline then repeats path optimization as described above with these thresholds in non-temporary mode, ignoring candidate clusters failing the newly established criteria. This two-stage approach filters low-quality clusters and yields more robust final time courses. Final paths with a path score (PO total score) of lower than 50 were selected for rate fitting.

### Back-exchange correction in mHDX-MS

In HDX-MS, deuterated residues can back exchange to hydrogen during the quenching step and during LC (performed in a non-deuterated buffer). The level of back exchange varies from protein to protein (affecting all measurements of the same protein in a uniform way) and also varies timepoint to timepoint based on small differences in conditions (affecting all proteins in the sample in a uniform way). We correct all measurements using both timepoint-specific and protein-specific corrections to determine the original level of deuteration before any back exchange. Although different residues in each protein may back exchange at different rates, our model assumes a single overall back-exchange percentage for all residues in a given protein at a given timepoint (equation (3)).

#### Protein-specific back-exchange correction

We determine the percentage of back exchange for each protein based on the deuteration level observed in the longest timepoint (typically 24 h) when the total deuteration is no longer changing (that is, the protein achieved full deuteration and any missing deuteriums are the result of back exchange). Back- exchange percentages are computed separately for pH_read_ 6 experiments and pH_read_ 9 experiments for each protein. For proteins that do not reach full deuteration in pH_read_ 6 experiments, we estimate their pH_read_ 6 back exchange level based on an empirical linear correlation between back exchange measured at pH 6 (for other, fully deuterated proteins) and back exchange measured at pH_read_ 9 (for those same proteins; Extended Data Fig. 1a). Overall, protein-specific back exchange varied from 6 to 45%.

#### Timepoint-specific back-exchange correction

We identify fully deuterated proteins by checking whether their final five timepoints show ≤1 Da variation in mass (Extended Data Fig. 1b). Next, we check which subset of proteins is fully deuterated in the preceding timepoints by checking whether their centroid masses remain within 6% of that final mass. Owing to the diverse stabilities in our samples, there is typically a subset of unstable proteins that rapidly reach full exchange, serving as internal controls for timepoint-specific back exchange across all timepoints. If fewer than five such proteins are found at a given timepoint, we apply the average back exchange computed from later timepoints where enough fully deuterated proteins were found. The timepoint-specific back exchange correction (which varied from −5% to +4% across all timepoints) is added to each protein’s back-exchange value to determine backexchange(*s*,*t*) in equation (3).

### Inferring exchange rates from isotopic distributions

We sample a set of exchange rates *k*_HX_ (one rate per exchangeable residue) to obtain the posterior rate distribution that is consistent with the observed isotopic envelopes according to the model described in equations (3) and (4). The N-terminal two residues are excluded because these fully back exchange. For domains for which we combine data from pH_read_ 6 and pH_read_ 9, we also sample the time scaling factor which determines ‘effective pH_read_ 6 measurement times’ for all pH_read_ 9 measurements (Extended Data Fig. 1c–f). This allows for small deviations from a theoretical factor of 10^3^ due to pH-dependent changes in protein dynamics or charge effects on *k*_chem_.

The inputs to the model are the experimentally measured integrated mass distributions at each timepoint (non-zero intensity only), the corresponding timepoints, the theoretical isotopic distribution in the undeuterated state^87^, the estimated level of back exchange at each timepoint and the fraction of D_2_O in the exchange buffer. Next, we use Bayesian inference (through no-U-turn sampling (NUTS), as implemented in the numpyro package^88^) to infer the set of amide exchange rates ln(*k*_HX_). We define a hierarchical prior that first samples a slowest rate from a truncated normal centred near e^−7^ s^−1^ (with scale e^10^ and bounded below e^−15^), then linearly spaces a set of *N* log-rates from this slowest rate up to ln(e^10^). Each of the *N* log-rates is assigned a broad normal prior distribution (*σ* = 5), reflecting minimal prior knowledge (Fig. 1e). For each proposed set of rates, we compute a Poisson-binomial^89,90^ distribution of exchanges—adjusted by the inferred back exchange and deuterium fraction (equation (3))—to yield a convolved theoretical integrated mass envelope per timepoint. Discrepancies between these theoretical envelopes and the measured intensities are captured by a Gaussian distribution with a global noise parameter (drawn from an exponential prior, *σ* = 1). We run the Markov chain Monte Carlo algorithm with four parallel chains, using 100 warm-up iterations and 250 posterior draws in each chain. After sampling, we discard any problematic chains (for example, poor *R*-hat or chain-specific r.m.s.e.) and re-run if necessary. Finally, samples from all chains are combined, and each sample’s posterior rate distribution is sorted from slowest to fastest, yielding posterior distributions for each *i*th slowest rate (with *i* ranging from 1 to *N*) as shown in Fig. 1e.

### Chemical intrinsic rate approximation in mHDX-MS analysis

To convert our Bayesian-inferred exchange rates *k*_HX,*i*_ into residue opening free energies Δ*G*_open,i_, we must know each site’s intrinsic rate *k*_chem,*i*_ (equation (2)). However, each residue has a unique *k*_chem,*i*_ based on its local sequence context^67,91^, and our data do not reveal which measured rate is associated with which residue’s *k*_chem_. A straightforward approximation is simply to use the median (or mean) value among the intrinsic rates. To improve on this approximation, we examined the empirical relationship between *k*_chem,*i*_ and *k*_HX,*i*_ using site-resolved HDX NMR data on 11 proteins (all from Supplementary Fig. 5, except for double mutants HHH_rd4_0518_R35D_G45L and EEHEE_rd4_0871_K31L_E36V). We found a weak but significant trend across nearly all proteins whereby residues with slower *k*_HX,*i*_ also had slower *k*_chem,*i*_ (Supplementary Fig. 2a). We incorporated this trend into our estimation of *k*_chem,*i*_ using a simple scaling factor between normalized *k*_HX,*i*_ and normalized *k*_chem,*i*_:

$$\log \,{k}_{{\rm{c}}{\rm{h}}{\rm{e}}{\rm{m}},i}={\rm{m}}{\rm{e}}{\rm{d}}{\rm{i}}{\rm{a}}{\rm{n}}(\log \,{k}_{{\rm{c}}{\rm{h}}{\rm{e}}{\rm{m}}})+\alpha \times z\mathrm{-}{\rm{s}}{\rm{c}}{\rm{o}}{\rm{r}}{\rm{e}}(\log \,{k}_{{\rm{H}}{\rm{X}},i})\times {\rm{s}}{\rm{t}}{\rm{d}}(\log \,{k}_{{\rm{c}}{\rm{h}}{\rm{e}}{\rm{m}}})$$

where median(log *k*_chem_), *z*-score(log *k*_HX,*i*_) and std(log *k*_chem_) are computed separately for each protein, and a universal scaling factor *α* is used for all proteins. As a result, a residue with the average log *k*_HX,*i*_ for its protein (that is, *z*-score = 0) will be analysed using that protein’s median *k*_chem,*i*_, and faster or slower residues will be analysed using faster or slower *k*_chem,*i*_ based on the scaling factor *α*. Here, we used a scaling factor *α* = 0.38, which nearly minimizes the error between our inferred Δ*G*_open_ distributions and the Δ*G*_open_ distributions from NMR across all 11 proteins (this was computed using a smaller set of NMR proteins than our final set). This adjustment yields Δ*G*_open_ distributions that are more accurate than assuming the median *k*_chem_ for all positions (Supplementary Fig. 2b).

### Net charge correction to all Δ*G*_open_

To account for electrostatic effects on hydrogen exchange rates, we applied a correction to the estimated Δ*G*_open_ values based on the estimated net charge of each protein at pH 6 (as computed by the ProtParam module within the Biopython package^92,93^). It has been shown that negatively charged proteins have slower *k*_chem_ due to decreased local concentration of the hydroxide catalyst, and vice versa^94,95^, although these corrections are not explicitly considered in the exchange framework of ref. ^40^. Our data reproduced this electrostatic dependency. To empirically correct for this effect, we derived a linear adjustment that removes the dependency between net charge and Δ*G*_unfold_. For proteins with Δ*G*_unfold_ > 4 kcal mol^−1^, we found the nearly optimal correction coefficient of 0.12 kcal mol^−1^ per unit charge, which was then applied to all Δ*G*_open,*i*_ values.

### Δ*G*_unfold_ calculation

We derive Δ*G*_unfold_ as the average across the five more stable residues (averaging across 1–6 residues leads to highly correlated Δ*G*_unfold_ estimates; Supplementary Fig. 3a). When deriving Δ*G*_unfold_, we did not account for the contribution of the unfolded state *cis*–*trans* isomerization to the measured Δ*G*_unfold_^39,40^. Thus, all Δ*G*_unfold_ measurements are in reference to an unfolded state in which prolines have their native-state isomerization state, rather than a fully equilibrated unfolded state. However, analysis of proline isomerization states in AlphaFold 2 models (Supplementary Fig. 3b) indicates that *cis*-prolines are predominantly present in PASTA domains, with minimal occurrence in other protein families analysed (Supplementary Table 1 (dataset 3)). Thus, potential biases due to *cis*-prolines are largely confined to PASTA and do not substantially affect the other protein families examined. We also did not correct results for the small effects of *trans*-prolines.

### Detecting and filtering EX1 behaviour

Our pipeline is primarily designed for unimodal, EX2-like exchange profiles. It attempts to split multimodal or poorly fitted peaks into separate unimodal peaks, which is not ideal for analysing EX1 kinetics. We therefore do not recommend using this code in its current form to analyse EX1 data. Nonetheless, we implemented an automated procedure to identify and exclude these potential EX1-type profiles from our dataset. Under EX1 kinetics, backbone amides often exhibit an abrupt shift in their isotopic distribution, leading to characteristic anomalies in the experimental and fitted exchange profiles. (1) Width criterion: for each timepoint, we compared the observed isotopic distribution width to the width of the theoretical distribution from our EX2-only fit. We then computed the ratio of these widths across the exchange progress—specifically focusing on timepoints at which the majority of amides (80–90%) had undergone exchange. Samples were flagged as potential EX1 if two conditions were met: (1) the average width ratio in that progress window exceeded 1.25; and (2) the maximum ratio was greater than 1.35. Any domain meeting both thresholds at either pH 6 or pH 9 was classified as ex1_width_criteria. (2) Jump criterion: to capture large, discrete transitions in the exchange profiles, we tracked the difference between the centroid of the fitted distribution (EX2 assumption) and the maximum peak of the observed distribution over time. An exchange profile was labelled as ex1_jump_criteria if it displayed a shift exceeding a predefined range (from less than −2 Da to more than +2 Da) coupled with the transition happening below 90% overall exchange. These constraints were set to identify ‘jumps’ characteristic of EX1-like coordinated exchange rather than the smoother transitions expected under EX2 kinetics. Schematics on each criteria are provided in Extended Data Fig. 2a,b.

### Determining full deuteration and protein classification in mHDX-MS

For protein classification, we defined full deuteration as a delta mass of less than 2 Da between the back-exchange-corrected centroid masses measured at the last and fifth-to-last timepoints. Based on this criterion, our data are classified into four groups: (1) unmeasurable proteins, for which full deuteration occurs too early for accurate exchange rate modelling. We also classify a unmeasurable protein if any of following conditions are met: slowest rate constant greater than 10^−4^ s^−1^, Δ*G*_unfold_ value is lower than 2 kcal mol^−1^ or fewer than 20% of residues present measurable exchange rates (*k*_HX_ < 10^−3.45^ s^−1^); (2) proteins that achieve full deuteration in the pH 6 experiment; (3) proteins that only reach full deuteration in the pH 9 experiment—requiring the integration of data from both pH 6 and pH 9; and (4) proteins that do not reach full deuteration even in the pH 9 experiment. Proteins belonging to groups (2) and (3) are subsequently referred to as measurably stable.

### Filtering low-quality data

To maximize the reliability of our mHDX-MS measurements, we applied a series of filtering criteria (Supplementary Fig. 13). First, low-quality identifications were removed based on mass error (>10 ppm), isotopic distribution matching (idotp < 0.98) and *q* values (>0.025), and paths with a PO score above 50 were excluded (or above 40 for HHH_rd4_0518 and EEHEE_rd4_0871 mutants) (Supplementary Table 1 (dataset 1)). For all proteins that reach full deuteration, we require overall back exchange to remain below 45% (we do not consider back exchange for proteins in group 4 that do not reach full deuteration). For proteins fully deuterated under both conditions, we required that the back-exchange difference between pH 6 and pH 9 be less than 10%. Moreover, we assessed the consistency between the experimental and theoretical mass distributions (from the inferred rate constants) by requiring that 90% of timepoints have an r.m.s.e. lower than 0.3 normalized units (mass distributions are normalized so that the most intense isotopic peak has an intensity of one unit). This filtered dataset is available as Supplementary Table 1 (dataset 2). Finally, for each protein, the mass distribution path with the lowest PO score was selected as the representative opening energy distribution, and proteins exhibiting EX1 kinetics (Extended Data Fig. 2), unmeasurable protection or incomplete deuteration at pH 9 (Extended Data Fig. 5) were excluded from the measurably stable dataset (Supplementary Table 1 (dataset 3).

### Protein structure prediction

We predict the 3D structures of the proteins from their amino acid sequences using AlphaFold 2^56^. The protein sequences, provided in FASTA format, were used as input to the ColabFold implementation of AlphaFold 2^96^, which was run on Quest high performance computing facility at Northwestern University. The pipeline generated five models for each sequence, and the best-scoring model, based on the predicted pLDDT, was refined using amber. The best AlphaFold model for each sequence was further refined using the Rosetta Relax protocol^97^. This process aimed to optimize the predicted structures by minimizing their energy. When not explicitly mentioned otherwise, the relaxed structure was selected for downstream analysis (Supplementary Table 1 (dataset 9)).

### Deriving normalized cooperativity and family-normalized cooperativity

We first computed each protein’s average opening free energy (Δ*G*_avg_) as the average of all Δ*G*_open_ values, with unmeasurably fast residues set to a lower bound of 0 kcal mol^−1^. Here, Δ*G*_avg_ serves as a simplified proxy for the energies of partially open states on the energy landscape. We then built an empirical model of the typical average opening energy (Δ*G*_avg,expected_) for a given domain based on its global stability (Δ*G*_unfold_), its fraction of exchangeable backbone amides that donate hydrogen bonds (fxn_hb) and its net charge (netq). In our Bayesian regression model, we define the expected average free energy as

$${\Delta G}_{{\rm{a}}{\rm{v}}{\rm{g}},{\rm{e}}{\rm{x}}{\rm{p}}{\rm{e}}{\rm{c}}{\rm{t}}{\rm{e}}{\rm{d}}}=a\times {({\Delta G}_{{\rm{u}}{\rm{n}}{\rm{f}}{\rm{o}}{\rm{l}}{\rm{d}}}-b)}^{c}\times {({\rm{f}}{\rm{x}}{\rm{n}}{\rm{\_}}{\rm{h}}{\rm{b}})}^{d}+e\times {\rm{n}}{\rm{e}}{\rm{t}}{\rm{q}},$$

where the parameters *a*, *b*, *c*, *d* and *e* are sampled from non-informative priors (specifically, *a* distributed as (∼) normal(1, 3), *b* ∼ normal(0, 5), e ∼ normal(–1, 3), log(*c*) ∼ normal(–0.3, 2) with *c* = exp(log(*c*)) and *d* ∼ normal(–0.3, 2) with *d* = exp(log(*d*))), and the observation noise is modelled with *σ* ∼ exponential(1). We run Markov chain Monte Carlo using NUTS (as implemented in numpyro^88^) with 1,000 warm-up iterations and 1,000 samples to obtain the posterior distribution of these parameters. We then used the mean values of the posterior distributions for the parameters *a*–*e* (Supplementary Table 2) to compute Δ*G*_avg,expected_ for each domain. Finally, we define normalized cooperativity as the *z* score of the residual between each domain’s experimental Δ*G*_avg_ and its predicted value, Δ*G*_avg,expected_:

$${\rm{N}}{\rm{o}}{\rm{r}}{\rm{m}}{\rm{a}}{\rm{l}}{\rm{i}}{\rm{z}}{\rm{e}}{\rm{d}}\,{\rm{c}}{\rm{o}}{\rm{o}}{\rm{p}}{\rm{e}}{\rm{r}}{\rm{a}}{\rm{t}}{\rm{i}}{\rm{v}}{\rm{i}}{\rm{t}}{\rm{y}}=\frac{{\Delta G}_{{\rm{a}}{\rm{v}}{\rm{g}}}-{\Delta G}_{{\rm{a}}{\rm{v}}{\rm{g}},{\rm{e}}{\rm{x}}{\rm{p}}{\rm{e}}{\rm{c}}{\rm{t}}{\rm{e}}{\rm{d}}}}{\sigma ({\Delta G}_{{\rm{a}}{\rm{v}}{\rm{g}}}-{\Delta G}_{{\rm{a}}{\rm{v}}{\rm{g}},{\rm{e}}{\rm{x}}{\rm{p}}{\rm{e}}{\rm{c}}{\rm{t}}{\rm{e}}{\rm{d}}})}$$

The hydrogen bonding fraction fxn_hb is defined as the fraction of backbone amides that donate hydrogen bonds to any acceptor (sidechain or backbone) in the molecule. The first two residues (the N terminus and the first amide) are excluded from both the numerator and the denominator because these residues fully back exchange during HDX-MS. Hydrogen bonds are determined using the Rosetta model^55,98^ based on AlphaFold 2 structural models that are subsequently relaxed with Rosetta. We call these predicted structures reference structures.

These reference structures are critical for the calculation of normalized cooperativity because they define our expectation for how many protected residues should be observed in a fully cooperative domain. Even if a domain’s native structure under our experimental conditions differs from the reference structure (which may occur because the reference structures are only computational predictions), normalized cooperativity is still computed based on the number of hydrogen bonds in the reference structure. In an extreme case, a protein might be substantially unfolded under our experimental conditions compared with the reference structure. For example, consider a protein with two subdomains where both are folded in the reference structure but only one subdomain is stable under our experimental conditions. Here, ‘cooperativity’ could be interpreted in different ways. If we only consider the fraction of the structure that is stably folded under our experimental conditions, this subdomain might behave in a perfectly two-state manner and be considered highly cooperative. However, the domain as a whole (including both subdomains) is not cooperative because one subdomain can unfold while the other remains folded. Our analysis (based on the reference structure) considers the domain as a whole. In this scenario, Δ*G*_avg_ will be low, because the residues in the unfolded subdomain (with low Δ*G*_open_) are included in the average. Δ*G*_avg,expected_ will be much higher, because Δ*G*_avg,expected_ is computed assuming a larger number of protected residues than are experimentally observed. The result is a low normalized cooperativity, indicating that the full domain (according to the reference structure) is relatively low cooperativity compared to other domains with similar stability in our dataset.

We also include a linear correction for net charge (netq) in the calculation of Δ*G*_avg,expected_. As discussed in the ‘Net charge correction to all Δ*G*_open_’ section, negatively charged proteins tend to exhibit slower *k*_chem_. As a result, negatively charged proteins tend to have a larger fraction of measurably stable residues compared to positively charged proteins (exchange is uniformly slower in negatively charged proteins regardless of conformational stability, so more residues can be measured). Although we correct for this bias by applying a charge correction to all Δ*G*_open_ measurements, there is still an uncorrected bias on the number of measurable residues. In fitting *k*_HX_, we use a prior distribution on *k*_HX_ that assumes that most residues are very unstable until they are shown to be stable in the data (Fig. 1e). As a result, the inferred *k*_HX_ (and Δ*G*_open_) end up being very different for residues that are barely slow enough to be measurable and residues that are barely too fast to be measured. Residues that are just barely measurable end up with Δ*G*_open_ of around 2 kcal mol^−1^, compared with residues that are slightly too fast to be measured, which often have inferred Δ*G*_open_ much lower than 0 kcal mol^−1^ (Fig. 1e). These very low opening energies are set to the lower boundary of Δ*G*_open_ = 0 kcal mol^−1^ when calculating Δ*G*_avg_. As a result, the bias towards more measurable residues in negatively charged proteins ends up biasing Δ*G*_avg_. Including netq in Δ*G*_avg,expected_ mitigates this bias to enable a more fair comparison of normalized cooperativity in negatively and positively charged proteins.

We derived two distinct cooperativity metrics. The normalized cooperativity metric is obtained by fitting the model to the entire dataset, while the family-normalized cooperativity metric is derived by fitting the model separately within each protein family (using only families with more than 50 examples in the measurably stable category). For both metrics, we report their normalized versions—computed by subtracting the mean and dividing by the s.d. of the residuals—yielding the normalized cooperativity and family-normalized cooperativity, respectively. A Jupyter Notebook for modelling cooperativity from new data, along with a script to derive cooperativity using our precomputed metrics, are available at GitHub (https://github.com/Rocklin-Lab/mhdx_analysis).

### PCA of opening energy profiles

PCA was applied to opening free-energy profiles derived from mHDX-MS measurements for proteins in Dataset_3_MeasurablyStable (3,590 proteins; Supplementary Table 1). For each protein, the opening energy distribution was represented as a one-dimensional vector and zero-padded to the maximum profile length across the dataset before PCA. No additional normalization for protein length, hydrogen-bonding or net charge was applied. PCA was performed either globally across all proteins or separately within individual protein families. For family-specific analyses, PCA was applied independently to proteins belonging to each family. Principal component scores were then correlated with global folding free energy (Δ*G*_unfold_) and family-normalized cooperativity using PCCs.

### Reproducibility of opening energy distributions in mHDX-MS

Starting from the filtered dataset (Supplementary Table 1 (dataset 2)), we assessed the reproducibility of measured opening energy distributions across independent mHDX-MS experiments. First, for proteins analysed in multiple libraries, if a protein was represented by more than one opening energy distribution within a given library, we selected the path with the lowest PO score as the representative for that library. Across all libraries, 127 opening energy distribution replicates were identified for 36 different proteins. For each protein, the replicate with the lowest PO score was designated as the reference distribution, and the MAD was computed between the reference and every other replicate for that protein in a different library, considering only the measurable opening energies (that is, excluding energies derived from rates outside the dynamic range). This MAD is shown in Fig. 1f.

We further observed that some proteins were detected at multiple RTs within a single experiment. To evaluate the reproducibility in this context, we started with the opening energy distribution corresponding to the lowest PO score as the reference. We then iteratively added additional distributions only if they were acquired at RTs at least 2 min apart from any previously selected distribution—thereby ensuring that the additional replicates reflected genuine distinct chromatographic events rather than minor experimental variability. Under these criteria, 180 proteins exhibited multiple distinct RT replicates (Supplementary Fig. 14a). Across these replicates, both the MAD for global stability (Δ*G*_unfold_) and the MAD computed over all measurable Δ*G*_open_ values were less than 0.5 kcal mol^−1^ (Supplementary Fig. 14b–d).

### Comparison between mHDX-MS and HDX NMR

We compared exchange rate and opening energy measurements obtained from HDX NMR with those derived from mHDX-MS. For the rate comparison, each HDX NMR log-rate was compared to a simulated mHDX-MS log-rate, which was derived by adjusting the measured mHDX-MS rates by the ratio of the median *k*_chem_ values between the two conditions. The overall agreement between the two methods was quantified by computing r.m.s.e. across all 511 *k*_HX_ measurements collected from 13 proteins in multiple HDX NMR conditions (Supplementary Fig. 5). For the opening energy distributions, each construct’s sorted average Δ*G*_open_ (averaged over conditions when multiple HDX NMR conditions were available for the same residue) was compared to the corresponding distribution from mHDX-MS. In cases in which the two distributions differed in length, both distributions were truncated to the length of the shorter distribution. We then computed a single overall r.m.s.e. across all 323 Δ*G*_open_ measurements from the 13 proteins.

### Feature extraction

Our feature extraction pipeline leverages both interpretable features and PLM embeddings. For the interpretable features, we computed a comprehensive set of predictors that include sequence-based descriptors generated with iFeature^99^ (for example, amino acid composition, grouped amino acid composition and composition–transition–distribution metrics), disorder predictors (ADOPT^57^ and flDPnn^100^) and structure-based features derived from AlphaFold2/Rosetta-relaxed models. These structure-based features encompass pLDDT scores from AlphaFold2, a set of Rosetta-based features extensively described previously^21^, solvent accessibility^101^, as well as custom-derived metrics such as side-chain contact scores, burial scores, hydrogen-bond metrics and an expanded set of secondary structural element-specific descriptors (for example, maximum hydrophobicity of helix-1, average pLDDT of helix-2). A complete set of scripts for computing these features is available at GitHub (https://github.com/Rocklin-Lab/mhdx_analysis/tree/main/scripts/feature_extraction). In total, we extracted 2,800 features common across folds and an additional 2,000–3,700 topology-specific features. Moreover, we extracted embeddings from PLMs—ESM2 (650 million parameters)^102^, Unirep^103^ and SaProt^104^—to capture contextual sequence information. For ESM2, we averaged the representations from layers 33–36 and flattened the result into an array of 2,560 features per protein sequence, while Unirep and SaProt yielded 1,900 and 1,280 features, respectively.

### Feature correlation analysis

PCCs were computed for all derived sequence and structural features with target variables, global stability (Δ*G*_unfold_), normalized cooperativity and family-normalized cooperativity within each protein topology. PCCs were computed using the pearsonr function from SciPy Stats. Bootstrapping was performed with 1,000 resamples to generate the 95% CI for PCCs. 10,000 random permutations of global stability (dg_mean), normalized cooperativity (normalized_cooperativity_model_global) and family-normalized cooperativity (normalized_cooperativity_pf) were generated for the HHH and EEHEE topologies. PCCs were calculated for each random permutation of the target variables with the derived sequence and structural features. The mean and 95% CIs were calculated from the distribution of PCCs of the random permutations with each feature and compared to the experimentally calculated PCC.

### Training of machine learning models

We implemented a machine learning pipeline to predict key protein properties—Δ*G*_unfold_ and family-normalized cooperativity—using regularized linear models (LassoCV and RidgeCV). We independently evaluated features derived from PLM embeddings (ESM-2, SaProt, Unirep) alongside interpretable features. Our pipeline systematically tests 20 model variations per feature set, comparing models that use the original features to those employing expanded features (including square, inverse and logarithmic transformations), and optionally applying Pearson correlation filtering at thresholds of 5%, 10%, 25% and 50% to remove weakly correlated features. Model training was performed using fivefold cross-validation across three independent random splits. To ensure reproducibility and biological relevance, we applied dynamic clustering with MMseqs2^105^ to group similar sequences. For each protein family, sequences were iteratively clustered using a range of minimum sequence identity thresholds (from 0.1 to 0.75), with clustering halted when the largest cluster dropped below 10% of the total sequence count, resulting in final thresholds of between 0.45 and 0.70 across families. These sequence clusters were then randomly assigned to the five folds, ensuring that each fold was as distinct as possible while maintaining balanced representation across protein families (PFs).

By contrast, a first-generation set of models was trained on an earlier version of the dataset where PLM embeddings were derived from ProteinMPNN^106^, ESM Inverse Folding 1 (ESM_IF1)^107^, Tranception^108^, ESM2 (with both 650 million and 3 billion parameters)^102^, and hybrid models combining ESM2 with ESM_IF1. For these first-generation models, sequences were assigned randomly to five folds without applying identity-based clustering. Despite this difference in preprocessing, the overall training strategy—using fivefold cross-validation across three independent random splits—remained consistent across both approaches.

In both cases, global models were trained on the full dataset and evaluated on individual PFs, while family-specific models were developed for PFs with more than 200 examples. Model performance was assessed using the *R*^2^ score aggregated across held-out sets, and final model selection was based on the highest mean *R*^2^ across the three splits to maximize generalization.

### Reporting summary

Further information on research design is available in the Nature Portfolio Reporting Summary linked to this article.

## Online content

Any methods, additional references, Nature Portfolio reporting summaries, source data, extended data, supplementary information, acknowledgements, peer review information; details of author contributions and competing interests; and statements of data and code availability are available at 10.1038/s41586-026-10465-z.

## Supplementary information

Supplementary Information Supplementary Figs. 1–14, Supplementary Tables 1 and 2 and Supplementary References.

Reporting Summary

Peer Review file

## Figures and Tables

**Extended Data Table 1:**
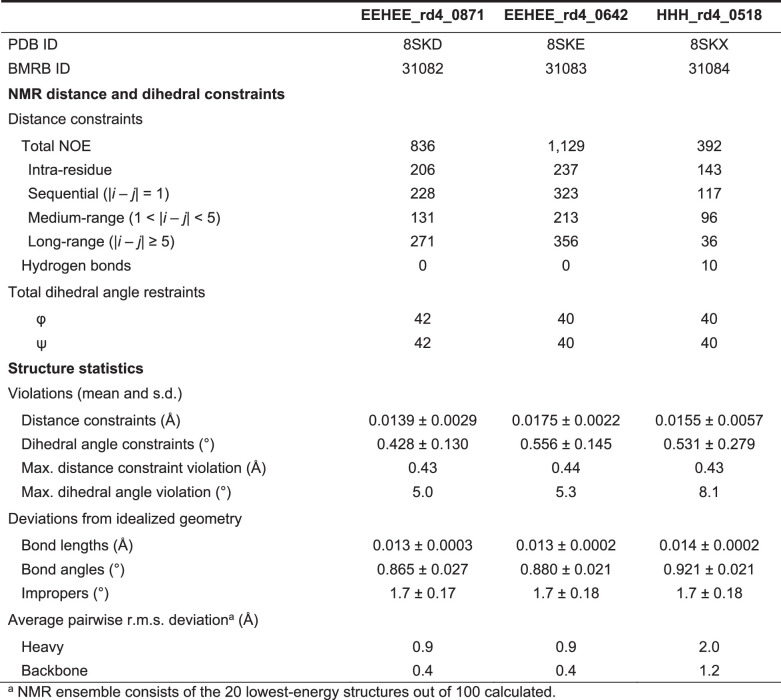
NMR and refinement statistics for miniproteins EEHEE_rd4_0871, EEHEE_rd4_0642 and HHH_rd4_0518

## Data Availability

All data supporting the findings of this study are available online (https://forms.gle/RwJwvfw6WN4gjXaD9). The MS proteomics data have been deposited to the ProteomeXchange Consortium via the PRIDE^109^ partner repository under dataset identifier PXD061702. Structural data have been deposited in the PDB under accession codes 8SKX, 8SKD and 8SKE.

## Code availability

The code for the analyses can be found at GitHub (https://github.com/Rocklin-Lab/mhdx_pipeline; https://github.com/Rocklin-Lab/hdxrate_pipeline; and https://github.com/Rocklin-Lab/mhdx_analysis).
