# Supplementary material for: Large-scale discovery, analysis and design of protein energy landscapes
Source: Nature. 2026 May 13;654(8120):1108–18. doi: 10.1038/s41586-026-10465-z (PMC13293878; doi:10.1038/s41586-026-10465-z)
Supplement: Supplementary file 1 — Supplementary Figs. 1–14, Supplementary Tables 1 and 2 and Supplementary References. [file 41586_2026_10465_MOESM1_ESM.pdf]

nature portfolio

https://doi.org/10.1038/s41586-026-10465-z

Supplementary information

Large-scale discovery, analysis and design of protein energy landscapes

In the format provided by the authors and unedited

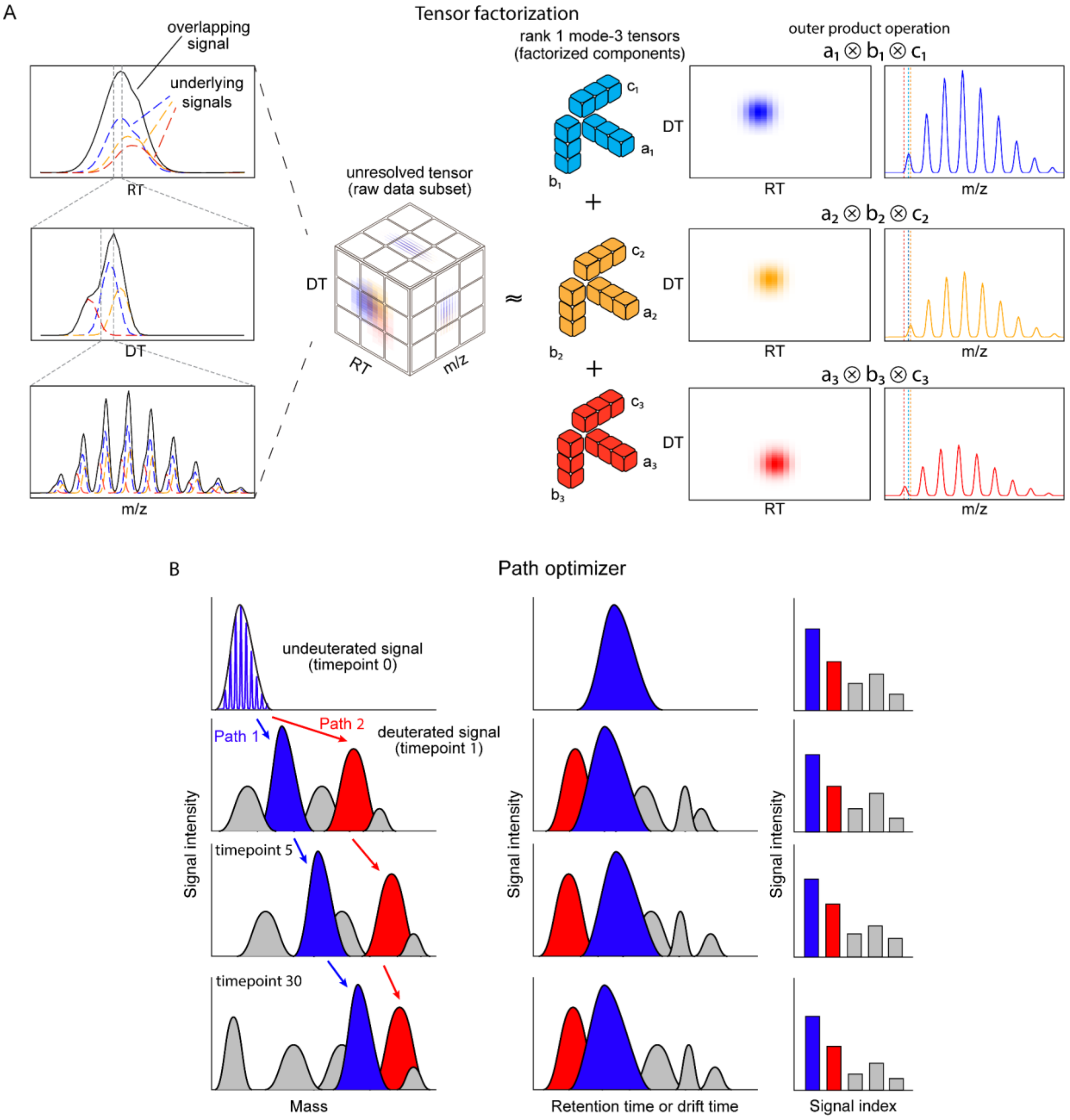

Figure S1: Tensor factorization and path optimization for mHDX-MS data. (A) Tensor factorization schematic**:** three underlying signals (blue, orange, red dashed lines) overlap in a single LC–IMS–MS experiment (black line). We represent the data as a 3D tensor spanning retention time (RT), drift time (DT), and m/z dimensions. By applying an iterative rank-decomposition approach (analogous to multi-dimensional nonnegative matrix factorization), each signal is approximated by a low-rank (rank-1) factor in each of the three modes. This effectively disentangles the individual signals, even when they overlap in multiple dimensions. (B) Path optimizer schematic: after deconvolving signals at each timepoint, the pipeline selects the most coherent set of isotopic clusters (ICs) to form a time-resolved mass profile. In this example, a "blue path" follows ICs that smoothly transition in mass, RT, and DT, whereas a "red path" includes

clusters with abrupt transitions and poorer agreement, leading to a higher penalty. The optimizer computes a multidimensional score (penalizing implausible changes in mass uptake, large RT/DT errors, etc.) and iteratively refines the path until no single substitution of an IC can further improve the overall score. The final “winning” path provides the most physically consistent deuteration trajectory from the undeuterated to the fully deuterated state.

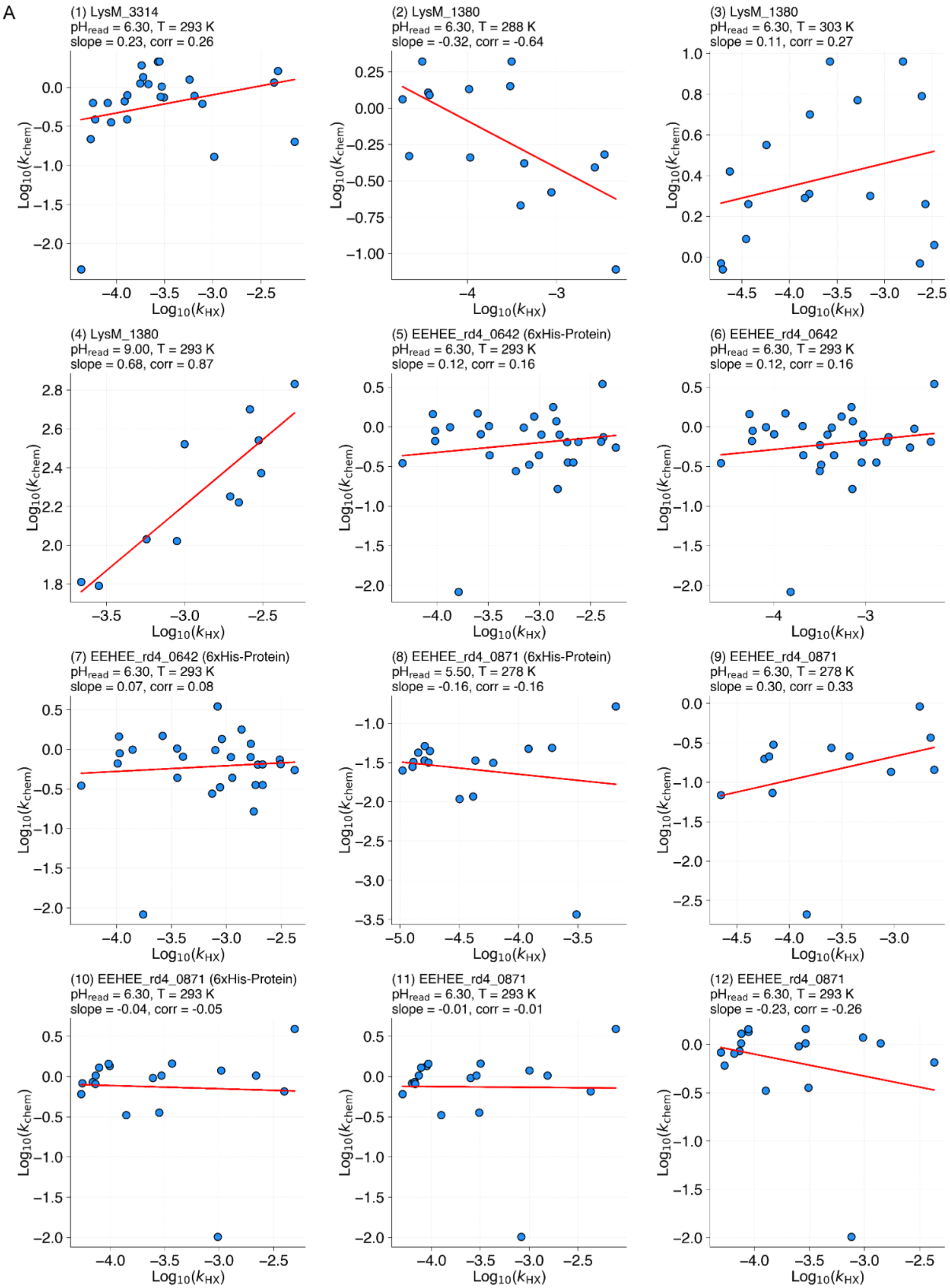
A
(1) LysM_3314
pH$_{read}$ = 6.30, T = 293 K
slope = 0.23, corr = 0.26
(2) LysM_1380
pH$_{read}$ = 6.30, T = 288 K
slope = -0.32, corr = -0.64
(3) LysM_1380
pH$_{read}$ = 6.30, T = 303 K
slope = 0.11, corr = 0.27
(4) LysM_1380
pH$_{read}$ = 9.00, T = 293 K
slope = 0.68, corr = 0.87
(5) EEHEE_rd4_0642 (6xHis-Protein)
pH$_{read}$ = 6.30, T = 293 K
slope = 0.12, corr = 0.16
(6) EEHEE_rd4_0642
pH$_{read}$ = 6.30, T = 293 K
slope = 0.12, corr = 0.16
(7) EEHEE_rd4_0642 (6xHis-Protein)
pH$_{read}$ = 6.30, T = 293 K
slope = 0.07, corr = 0.08
(8) EEHEE_rd4_0871 (6xHis-Protein)
pH$_{read}$ = 5.50, T = 278 K
slope = -0.16, corr = -0.16
(9) EEHEE_rd4_0871
pH$_{read}$ = 6.30, T = 278 K
slope = 0.30, corr = 0.33
(10) EEHEE_rd4_0871 (6xHis-Protein)
pH$_{read}$ = 6.30, T = 293 K
slope = -0.04, corr = -0.05
(11) EEHEE_rd4_0871
pH$_{read}$ = 6.30, T = 293 K
slope = -0.01, corr = -0.01
(12) EEHEE_rd4_0871
pH$_{read}$ = 6.30, T = 293 K
slope = -0.23, corr = -0.26
$Log_{10}(k_{chem})$
$Log_{10}(k_{HX})$

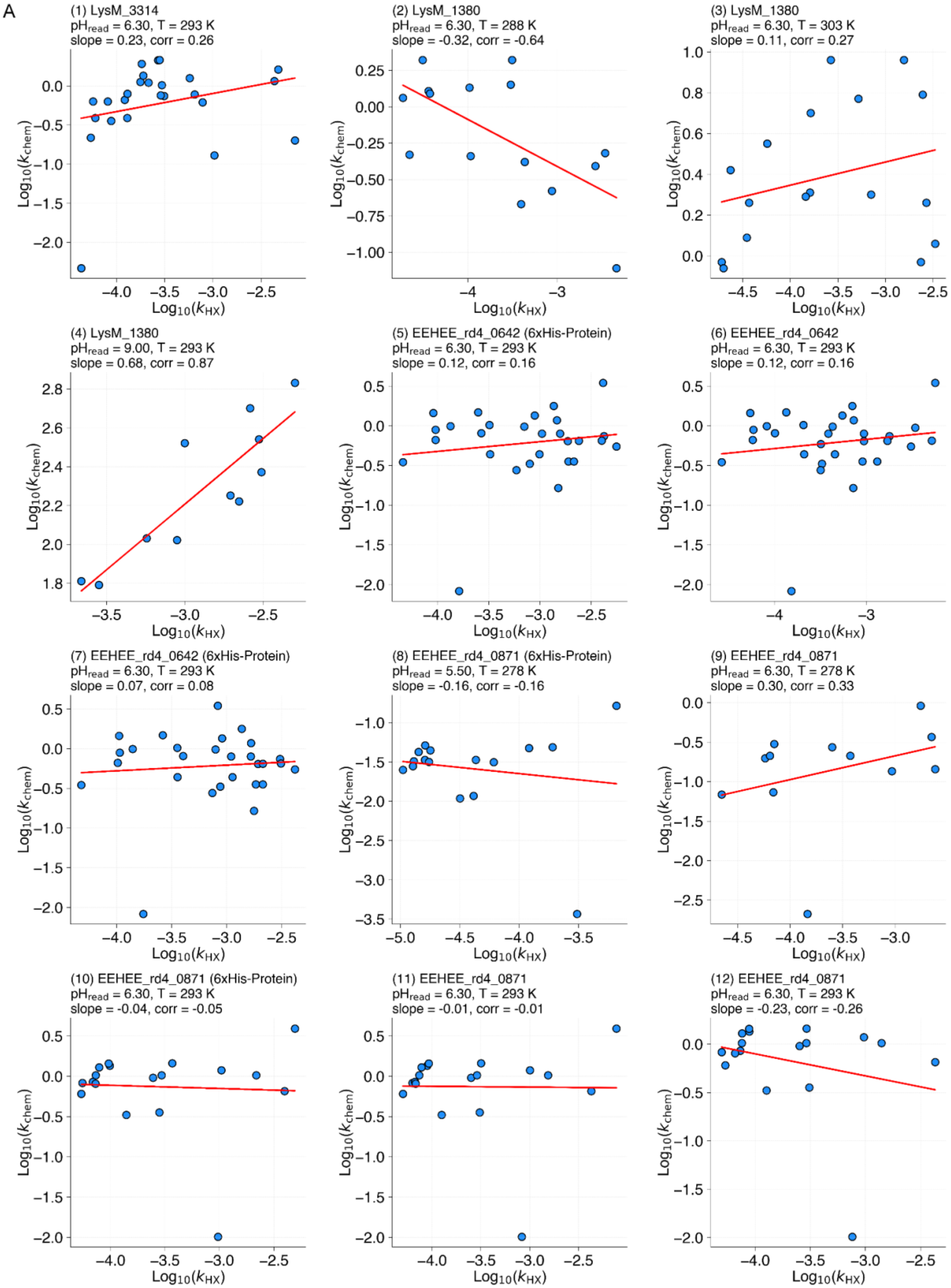
A
(1) LysM_3314
pHread = 6.30, T = 293 K
slope = 0.23, corr = 0.26
(2) LysM_1380
pHread = 6.30, T = 288 K
slope = -0.32, corr = -0.64
(3) LysM_1380
pHread = 6.30, T = 303 K
slope = 0.11, corr = 0.27
(4) LysM_1380
pHread = 9.00, T = 293 K
slope = 0.68, corr = 0.87
(5) EEHEE_rd4_0642 (6xHis-Protein)
pHread = 6.30, T = 293 K
slope = 0.12, corr = 0.16
(6) EEHEE_rd4_0642
pHread = 6.30, T = 293 K
slope = 0.12, corr = 0.16
(7) EEHEE_rd4_0642 (6xHis-Protein)
pHread = 6.30, T = 293 K
slope = 0.07, corr = 0.08
(8) EEHEE_rd4_0871 (6xHis-Protein)
pHread = 5.50, T = 278 K
slope = -0.16, corr = -0.16
(9) EEHEE_rd4_0871
pHread = 6.30, T = 278 K
slope = 0.30, corr = 0.33
(10) EEHEE_rd4_0871 (6xHis-Protein)
pHread = 6.30, T = 293 K
slope = -0.04, corr = -0.05
(11) EEHEE_rd4_0871
pHread = 6.30, T = 293 K
slope = -0.01, corr = -0.01
(12) EEHEE_rd4_0871
pHread = 6.30, T = 293 K
slope = -0.23, corr = -0.26
Log10(kchem)
Log10(kHX)

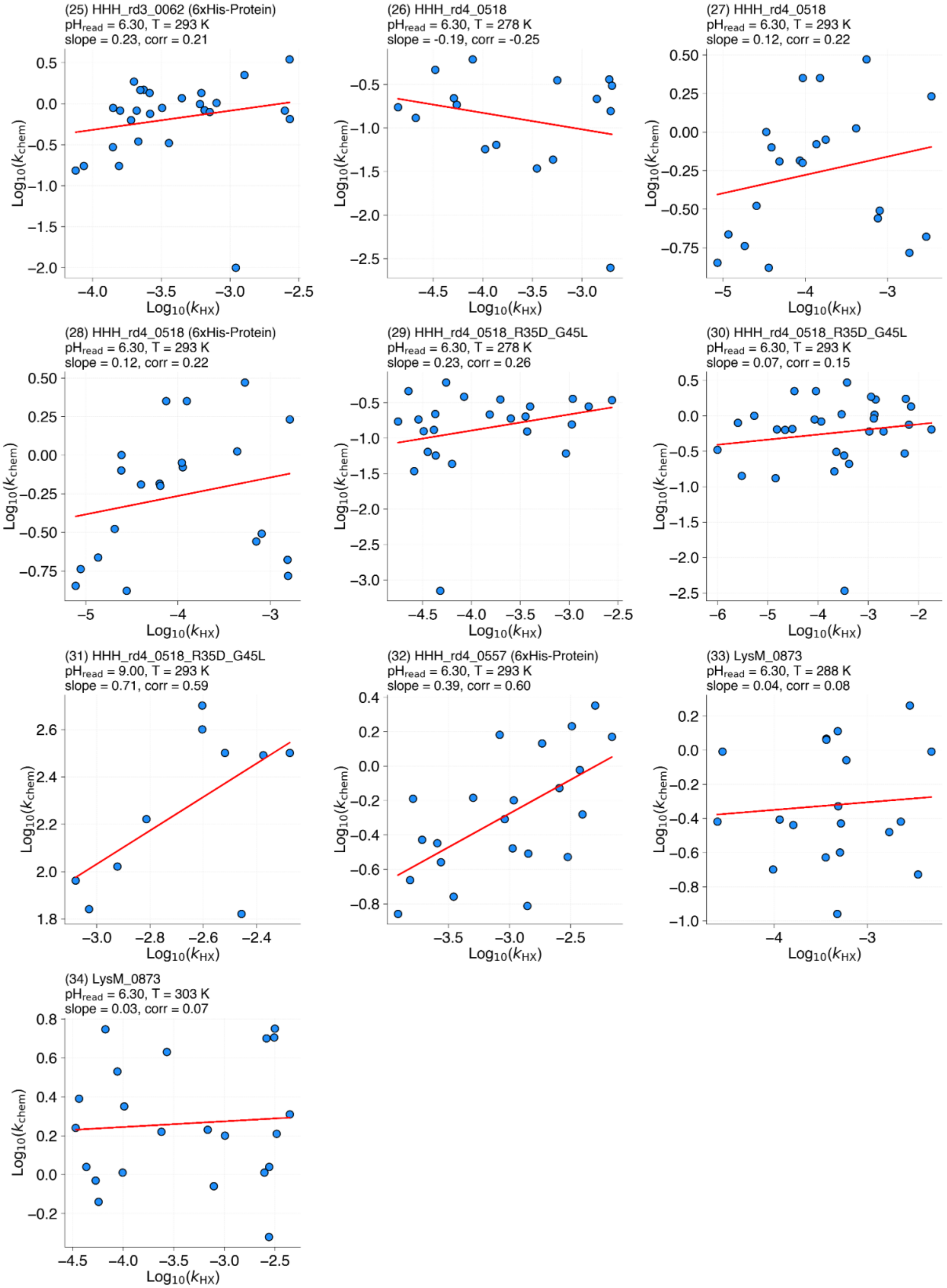

(25) HHH_rd3_0062 (6xHis-Protein)
pH_{read} = 6.30, T = 293 K
slope = 0.23, corr = 0.21
(26) HHH_rd4_0518
pH_{read} = 6.30, T = 278 K
slope = -0.19, corr = -0.25
(27) HHH_rd4_0518
pH_{read} = 6.30, T = 293 K
slope = 0.12, corr = 0.22
(28) HHH_rd4_0518 (6xHis-Protein)
pH_{read} = 6.30, T = 293 K
slope = 0.12, corr = 0.22
(29) HHH_rd4_0518_R35D_G45L
pH_{read} = 6.30, T = 278 K
slope = 0.23, corr = 0.26
(30) HHH_rd4_0518_R35D_G45L
pH_{read} = 6.30, T = 293 K
slope = 0.07, corr = 0.15
(31) HHH_rd4_0518_R35D_G45L
pH_{read} = 9.00, T = 293 K
slope = 0.71, corr = 0.59
(32) HHH_rd4_0557 (6xHis-Protein)
pH_{read} = 6.30, T = 293 K
slope = 0.39, corr = 0.60
(33) LysM_0873
pH_{read} = 6.30, T = 288 K
slope = 0.04, corr = 0.08
(34) LysM_0873
pH_{read} = 6.30, T = 303 K
slope = 0.03, corr = 0.07
$Log_{10}(k_{chem})$
$Log_{10}(k_{HX})$

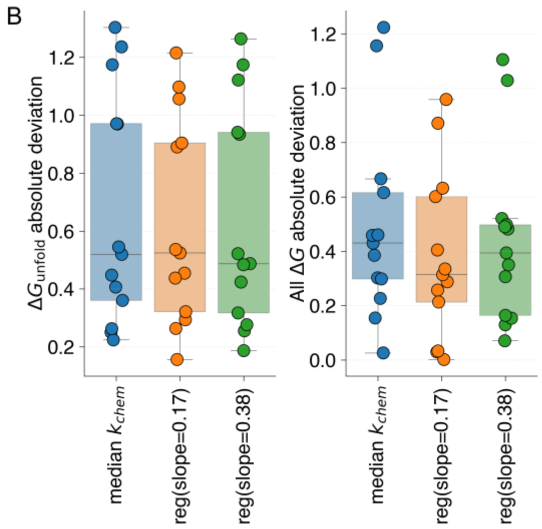

Figure S2: Estimation of intrinsic exchange rates and residue opening free energies (ΔG_{open}). (A) Correlation plots comparing the logarithm of experimentally inferred exchange rates (log k_{HX}) with intrinsic exchange rates (log k_{chem}) derived from residue-resolved HDX NMR for 11 proteins. Each panel shows the relationship for one protein and one condition (temperature, pH), demonstrating that slower k_{HX} values tend to be associated with slower k_{chem}. This trend is captured by fitting a linear model to z-scored log k_{HX} and log k_{chem} values, which forms the basis for our regression adjustment. (B) Boxplots comparing the absolute deviation in global stability (left) and across all measurable ΔG_{open} values (right) when estimating k_{chem} by (i) simply using the protein's median k_{chem}, (ii) using a regression model derived from a reduced dataset (slope = 0.17), and (iii) using a regression model based on the complete dataset (slope = 0.38). The regression-based adjustments yield ΔG_{open} estimates that more accurately recapitulate those obtained from HDX NMR, with the model using slope = 0.38 providing superior performance. In the box-plots, the center line indicates the median. The box limits represent the 25th and 75th percentiles (lower and upper quartiles). The whiskers extend to 1.5x the interquartile range beyond the quartiles. All data points are shown.

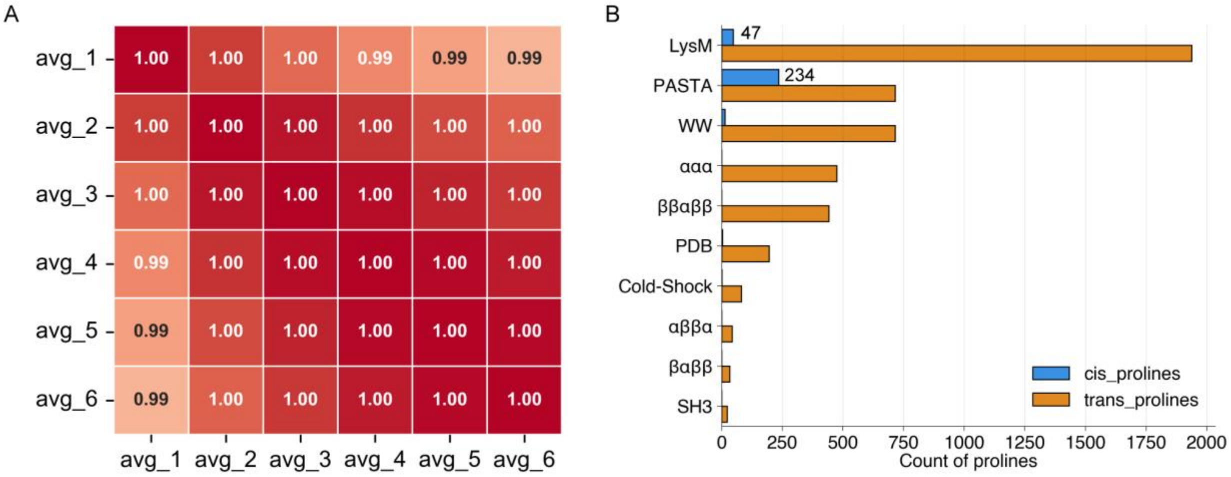

Figure S3: Robustness of ΔG_{unfold} estimation via averaging the most stable residues. (A) The correlation matrix displays pairwise Pearson correlation coefficients for stability values obtained by averaging the top 1 to 6 most stable residues (labeled avg_1 to avg_6). All pairwise correlations are ≥0.99, indicating that the stability metric is virtually invariant to the number of residues included in the average. (B) Counts of cis and trans prolines across protein families included in **Table S1 : Dataset_3**. While trans prolines are abundant across all families, cis prolines are predominantly found in PASTA domains, with few occurrences in LysM and WW domains. These data indicate that potential effects of cis prolines on ΔG_{unfold} estimations are largely confined to a subset of PASTA sequences.

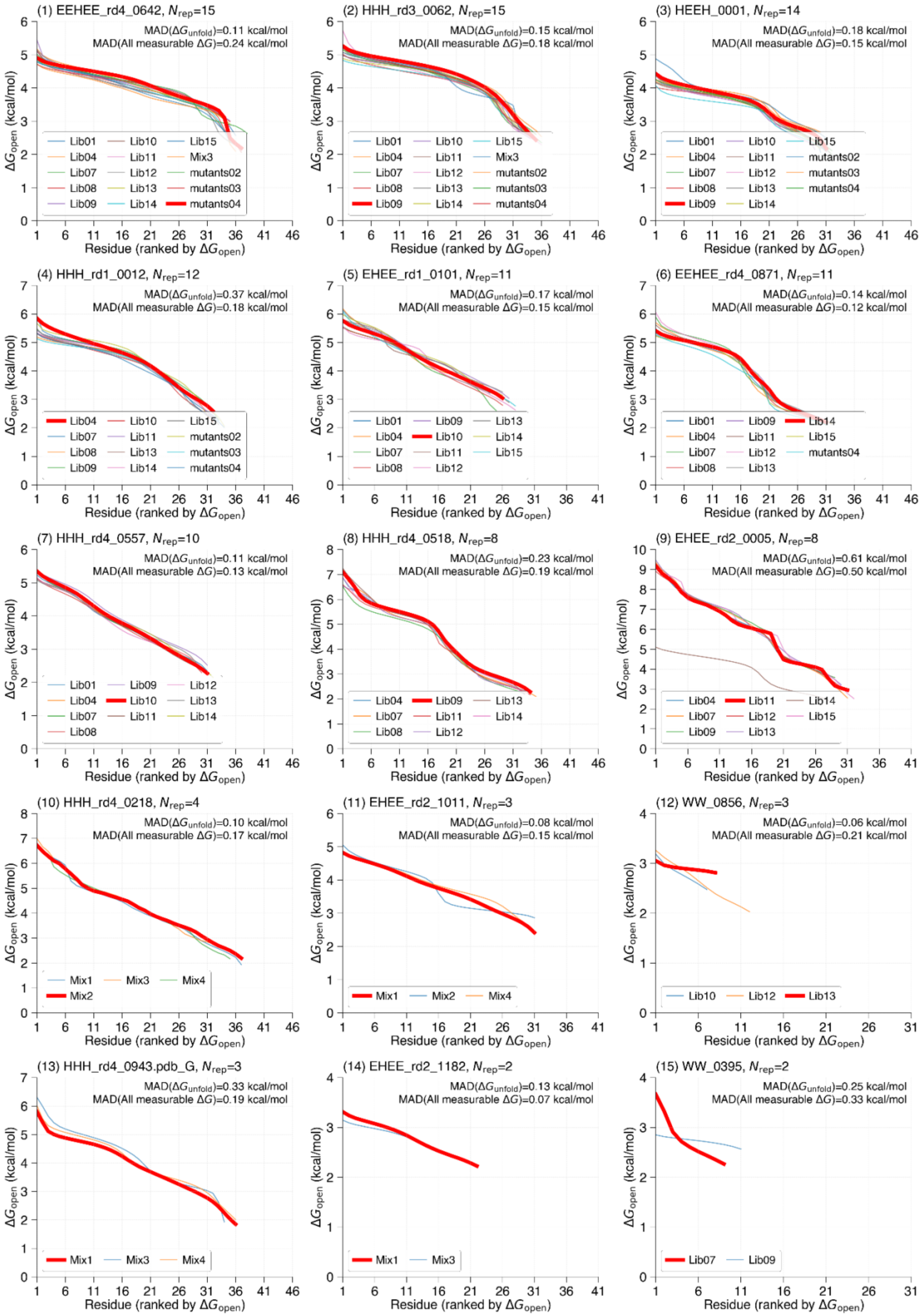
ΔG_open (kcal/mol)
Residue (ranked by ΔG_open)
(1) EEHEE_rd4_0642, N_rep=15
MAD(ΔG_unfold)=0.11 kcal/mol
MAD(All measurable ΔG)=0.24 kcal/mol
Lib01 Lib04 Lib07 Lib08 Lib09 Lib10 Lib11 Lib12 Lib13 Lib14 Lib15 Mix3 mutants02 mutants03 mutants04
(2) HHH_rd3_0062, N_rep=15
MAD(ΔG_unfold)=0.15 kcal/mol
MAD(All measurable ΔG)=0.18 kcal/mol
Lib01 Lib04 Lib07 Lib08 Lib09 Lib10 Lib11 Lib12 Lib13 Lib14 Lib15 Mix3 mutants02 mutants03 mutants04
(3) HEEH_0001, N_rep=14
MAD(ΔG_unfold)=0.18 kcal/mol
MAD(All measurable ΔG)=0.15 kcal/mol
Lib01 Lib04 Lib07 Lib08 Lib09 Lib10 Lib11 Lib12 Lib13 Lib14 Lib15 mutants02 mutants03 mutants04
(4) HHH_rd1_0012, N_rep=12
MAD(ΔG_unfold)=0.37 kcal/mol
MAD(All measurable ΔG)=0.18 kcal/mol
Lib04 Lib07 Lib08 Lib09 Lib10 Lib11 Lib13 Lib14 Lib15 mutants02 mutants03 mutants04
(5) EHEE_rd1_0101, N_rep=11
MAD(ΔG_unfold)=0.17 kcal/mol
MAD(All measurable ΔG)=0.15 kcal/mol
Lib01 Lib04 Lib07 Lib08 Lib09 Lib10 Lib11 Lib12 Lib13 Lib14 Lib15
(6) EEHEE_rd4_0871, N_rep=11
MAD(ΔG_unfold)=0.14 kcal/mol
MAD(All measurable ΔG)=0.12 kcal/mol
Lib01 Lib04 Lib07 Lib08 Lib09 Lib11 Lib12 Lib13 Lib14 Lib15 mutants04
(7) HHH_rd4_0557, N_rep=10
MAD(ΔG_unfold)=0.11 kcal/mol
MAD(All measurable ΔG)=0.13 kcal/mol
Lib01 Lib04 Lib07 Lib08 Lib09 Lib10 Lib11 Lib12 Lib13 Lib14
(8) HHH_rd4_0518, N_rep=8
MAD(ΔG_unfold)=0.23 kcal/mol
MAD(All measurable ΔG)=0.19 kcal/mol
Lib04 Lib07 Lib08 Lib09 Lib11 Lib12 Lib13 Lib14
(9) EHEE_rd2_0005, N_rep=8
MAD(ΔG_unfold)=0.61 kcal/mol
MAD(All measurable ΔG)=0.50 kcal/mol
Lib04 Lib07 Lib09 Lib11 Lib12 Lib13 Lib14 Lib15
(10) HHH_rd4_0218, N_rep=4
MAD(ΔG_unfold)=0.10 kcal/mol
MAD(All measurable ΔG)=0.17 kcal/mol
Mix1 Mix2 Mix3 Mix4
(11) EHEE_rd2_1011, N_rep=3
MAD(ΔG_unfold)=0.08 kcal/mol
MAD(All measurable ΔG)=0.15 kcal/mol
Mix1 Mix2 Mix4
(12) WW_0856, N_rep=3
MAD(ΔG_unfold)=0.06 kcal/mol
MAD(All measurable ΔG)=0.21 kcal/mol
Lib10 Lib12 Lib13
(13) HHH_rd4_0943.pdb_G, N_rep=3
MAD(ΔG_unfold)=0.33 kcal/mol
MAD(All measurable ΔG)=0.19 kcal/mol
Mix1 Mix3 Mix4
(14) EHEE_rd2_1182, N_rep=2
MAD(ΔG_unfold)=0.13 kcal/mol
MAD(All measurable ΔG)=0.07 kcal/mol
Mix1 Mix3
(15) WW_0395, N_rep=2
MAD(ΔG_unfold)=0.25 kcal/mol
MAD(All measurable ΔG)=0.33 kcal/mol
Lib07 Lib09

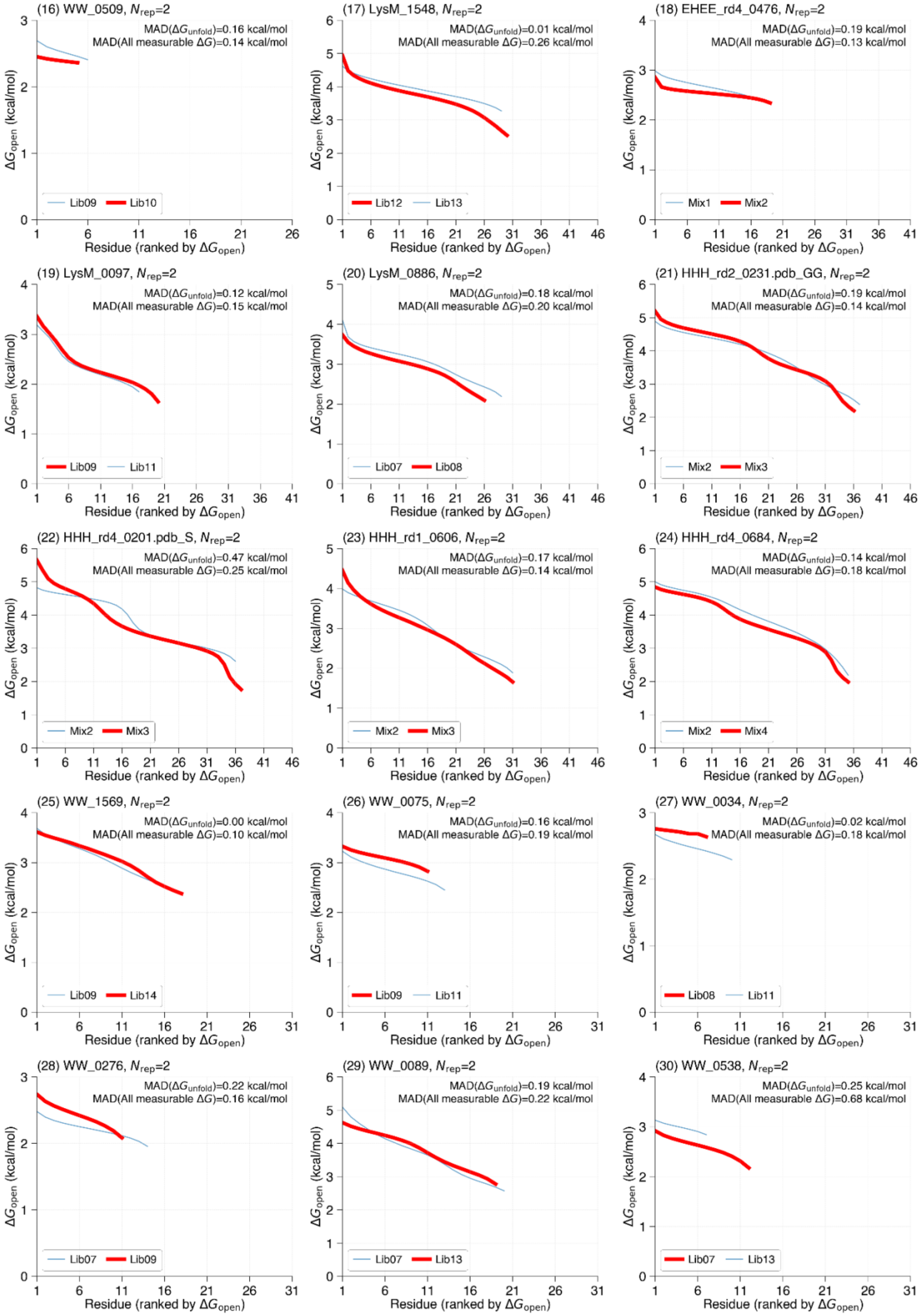

(16) WW_0509, N_{rep}=2
MAD(ΔG_{unfold})=0.16 kcal/mol
MAD(All measurable ΔG)=0.14 kcal/mol
Lib09 Lib10
(17) LysM_1548, N_{rep}=2
MAD(ΔG_{unfold})=0.01 kcal/mol
MAD(All measurable ΔG)=0.26 kcal/mol
Lib12 Lib13
(18) EHEE_rd4_0476, N_{rep}=2
MAD(ΔG_{unfold})=0.19 kcal/mol
MAD(All measurable ΔG)=0.13 kcal/mol
Mix1 Mix2
(19) LysM_0097, N_{rep}=2
MAD(ΔG_{unfold})=0.12 kcal/mol
MAD(All measurable ΔG)=0.15 kcal/mol
Lib09 Lib11
(20) LysM_0886, N_{rep}=2
MAD(ΔG_{unfold})=0.18 kcal/mol
MAD(All measurable ΔG)=0.20 kcal/mol
Lib07 Lib08
(21) HHH_rd2_0231.pdb_GG, N_{rep}=2
MAD(ΔG_{unfold})=0.19 kcal/mol
MAD(All measurable ΔG)=0.14 kcal/mol
Mix2 Mix3
(22) HHH_rd4_0201.pdb_S, N_{rep}=2
MAD(ΔG_{unfold})=0.47 kcal/mol
MAD(All measurable ΔG)=0.25 kcal/mol
Mix2 Mix3
(23) HHH_rd1_0606, N_{rep}=2
MAD(ΔG_{unfold})=0.17 kcal/mol
MAD(All measurable ΔG)=0.14 kcal/mol
Mix2 Mix3
(24) HHH_rd4_0684, N_{rep}=2
MAD(ΔG_{unfold})=0.14 kcal/mol
MAD(All measurable ΔG)=0.18 kcal/mol
Mix2 Mix4
(25) WW_1569, N_{rep}=2
MAD(ΔG_{unfold})=0.00 kcal/mol
MAD(All measurable ΔG)=0.10 kcal/mol
Lib09 Lib14
(26) WW_0075, N_{rep}=2
MAD(ΔG_{unfold})=0.16 kcal/mol
MAD(All measurable ΔG)=0.19 kcal/mol
Lib09 Lib11
(27) WW_0034, N_{rep}=2
MAD(ΔG_{unfold})=0.02 kcal/mol
MAD(All measurable ΔG)=0.18 kcal/mol
Lib08 Lib11
(28) WW_0276, N_{rep}=2
MAD(ΔG_{unfold})=0.22 kcal/mol
MAD(All measurable ΔG)=0.16 kcal/mol
Lib07 Lib09
(29) WW_0089, N_{rep}=2
MAD(ΔG_{unfold})=0.19 kcal/mol
MAD(All measurable ΔG)=0.22 kcal/mol
Lib07 Lib13
(30) WW_0538, N_{rep}=2
MAD(ΔG_{unfold})=0.25 kcal/mol
MAD(All measurable ΔG)=0.68 kcal/mol
Lib07 Lib13
ΔG_{open} (kcal/mol)
Residue (ranked by ΔG_{open})

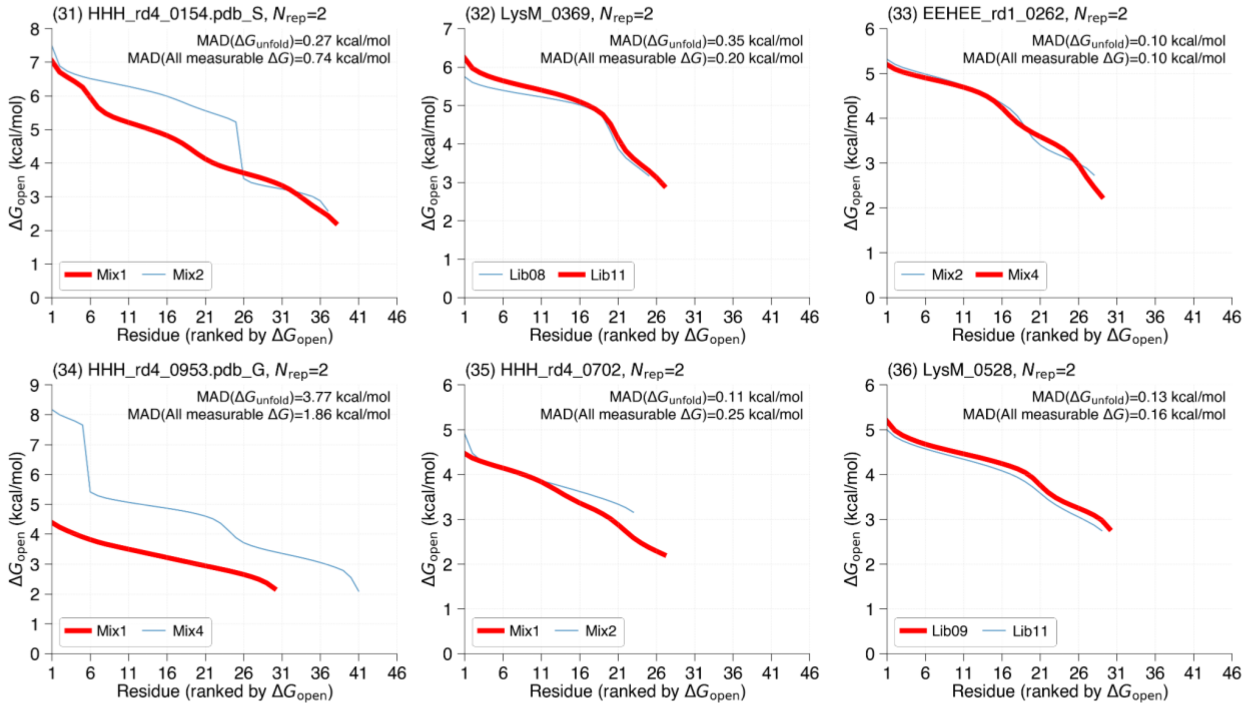

Figure S4: Reproducibility of mHDX-MS measurements across independent libraries. Each panel shows the reproducibility for one protein sequence that was measured in at least two independent mHDX-MS libraries (36 sequences in total). Panels are arranged in order of decreasing number of libraries in which the protein was detected. Within each panel, replicate measurements are displayed and the representative measurement - defined as the replicate with the lowest path optimizer score - is highlighted in red. The number of replicates (N_{rep}) is indicated, and the mean absolute deviations for ΔG_{unfold} and for all measurable ΔG_{open} values are computed relative to the representative value.

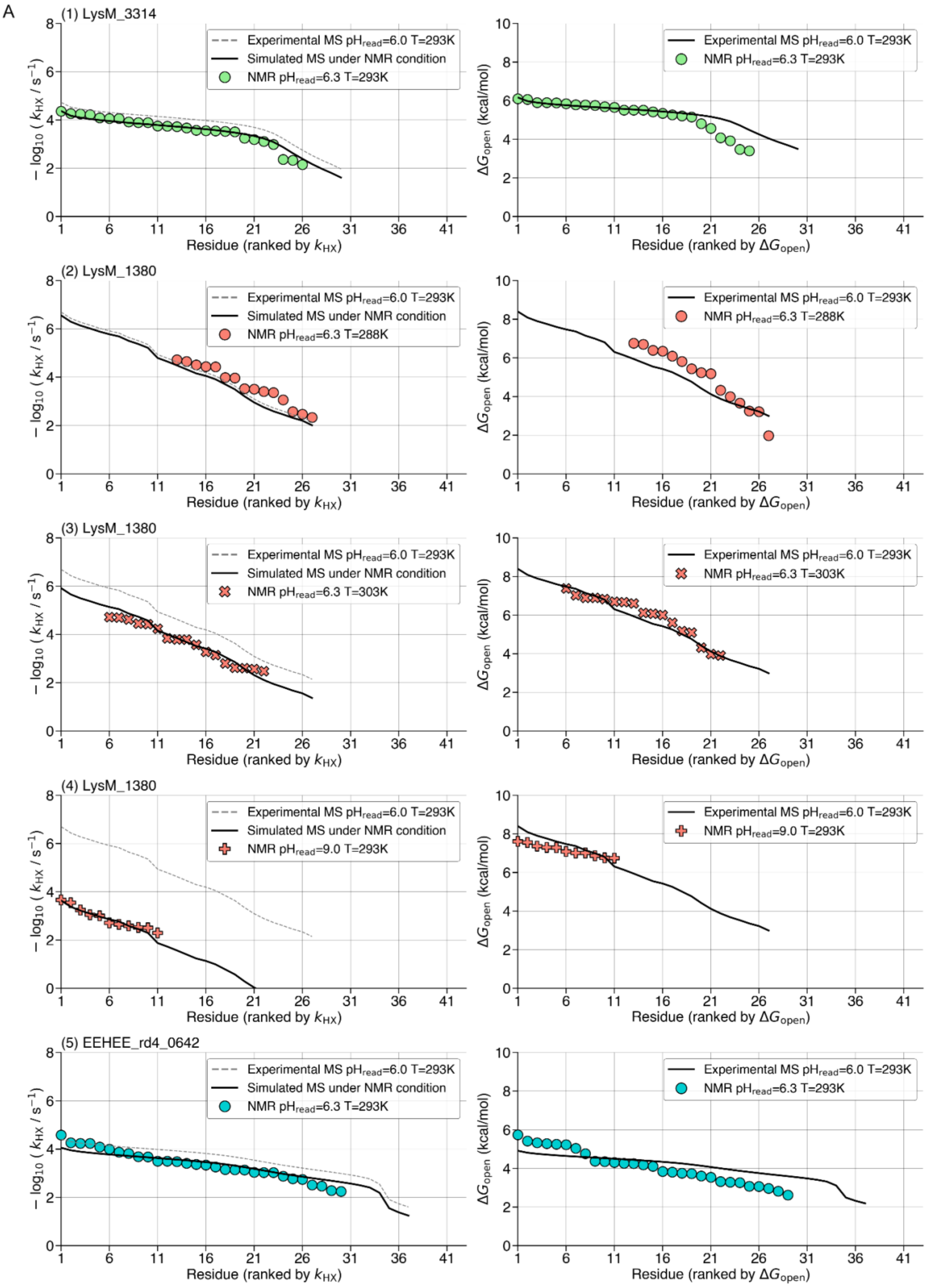

A
(1) LysM_3314
Experimental MS pH$_{read}$=6.0 T=293K
Simulated MS under NMR condition
NMR pH$_{read}$=6.3 T=293K
(2) LysM_1380
NMR pH$_{read}$=6.3 T=288K
(3) LysM_1380
NMR pH$_{read}$=6.3 T=303K
(4) LysM_1380
NMR pH$_{read}$=9.0 T=293K
(5) EEHEE_rd4_0642
$-\log_{10}(k_{HX} / s^{-1})$
ΔG_{open} (kcal/mol)
Residue (ranked by k_{HX})
Residue (ranked by ΔG_{open})

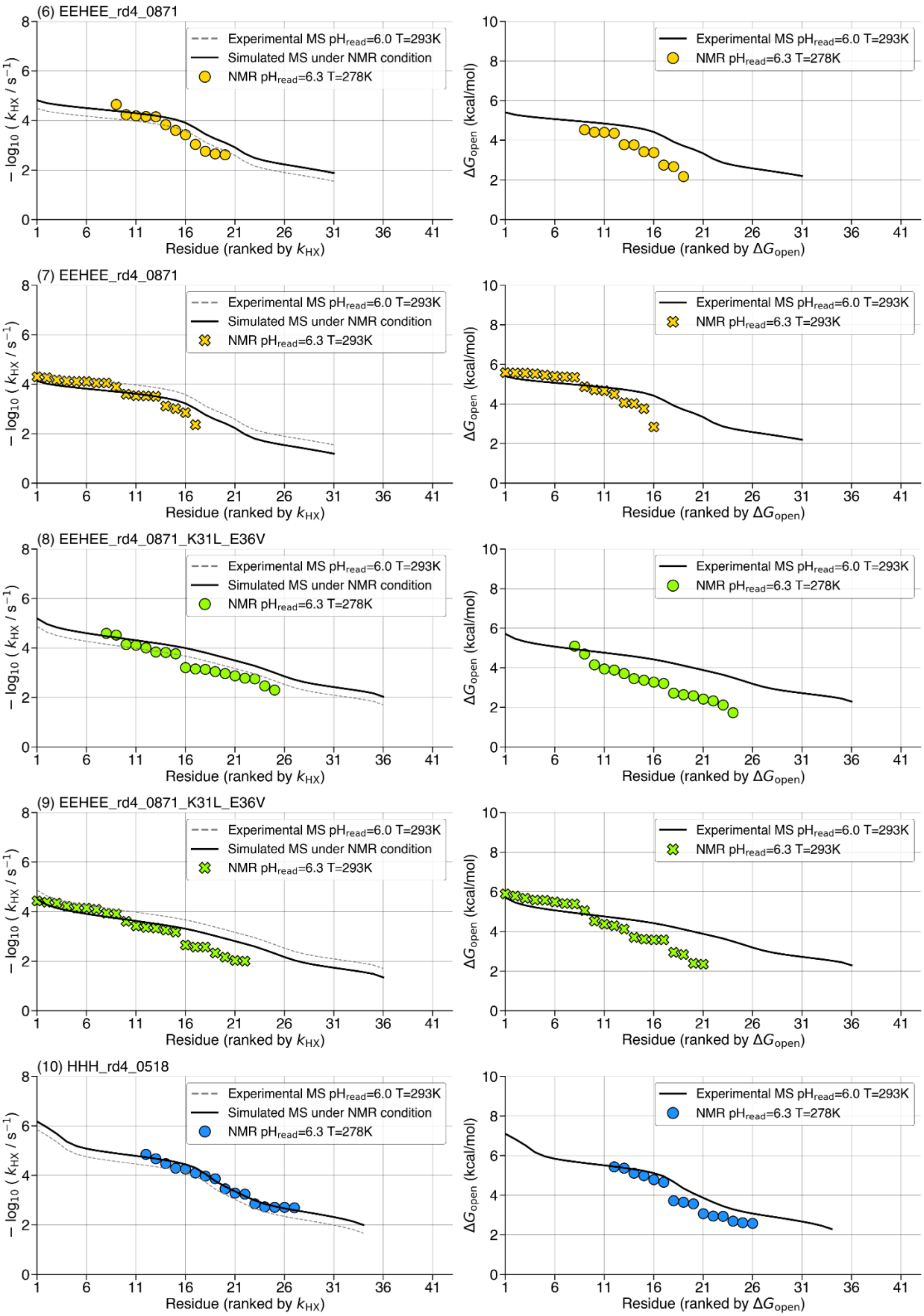

(6) EEHEE_rd4_0871
Experimental MS pHread=6.0 T=293K
Simulated MS under NMR condition
NMR pHread=6.3 T=278K
− log10 (kHX / s−1)
Residue (ranked by kHX)
ΔGopen (kcal/mol)
Residue (ranked by ΔGopen)
(7) EEHEE_rd4_0871
NMR pHread=6.3 T=293K
(8) EEHEE_rd4_0871_K31L_E36V
(9) EEHEE_rd4_0871_K31L_E36V
(10) HHH_rd4_0518

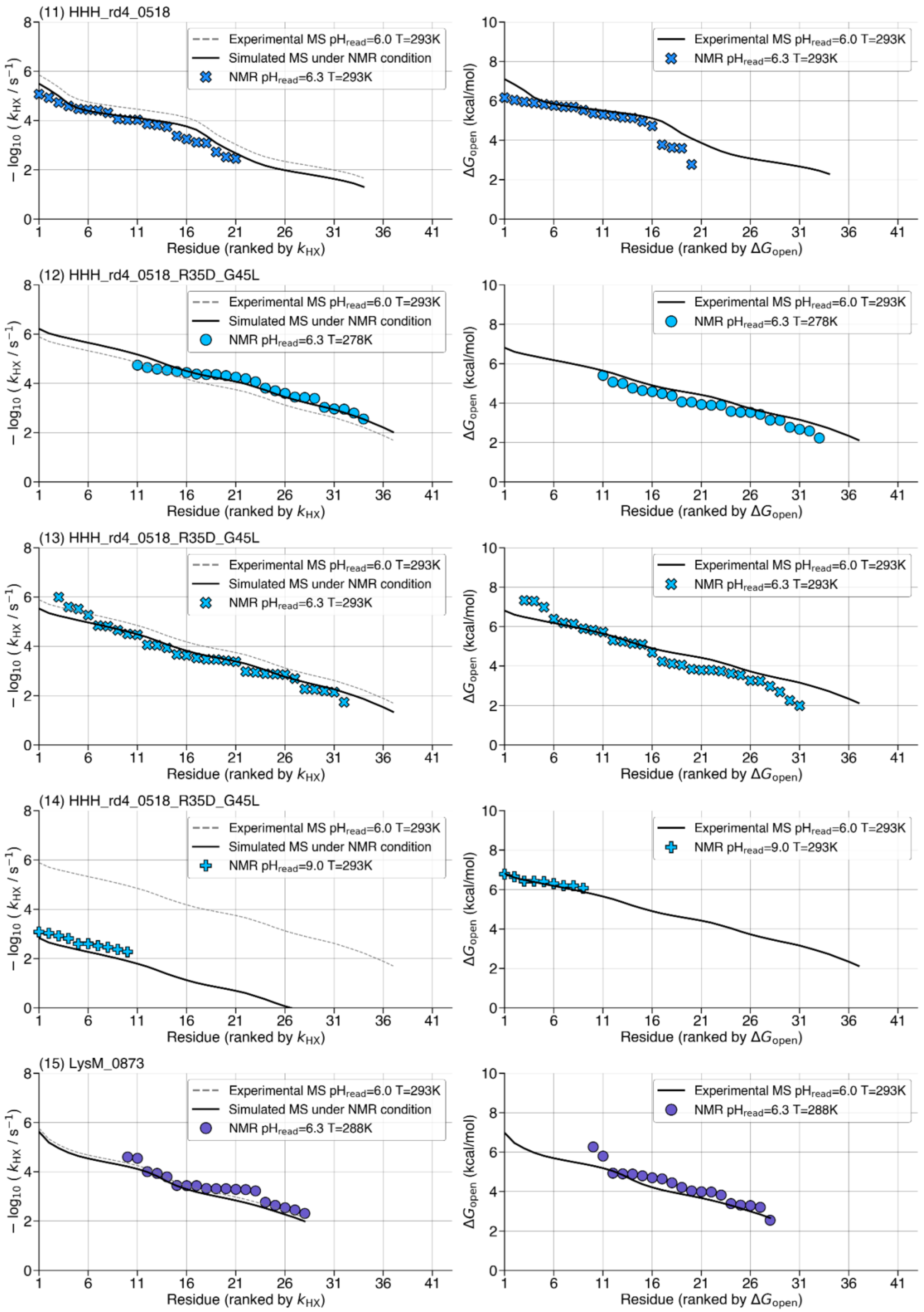

(11) HHH_rd4_0518
Experimental MS pH_{read}=6.0 T=293K
Simulated MS under NMR condition
NMR pH_{read}=6.3 T=293K
$-\log_{10}(k_{HX} / s^{-1})$
Residue (ranked by k_{HX})
Experimental MS pH_{read}=6.0 T=293K
NMR pH_{read}=6.3 T=293K
ΔG_{open} (kcal/mol)
Residue (ranked by ΔG_{open})
(12) HHH_rd4_0518_R35D_G45L
Experimental MS pH_{read}=6.0 T=293K
Simulated MS under NMR condition
NMR pH_{read}=6.3 T=278K
$-\log_{10}(k_{HX} / s^{-1})$
Residue (ranked by k_{HX})
Experimental MS pH_{read}=6.0 T=293K
NMR pH_{read}=6.3 T=278K
ΔG_{open} (kcal/mol)
Residue (ranked by ΔG_{open})
(13) HHH_rd4_0518_R35D_G45L
Experimental MS pH_{read}=6.0 T=293K
Simulated MS under NMR condition
NMR pH_{read}=6.3 T=293K
$-\log_{10}(k_{HX} / s^{-1})$
Residue (ranked by k_{HX})
Experimental MS pH_{read}=6.0 T=293K
NMR pH_{read}=6.3 T=293K
ΔG_{open} (kcal/mol)
Residue (ranked by ΔG_{open})
(14) HHH_rd4_0518_R35D_G45L
Experimental MS pH_{read}=6.0 T=293K
Simulated MS under NMR condition
NMR pH_{read}=9.0 T=293K
$-\log_{10}(k_{HX} / s^{-1})$
Residue (ranked by k_{HX})
Experimental MS pH_{read}=6.0 T=293K
NMR pH_{read}=9.0 T=293K
ΔG_{open} (kcal/mol)
Residue (ranked by ΔG_{open})
(15) LysM_0873
Experimental MS pH_{read}=6.0 T=293K
Simulated MS under NMR condition
NMR pH_{read}=6.3 T=288K
$-\log_{10}(k_{HX} / s^{-1})$
Residue (ranked by k_{HX})
Experimental MS pH_{read}=6.0 T=293K
NMR pH_{read}=6.3 T=288K
ΔG_{open} (kcal/mol)
Residue (ranked by ΔG_{open})

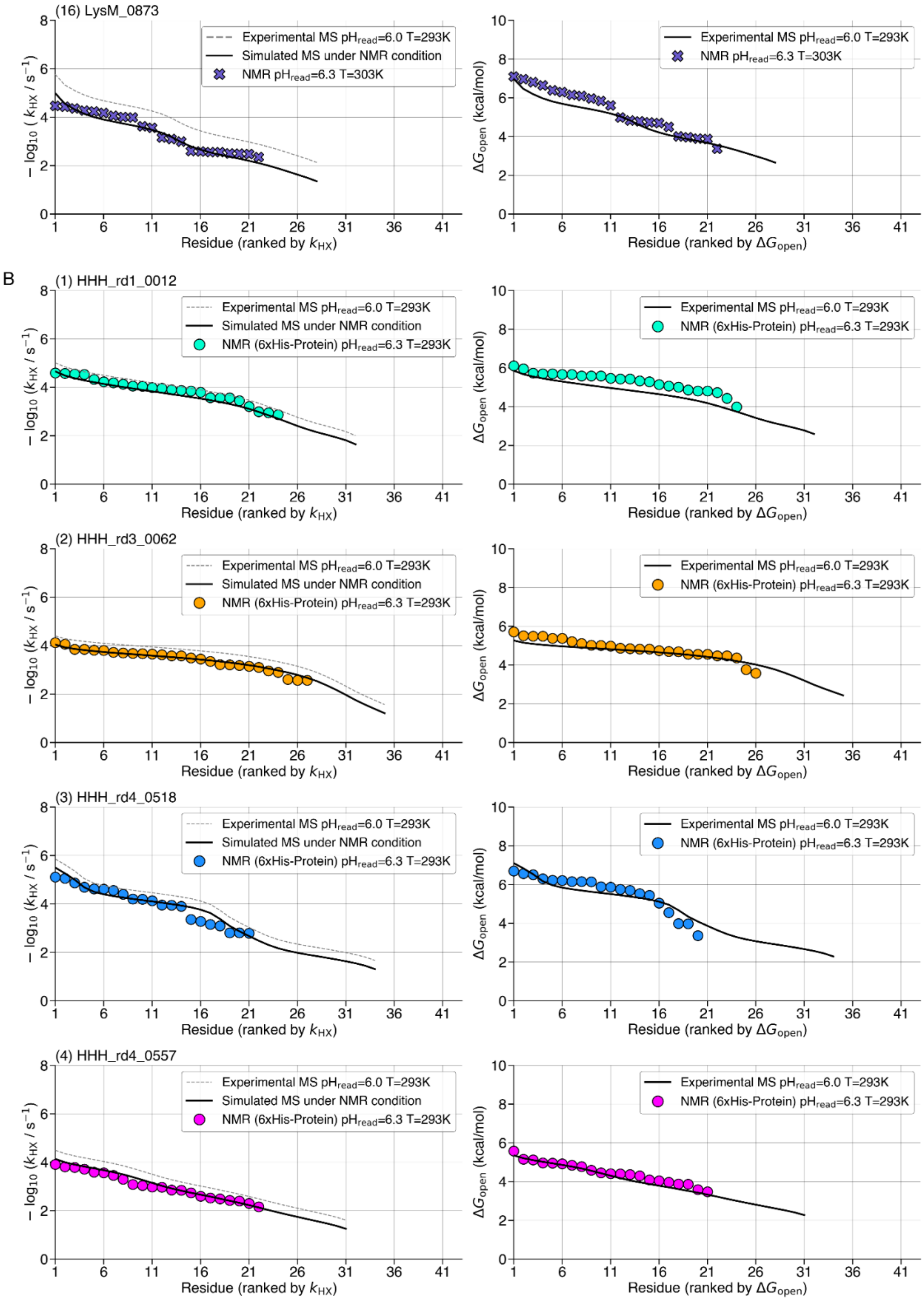
(16) LysM_0873
Experimental MS pH$_{read}$=6.0 T=293K
Simulated MS under NMR condition
NMR pH$_{read}$=6.3 T=303K
$-\log_{10}$ (k_{HX} / s^{-1})
Residue (ranked by k_{HX})
ΔG_{open} (kcal/mol)
Residue (ranked by ΔG_{open})
B
(1) HHH_rd1_0012
NMR (6xHis-Protein) pH$_{read}$=6.3 T=293K
(2) HHH_rd3_0062
(3) HHH_rd4_0518
(4) HHH_rd4_0557

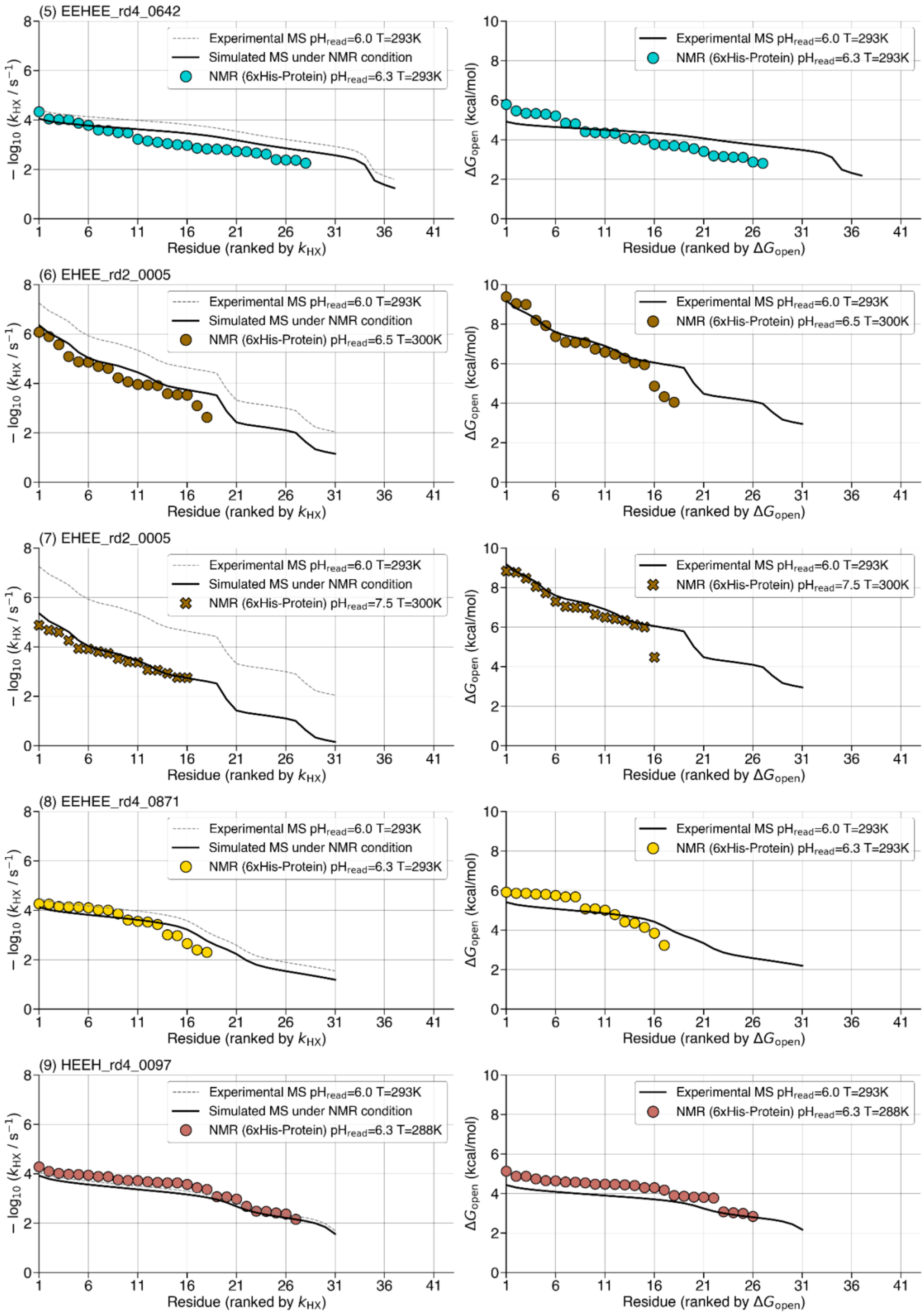
(5) EEHEE_rd4_0642
Experimental MS pHread=6.0 T=293K
Simulated MS under NMR condition
NMR (6xHis-Protein) pHread=6.3 T=293K
− log10 (kHX / s−1)
Residue (ranked by kHX)
ΔGopen (kcal/mol)
Residue (ranked by ΔGopen)
(6) EHEE_rd2_0005
Experimental MS pHread=6.0 T=293K
Simulated MS under NMR condition
NMR (6xHis-Protein) pHread=6.5 T=300K
(7) EHEE_rd2_0005
Experimental MS pHread=6.0 T=293K
Simulated MS under NMR condition
NMR (6xHis-Protein) pHread=7.5 T=300K
(8) EEHEE_rd4_0871
Experimental MS pHread=6.0 T=293K
Simulated MS under NMR condition
NMR (6xHis-Protein) pHread=6.3 T=293K
(9) HEEH_rd4_0097
Experimental MS pHread=6.0 T=293K
Simulated MS under NMR condition
NMR (6xHis-Protein) pHread=6.3 T=288K

Figure S5: Comparative analysis of HDX NMR and mHDX-MS measurements. Thirteen unique protein domains were assessed using both multiplexed hydrogen-deuterium exchange mass spectrometry (mHDX-MS) and hydrogen-deuterium exchange NMR (HDX NMR) under various experimental conditions. All proteins analyzed by mHDX-MS had their 6×His-SUMO tags cleaved prior to measurement. Two classes of comparison are shown: (A) eight protein domains evaluated using nearly identical constructs between mHDX-MS and NMR, where the NMR constructs include only one or two extra residues at the N-termini; and (B) eight protein domain constructs where the NMR construct contains a long N-terminal tag (MGSSHHHHHHSSGLVPRGS). Each row corresponds to a specific protein under a given experimental condition, with multiple rows per protein representing different HDX NMR conditions. The left panels display exchange rates (k_{HX}) from mHDX-MS (grey dashed lines) and HDX NMR (scatter), along with simulated mHDX-MS rates under replicated NMR conditions (black line), illustrating the agreement between the two methods. Simulated mHDX-MS rates were derived by modifying the measured rates according to the ratio between the median k_{chem} between the two conditions. The right panels show the corresponding opening energy distributions (ΔG_{open}) from both mHDX-MS (black lines) and HDX NMR (scatter). Exchange rates were measured at pH 6.0-6.3 (MES buffer), pH 7.5 (PBS buffer), and pH 9.0 (bicine buffer). When some residues exchanged too slowly to resolve by NMR, these residues are not shown. For example, in #3 LysM_1380 at pH 6.3 303K, the slowest five residues were too slow to determine k_{HX} over 24 hours, so no NMR rate is shown for residues rank 1 to rank 5.

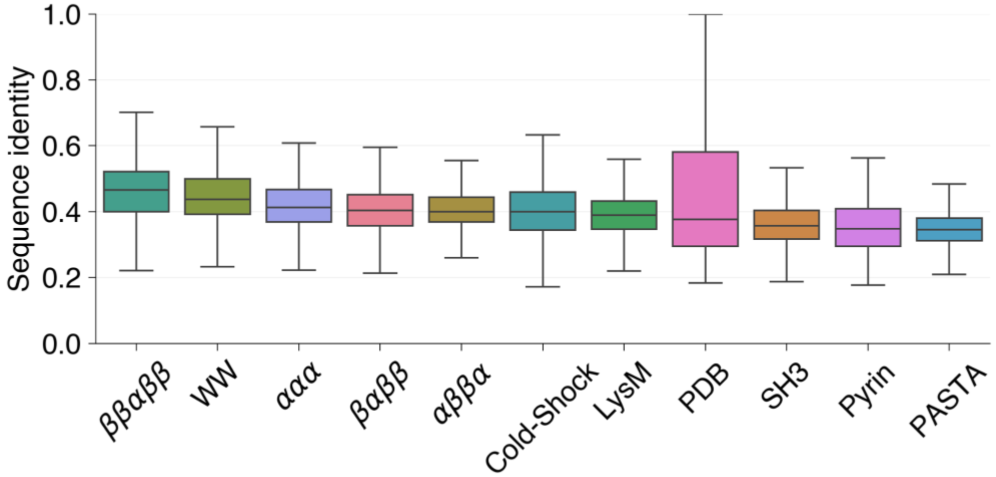

Figure S6: Distribution of pairwise sequence identity for different protein families. Sequences within each family were clustered using MMseqs2 (easy-cluster) with a dynamic threshold for minimum sequence identity (--min-seq-id ranging from 0.1 to 0.75), stopping when the largest cluster contained ≤10% of the total sequences. Representative sequences from the final clustering step were selected, and pairwise sequence identity was computed against all members within their respective clusters using MMseqs2's search function. Alignments were filtered to remove redundant sequence pairs, retaining only the highest-scoring alignment per unique sequence pair. The identity values were further corrected by weighting sequence identity by alignment length relative to the shortest sequence in the pair. Alignments between identical sequences (query == target) were excluded, and spurious short alignments were removed (E-value > 1e-3). The final distribution aggregates pairwise identity values across all clusters within each protein family. Outliers were hidden in the boxplot for clarity. In the box-plots, the center line indicates the median. The box limits represent the 25th and 75th percentiles (lower and upper quartiles). The whiskers extend to 1.5x the interquartile range beyond the quartiles. Outliers falling outside the whiskers are not displayed.

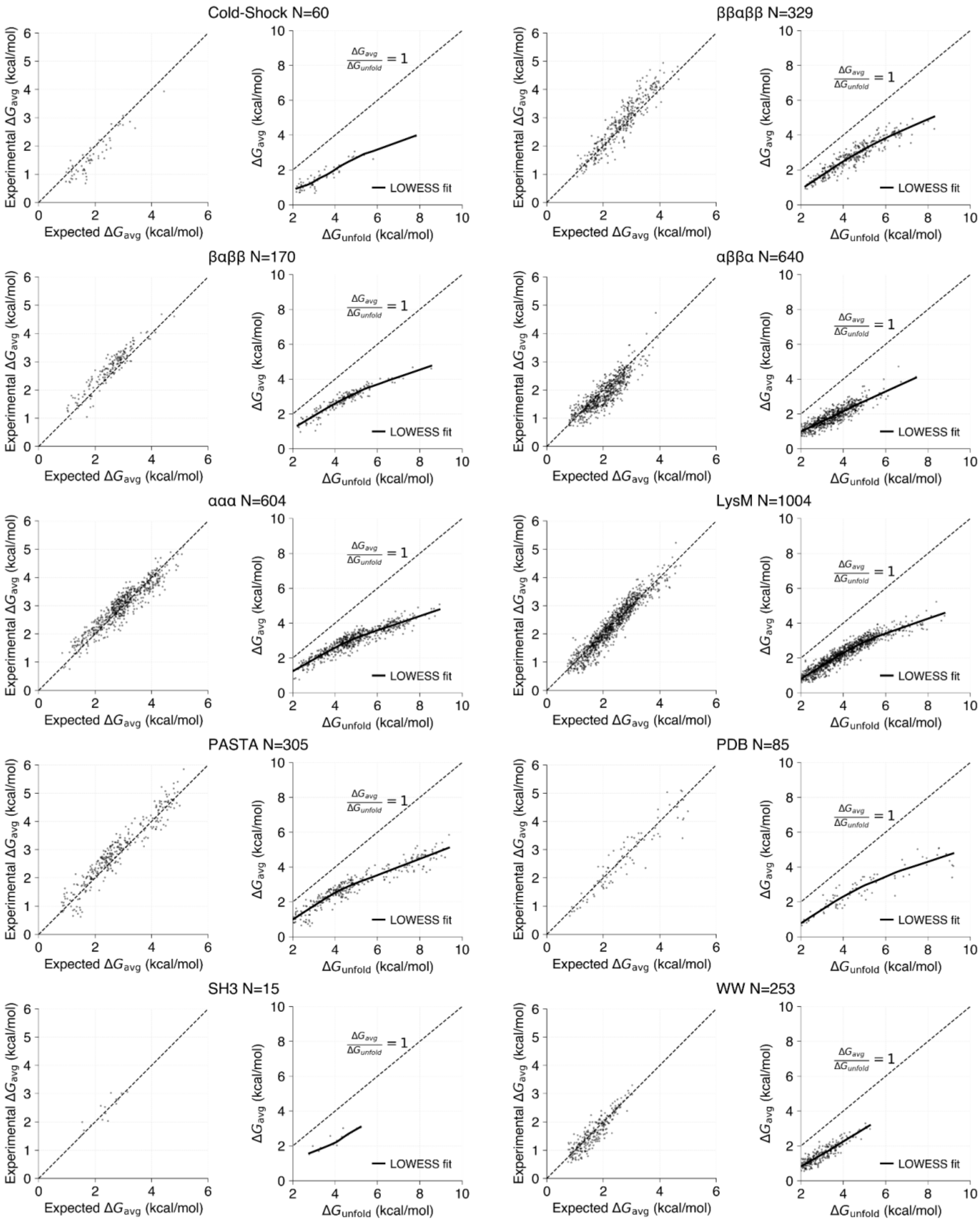

Figure S7: Empirical modeling of normalized cooperativity and family-normalized cooperativity. For each protein family, we fit an empirical model to predict the expected average opening free energy ($\Delta G_{avg,expected}$) as a function of global stability (ΔG_{unfold}), the fraction of backbone hydrogen bonds (fxn_hb), and net charge. The model is defined as $\Delta G_{avg,expected}$ = a ·

$(\Delta G_{unfold} - b)^c \cdot (\text{fxn\_hb})^d + e \cdot \text{netq}$, where the parameters a, b, c, d, and e are estimated from non-informative priors. Family-normalized cooperativity is then computed as the z-scored residual between the experimentally determined ΔG_{avg} and $\Delta G_{avg,expected}$, effectively decoupling cooperativity from stability. Additionally, for each protein family, we illustrate the sub-linear relationship between ΔG_{unfold} and ΔG_{avg}, with a diagonal dashed line indicating a linear dependency for reference.

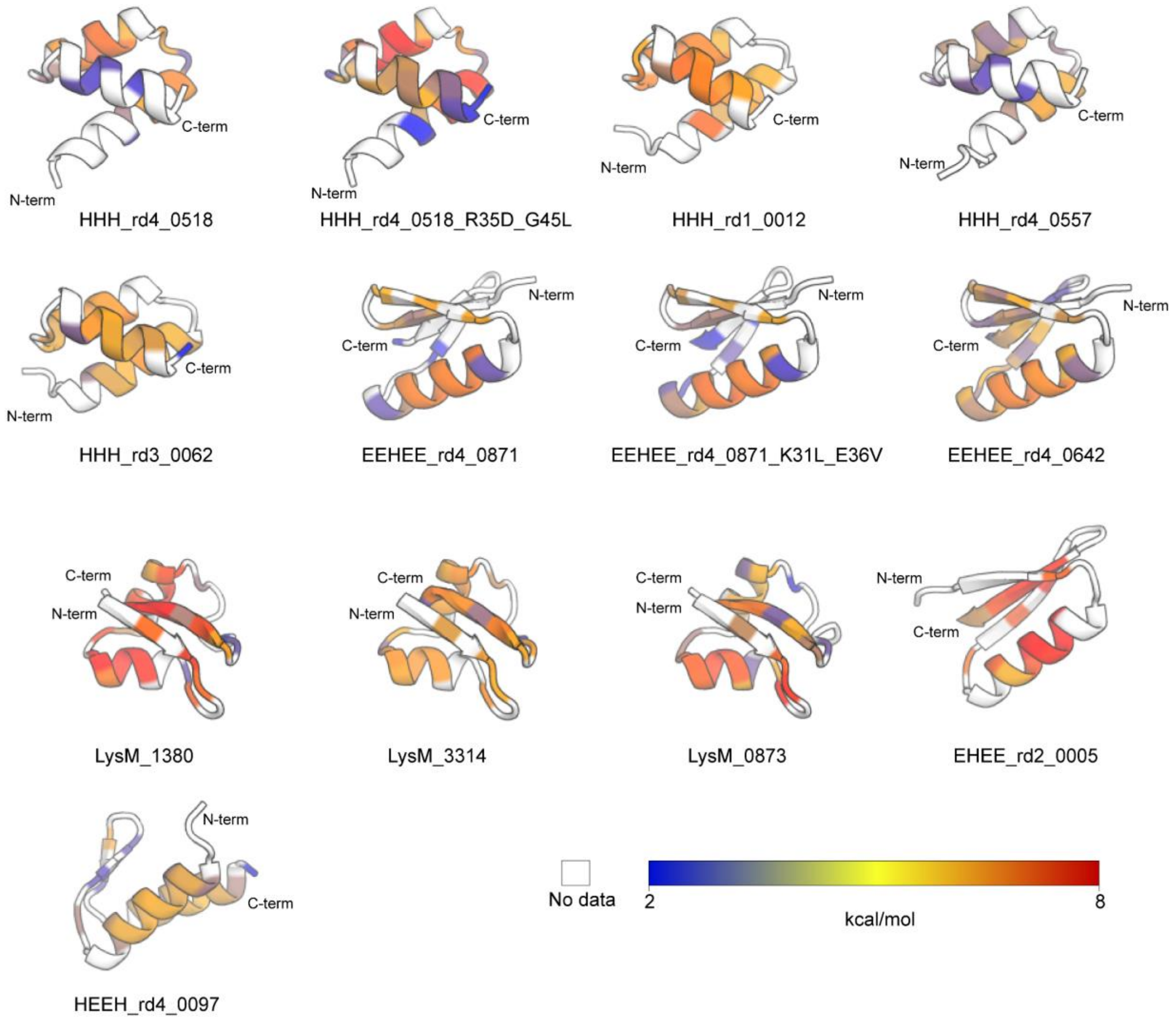

Figure S8: AlphaFold-derived structural models of all proteins characterized by HDX NMR. Each residue is colored according to its measured opening energy (ΔG_{open}), highlighting variations in local stability across the protein structure. Residues exchanging too rapidly to quantify are colored white.

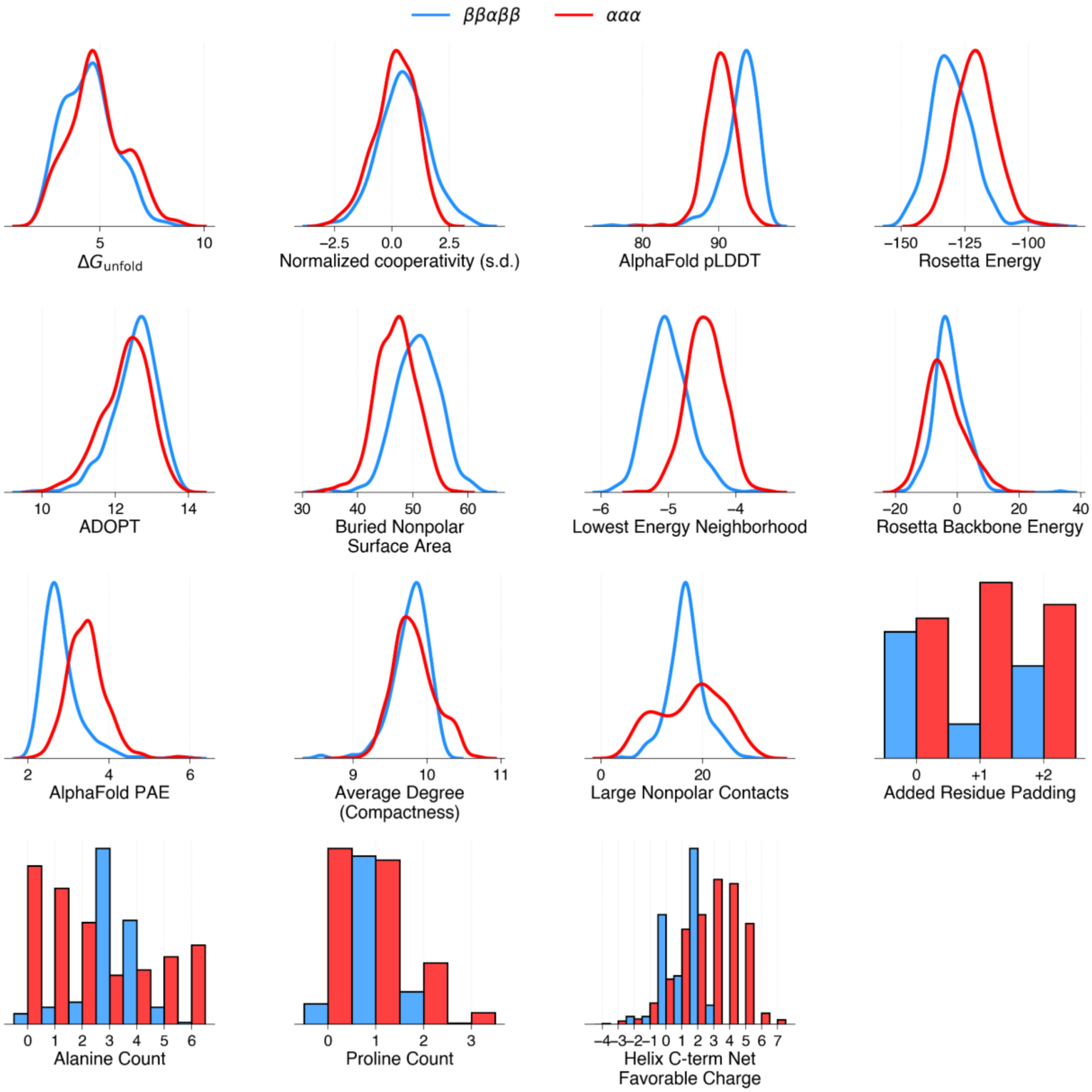

Figure S9: Feature distributions. Distributions for target variables and notable features highlighted in Figure 4 are shown for ααα (red) and ββαββ (blue) topologies. Kernel density plots are shown for continuous variables and histograms are shown for discrete variables.

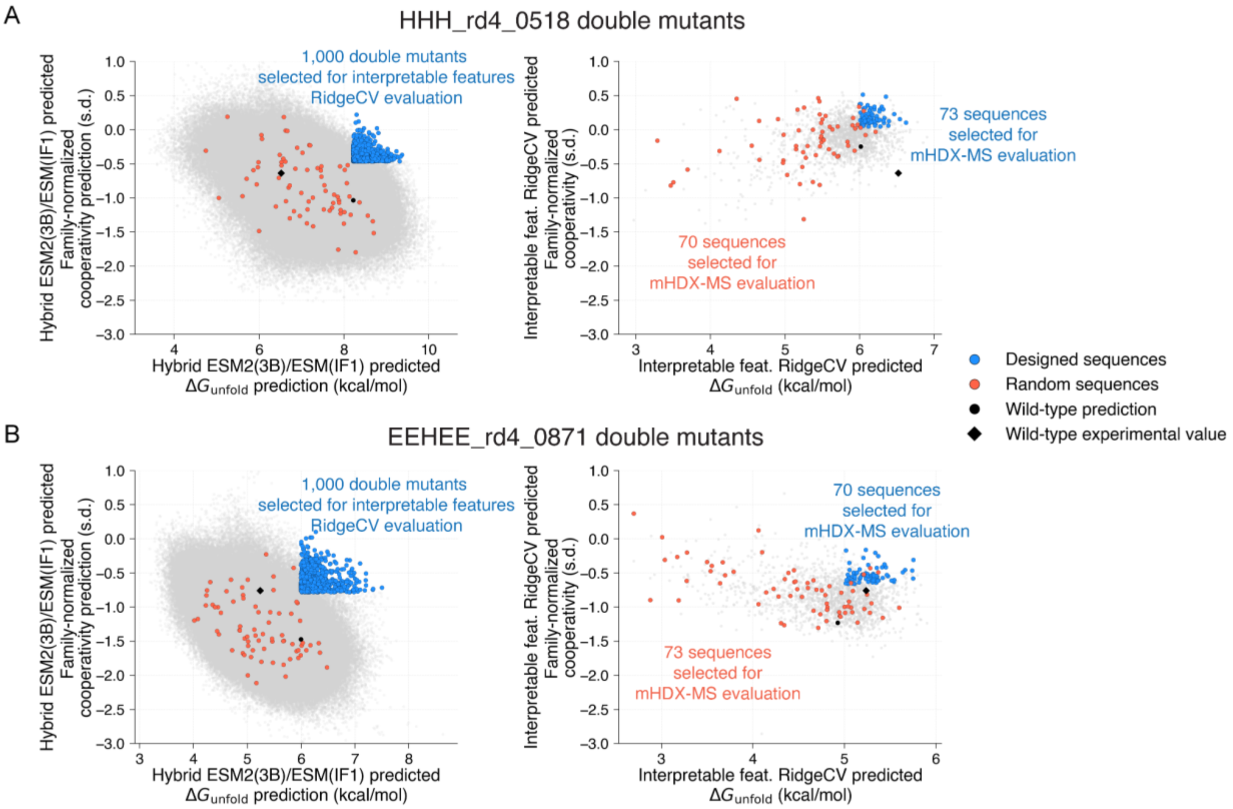

Figure S10: Selection of double mutants for experimental testing. (A) For HHH_rd4_0518, the best-performing first-generation protein language model was used to evaluate all possible double mutants (grey dots, left panel). The top 1,000 candidates were then selected (blue dots, right panel) and further evaluated using RidgeCV trained on interpretable features. Among these, 73 double mutants exhibiting both higher family-normalized cooperativity and ΔG_{unfold} compared to the wildtype (indicated by the black circle) were chosen for experimental testing; an additional 70 mutants were randomly selected (red dots, shown in both panels). (B) A similar workflow was applied to EEHEE_rd4_0871: all possible double mutants were initially evaluated (grey dots, left panel), and the top 1,000 candidates were filtered using the RidgeCV model trained on interpretable features (blue dots, left panel). From these, 73 double mutants (blue dots, right panel) with predicted properties exceeding those of the wildtype (black circle) were selected for testing, with another 70 mutants chosen at random (red dots, shown in both panels).

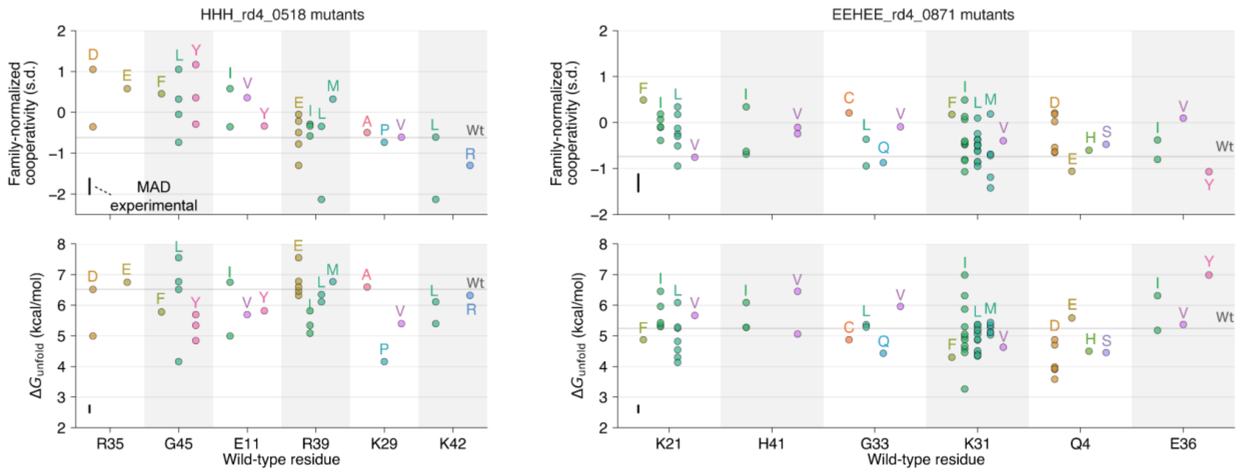

Figure S11. Impact of designed double mutants on protein ΔG_{unfold} and family-normalized cooperativity. (A) For HHH_rd4_0518, the top panel shows the changes in family-normalized cooperativity and the bottom panel shows the corresponding changes in ΔG_{unfold} for designed double mutants at the six most frequently mutated positions. Wild-type residues at these positions are ranked from highest to lowest average family-normalized cooperativity, providing a reference for assessing the impact of the mutations. Vertical scale bars show experimental mean absolute deviations (MAD) between replicates (of other proteins) measured in multiple libraries (**Fig. S4**). (B) As in A, for EEHEE_rd4_0871.

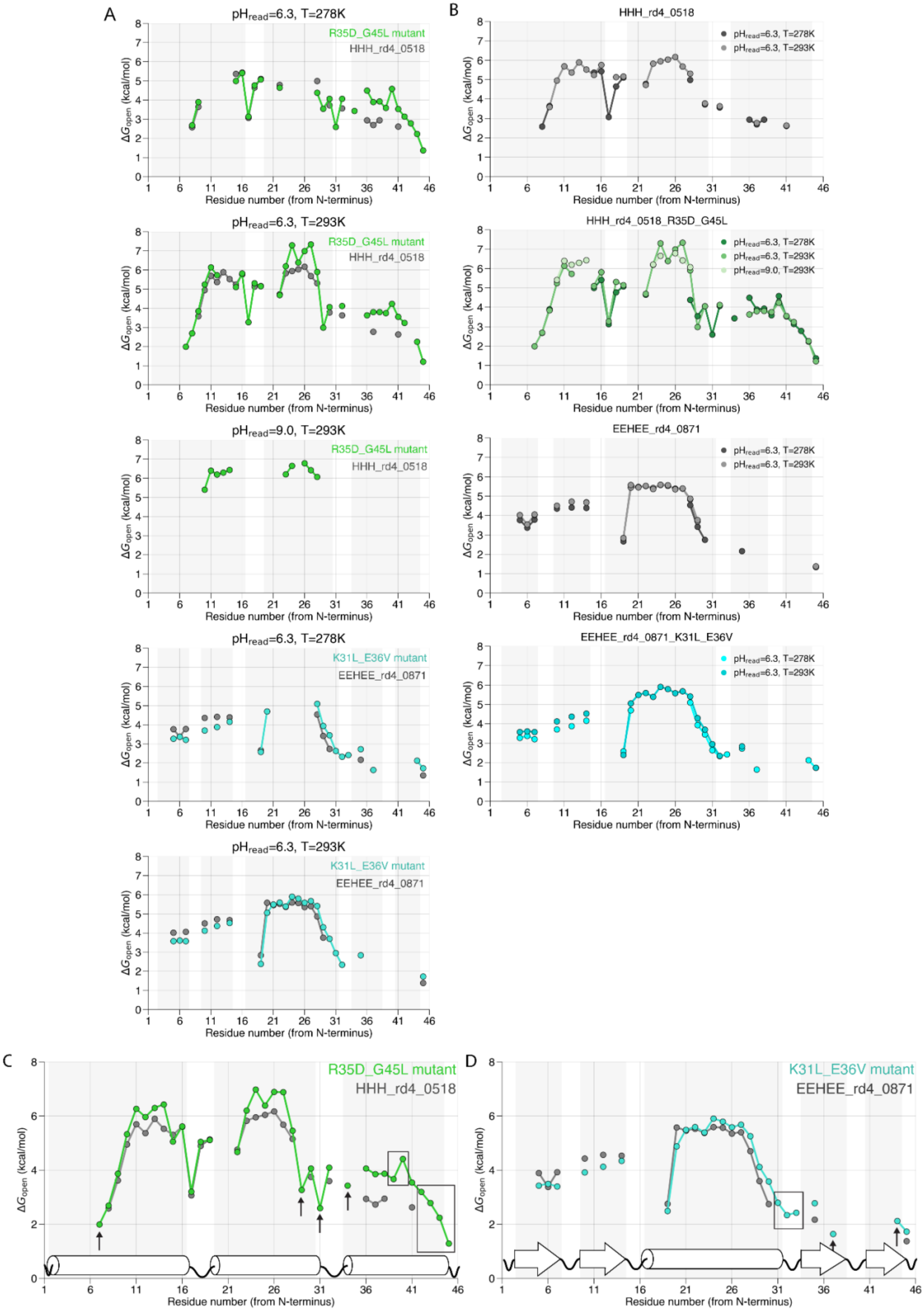
A
B
C
D
pH$_{read}$=6.3, T=278K
pH$_{read}$=6.3, T=293K
pH$_{read}$=9.0, T=293K
R35D_G45L mutant
HHH_rd4_0518
K31L_E36V mutant
EEHEE_rd4_0871
HHH_rd4_0518_R35D_G45L
EEHEE_rd4_0871_K31L_E36V
ΔG_{open} (kcal/mol)
Residue number (from N-terminus)

Figure S12: Residue-level HDX NMR analysis of wildtype and selected mutant proteins. (A) HDX NMR collected under multiple experimental conditions for HHH_rd4_0518 (wildtype) and its mutant, labeled as R34D_G45L for simplicity, and for EEHEE_rd4_0871 (wildtype) alongside its mutant K31L_E36V. (B) Four sub-panels display the HDX NMR data for each protein, with different shades representing the different experimental conditions. (C) Average residue opening free energies (ΔG_{open}) for HHH_rd4_0518 and the R34D_G45L mutant are shown; for residues with protection measured under multiple conditions, the values are averaged. Boxes indicate groups of residues while arrows highlight individual residues where protection was observed in the mutant but not in the wildtype. (D) A similar comparison is presented for EEHEE_rd4_0871 and the K31L_E36V mutant.

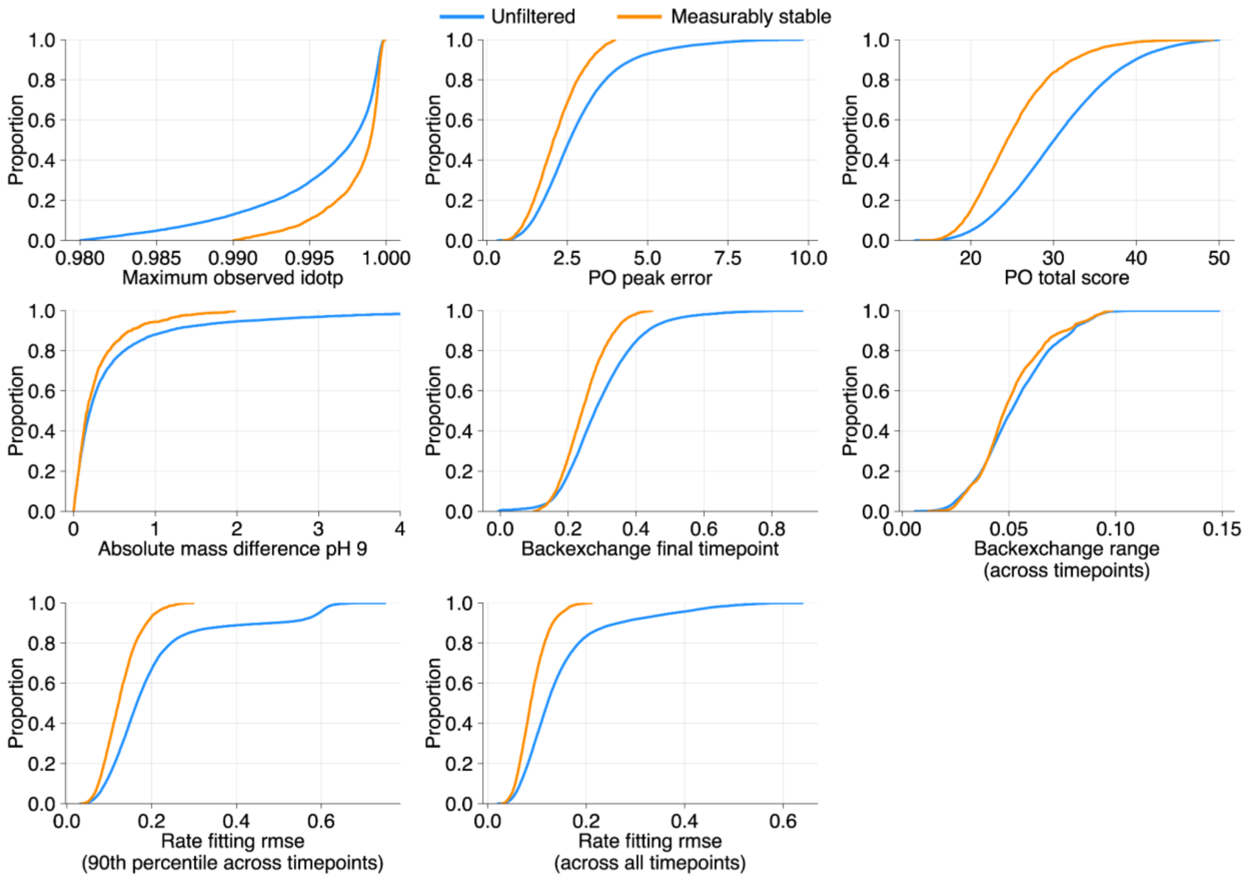

Figure S13: Cumulative distributions of quality control metrics. Blue curves show the cumulative distributions for 28,107 successful HDX measurements (note that individual proteins can be represented multiple times due to multiple retention times) that pass only the initial filtering criteria based on mass accuracy (<10 ppm), minimum isotopic distribution matching (idotp > 0.98), and a path optimizer (PO) total score (<50). Orange curves represent the cumulative distributions for the unique proteins selected to form the final dataset of 3,590 measurably stable proteins after applying additional, stricter quality control filters. These comparisons illustrate the impact of the combined filtering criteria on overall dataset quality. Notes: 1) the "absolute mass difference" is computed as the difference between the centroid mass at the last timepoint and that at the fifth-to-last timepoint after timepoint-specific back exchange correction; 2) the "back exchange range" is defined as the difference between the maximum and minimum back exchange values observed across all timepoints after applying timepoint-specific back exchange correction and, in the case of combined pH 6 and pH 9 data, experiment-wide back exchange normalization; 3) the rate fitting RMSE is computed as the root-mean-squared error between the theoretical isotopic distribution (based on the inferred k_{ex}) and the experimentally observed isotopic distribution, each scaled by dividing isotopic peak intensities by the most intense individual peak ($I/I_{max} = 1$); we report the 90th percentile of RMSE values across timepoints (second-to-last panel) and the average RMSE across timepoints (last panel).

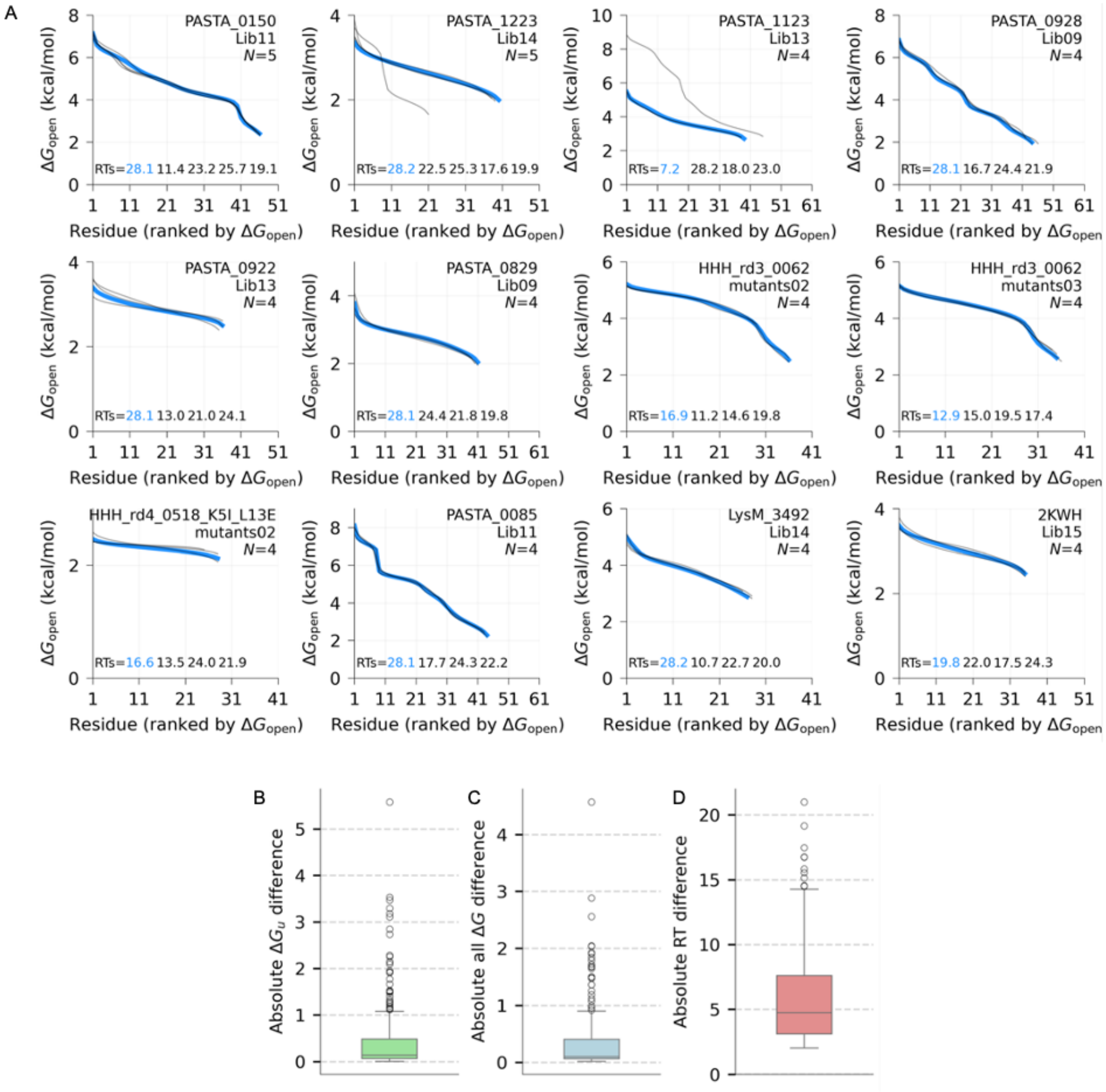

Figure S14: Reproducibility of mHDX-MS measurements across multiple retention times within the same library. (A) Examples of proteins exhibiting multiple retention times within a single experiment, yielding multiple successful HDX replicates. In total, 180 proteins were observed with at least two distinct retention times separated by more than 2 minutes. (B) Distribution of absolute differences in global stability (ΔG_{unfold}) between replicates. (C) Distribution of all measured ΔG values across replicates. (D) Distribution of retention time differences for proteins with replicates that differ by at least 2 minutes. Box-plots (B-D): center lines, median; boxes, 25th-75th percentiles; whiskers, 1.5x the interquartile range (to data limits). Outliers falling outside the whiskers are displayed.

Table S1: Datasets available (https://forms.gle/RwJwvfw6WN4gjXaD9)

Dataset name	Description	# of unique sequences
Dataset_0_InitialOrder<br><br>(Extended Data Fig. 4, Fig. S6)	Initial DNA from all libraries	15,715
Dataset_1_UnfilteredData<br><br>(Extended Data Fig. 4, Fig. S13)	HDX results minimally filtered based on confident IDs and PO score < 50	8,293
Dataset_2_SuccessfulHDX<br><br>(Fig. 1G, Extended Data Fig. 2, Extended Data Fig. 3, Extended Data Fig. 4, Extended Data Fig. 5, Extended Data Fig. 10, Fig. S4, Fig. S11, Fig. S12)	Successful HDX: proteins passing quality metrics described in the methods (includes EX1 kinetics and not fully deuterated proteins)	5,778
Dataset_3_MeasurablyStable<br><br>(Extended Data Fig. 4, Extended Data Fig. 9, Fig. 1C, Fig. 2A-F, Fig. 5B-D, Fig. S3, Fig. S5, Fig. S7, Fig. S11, Fig. S13)	Measurably Stable: proteins that reached full deuteration, ΔG_{unfold} was greater than 2 kcal/mol, EX1 kinetics removed	3,590
Dataset_4_HDXNMR<br><br>(Fig. 1H, Fig. 3C and Fig. 5F, Extended Data Fig. 7, Fig. S2, Fig. S5, Fig. S8, Fig. S12)	HDX NMR results: comprehensive dataset per condition, and average ΔG_{open} per position are provided as separate rows	16<br>(13 proteins including some with/without His-tags)
Dataset_5_MesophilicThermophilic<br><br>(Fig. 2G)	Subset of proteins from natural domains from Dataset_3 which we classified as mesophilic or thermophilic (opt. growth temperature > 40°C)	1,637
Dataset_6_splits_interpretable<br><br>(Fig. 4, Extended Data Fig. 8, Extended Data Fig. 9, Fig. S9)	Splits used for machine learning along with set of interpretable features	3,193
Dataset_6_splits_esm2<br>(Extended Data Fig. 9)	Splits used for machine learning along with set of ESM2-derived features	3,465
Dataset_6_splits_unirep<br>(Extended Data Fig. 9)	Splits used for machine learning along with set of Unirep features	3,465

Dataset_6_splits_saprot<br>(Extended Data Fig. 9)	Splits used for machine learning along with set of SaProt features	3,465
Dataset_6_splits_af_evoformer<br>(Extended Data Fig. 9)	Splits used for machine learning along with set of AF-Evoformer features	3,465
Dataset_7_mHDX_cDNA<br><br>(Extended Data Fig. 3)	Subset of proteins from Dataset_2 (best PO scored representative, excluded EX1 kinetics) that overlaps with available data from cDNA proteolysis assay [1]	4,464
Dataset_8_PDFs	Comprehensive set of plots generated by mhdx_pipeline and hdxrate_pipeline to evaluate time dependent mass distributions and their fits to rates. A jupyter-notebook is provided to help navigate through the pdfs.	-
Dataset_9_AlphaFoldModels<br>(Fig. S8)	Set of modeled structures from Dataset_2_SuccessfulHDX	5,778

Table S2: Parameters of cooperativity model fitting (Table S1: Dataset_3): $\Delta G_{\text{avg, expected}} = a\ (\Delta G_{\text{unf}} - b)^{c} \cdot (\text{fxn\_hb})^{d} + e\ (\text{netq})$

PF	N	a	b	c	d	e	Pearson correlation	RMSE (kcal/mol)
ααα	604	1.7614	1.6552	0.5245	0.2341	-0.0172	0.9368	0.2628
WW	253	0.7137	0.7551	1.0544	0.1236	-0.0285	0.9160	0.2280
LysM	1,004	1.5436	1.6800	0.6364	0.2597	-0.0241	0.9504	0.2584
ββαββ	329	1.6249	1.5824	0.6792	0.4643	0.0303	0.9358	0.3152
βαββ	170	1.5762	1.5393	0.5918	0.0607	-0.0184	0.9474	0.2267
PASTA	305	1.9200	1.6959	0.5624	0.4146	-0.0192	0.9561	0.3152
αββα	640	0.4650	-0.0533	1.1308	0.0808	-0.0093	0.8810	0.2744
Cold-Shock	60	1.2081	1.5092	0.6229	0.1626	-0.0215	0.8478	0.3567
PDB	85	1.9810	1.8122	0.5657	0.6322	-0.0181	0.9531	0.3381
all	3,465	1.4729	1.5818	0.5657	0.2569	-0.0082	0.9401	0.3142

References

1. Tsuboyama, K. *et al.* Mega-scale experimental analysis of protein folding stability in biology and design. *Nature* **620**, 434–444 (2023).
