# Supplementary material for: Large-scale discovery, analysis and design of protein energy landscapes
Source: Nature. 2026 May 13;654(8120):1108–18. doi: 10.1038/s41586-026-10465-z (PMC13293878; doi:10.1038/s41586-026-10465-z)
Supplement: Supplementary file 3 — Peer Review file [file 41586_2026_10465_MOESM3_ESM.pdf]

nature portfolio

Peer Review File

Large-scale discovery, analysis, and design of protein energy landscapes

Corresponding Author: Professor Gabriel Rocklin

Any redactions in this file are there to maintain patient confidentiality, the confidentiality of unpublished data, or to remove third-party material.

This file contains all reviewer reports in order by version, followed by all author rebuttals in order by version.

Attachments originally included by the reviewers as part of their assessment can be found at the end of this file.

Version 0:

Reviewer comments:

Referee #1

(Remarks to the Author)
This is a beautiful (I would say work of art!) paper that describes a tour-de-force effort to characterize small protein domains that are both designed and naturally occurring for their conformational heterogeneity (i.e. dynamic fluctuations). A very large number of domains (many thousands!) from 10 different families were analyzed. The authors were able to obtain high quality data on individual proteins in the large libraries, which is really new. The libraries were screened for stability towards proteolysis (developed in previous work) and amide H/D exchange (HDX). A really new development, and I would say the crux of this manuscript, is the high-throughput analysis of protein libraries by HDX-Mass spectrometry (HDX-MS). The workflow and subsequent HDX-MS data analysis was really impressive. The authors use ion mobility separation and analyze whole proteins over pH and time so as to achieve a complete exchange profile for each protein. They have deeply considered, and controlled for, all of the various issues that might plague such an analysis. I was especially impressed with the tensor factorization approach in which the retention time (RT), drift time (DT), and m/z dimensions of data are represented as a 3D tensor. They then apply an iterative rank-decomposition approach which disentangles the signals even when two of the three variables are overlapping. Finally, they implement a path optimizer which selects the most coherent set of isotopic clusters to generate a time-resolved mass profile. We see that the same protein is isolated because this analysis of the pH 6 experiments and the pH 9 experiments overlap in the middle (best seen in Figs S2 and S11). The HDX-MS data is also benchmarked for 13 proteins by NMR-based HDX experiments to characterize the exchange of each amide in the protein, with excellent agreement with the “whole protein” HDX-MS.

The authors use these data to determine global stability from the few most slowly exchanging amides. Their domains span a wide range of stabilities from less than 2 kcal/mol for many of the naturally-occurring domains (these are small domains) to almost 10 kcal/mol for the most stable designs. To me, the most exciting result from this work is the characterization of the conformational fluctuations, which occur at lower energies than the unfolding energy. HDX-MS data is uniquely capable of revealing these fluctuations, but since HDX-MS has been done one protein at a time, it was never possible to find “rules” that govern this behavior. The authors built an empirical model to predict the average energy of these conformational fluctuations, ΔG_{avg}, based on ΔG_{unfold}, hydrogen bonding, and protein net charge which was able to explain 89% of the variance in ΔG_{avg}. They then analyze the residuals to discover which domains have less than the average fluctuations (positive residual) or more than the average (negative residual). They interpret these results as higher cooperativity or lower cooperativity in the fluctuations. The results of this analysis reveal a large variation in cooperativity within each family indicating that sequence is the main determinant of cooperativity, not fold-type. They further analyzed some of the low cooperativity domains by HDX-NMR to reveal that the low stability residues were clustered, mostly into a single secondary structural element. One of the five examples did not have the low stability residues clustered. The results also showed that highly stable small domains can still have a low stability element present within them. These are very important results that deeply define protein folding landscapes, and that should be in all Biophysical Chemistry textbooks.

The manuscript ends with implementation of a machine learning model that, although not quantitative, will improve protein design workflows by making somewhat better predictions of improvement mutations.

I found the Discussion section underwhelming. It reads like a “potential pitfalls” section of a grant. I think the authors should rewrite their discussion focusing on what they learned rather than on mostly red-herring issues of the approach. To me, the key take-home messages are: 1) ion mobility uniquely enables library-scale HDX-MS analysis; 2) overall protein stability and conformational fluctuations are not thermodynamically linked; 3) many factors contribute to conformational fluctuations, which are still not predictable, whereas it is pretty easy now-a-days to predict overall protein stability and how to increase it.

Some minor corrections/comments

What is the dashed line in Fig. S8 supposed to represent? Please comment in the figure legend.

I think the first time the authors use RMSE (p 5) they should define this abbreviation.

The authors occasionally report RMSE values without units. RMSE values should always be reported with units - the same as the units of the variable being predicted (the target variable).

(Remarks on code availability)
The code looks very well done. It does include a README.

Referee #2

(Remarks to the Author)
Ferrari et al. introduce a new biophysical model that multiplexes hydrogen-exchange experiments of many proteins in order to measure the distribution of local folding energies (as defined as the log probability of the local unfolding of the secondary structure). The technology does not resolve site-specific information but instead obtains an unordered set of local folding free energies for all the amino acids of the protein, which can be ordered by magnitude and then represent a "fingerprint" of the protein's energy landscape. The authors also perform NMR-based hydrogen-exchange studied of a few selected proteins to correlated these fingerprints with site-specific information. The new method method represents a very interesting and exciting new trade-off between biophysical detail and scale of data. The contribution is extremely creative and original, and I believe it will be a valuable addition to a set of methods that can be used to obtain quantitatively reliable biophysical data for many proteins, and this is extremely important to power the next generation of biomolecular AI models. I strongly support the publication of this article in Nature. Please find below a few comments and suggestions on the writing and presentation of results.

- In various places the article discusses protein energy landscapes. I think it's fair to say that the method introduced here - as exciting as it is - doesn't really measure the energy landscape. Many energy landscapes map to the same fingerprint that's being measured.

- "However, these high-throughput methods do not yet have the ability to resolve the details of conformational fluctuations or identify the range of excited states populated by each protein
sequence." --> Why not? These models probably can resolve these states, especially models emulating the structure distribution such as BioEmu. They may just lack reliable data. Maybe the distinction here is between model and model parameters / model version?

- "These ΔGopen distributions also reveal the global stability (ΔGunfold) of each domain based on the opening energy of the most stable residue (Huyghues-Despointes, Scholtz, and Pace, 1999; Bai et al., 1994);" --> This may be my ignorance, but why is the opening energy of the most stable residue the global stability? Most methods that measure something like a global stability, e.g. via a melting curve, require some interpretation of the raw experimental data, often in the form of a two-state model. The stability obtained via such a measurement does not necessarily correspond to the stability of the most stable residue. In fact I find it plausible that the most stable residues are still formed in what most experiments would measure as unfolded state.

- In "Structural determinants of cooperativity" the authors tried correlating cooperativity with several protein features, and no strong correlation is found. My gut feeling is cooperativity must be at least a pairwise, if not many-body feature. Maybe it's worth trying to check correlation between the AlphaFold Evoformer pair embeddings and cooperativity, or trying to train a simple neural net from the pair embeddings to cooperativity.

- Similarly, the attempts to predict cooperativity from various features do not lead to high prediction accuracy. Can we be certain that the notion of cooperativity obtained from the measured fingerprints is accurate? For example, a completely cooperative 2-state folder would give to a nearly constant set of opening energies, but likewise would a completely uncooperative folder where all opening energies are the same range. I wonder what the uncertainty of this notion of cooperativity is - perhaps there is too much noise in the observable to fit a good model?

Frank Noe

(Remarks on code availability)

Referee #3

(Remarks to the Author)
The goal of this paper is to elucidate the conformational fluctuations in proteins under native conditions as they explore excited states. Clearly, such states could be (and are) very different from the globally unfolded states. Their characterization is important for several biological functions. As authors note in the text and through citations of literature, the idea that the free energies associated with the fluctuations span a very broad spectrum. They are protein dependent and could vary among proteins with similar stability, which implies that stability alone is insufficient to map out the energy landscape. HDX-MS is not a new method but the use of mHDX-MS that allows them to get data for hundreds of proteins appears to a new methodological development. With this new method they generated massive amount of data (high throughput), which I see as the major important highlight of the work. The results are potentially very useful. But as currently written, the paper leaves a lot to be desired.

Having stated this, there are many issues, both technical and conceptual, that the authors must address. The paper is very hard to read, period. (1) For starters there are no page numbers (Journal at some stage will fix it but does not help now) and equations are not numbered. Some equations are in in the figures – please move them to the text and explain the terms clearly using mathematical notation. (2) To analyze the data several techniques and computational methods are used. It is unclear how accurate are these methods separately and when combined. This will have an impact on the inferred DGopen,i for instance (Fig 1C for example). One does not get a feel for the possible errors at all.

Big picture: The question that keeps coming to mind is the following: What does one learn from the finding that all folded proteins make excursions to excited states in all proteins under native conditions? Conceptually, this is not new and is expected in finite systems, leading to the so what question. The authors correctly point out that the HDX NMR, slow but accurate method that can be used to determine the identity of residues, (seemed to be gold standard) has already shown that this is the case (pioneering works by Englander, Marqusee and others).

The authors suggest (in the Abstract and Discussion) that the data could improve machine learning and “physics-based” modeling. Besides being speculative, I do not know what “physics-based” (please explain) means, except it seems to pertain to MD simulations. The connection to aggregation, stated a few times, is not flushed out although this has been demonstrated on a limited basis using low throughput experiments (L. Kay and Clore Huntington) and simulations (for example Phys Rev Lett 105, 218101 (2010)). I think some relation between excited states to something concrete (aggregation or immunogenicity as they suggest) will be useful and possibly needed.

Definitions and computations: (1) It seems that the three free energy scale that relevant are in this work are: DGunfold is complicated (Eq. 5 in Fig. 1E). Is there any relation to the standard to GU - GN difference between the free energies of the folded and unfolded states measured by titration using denaturants for example? In 2023 in a Nature paper published a method to measure stability of proteins. They could determine the relation between stability and DGunfold. Somewhere in Methods they state DGunfold “as the average across five more stable residues”. What does this phrase mean? (2) The lengths of proteins studied are small (28-64). Fig. 1C shows that DGopen can be as large as 10 kcal/mol (roughly 16 kBT), which seems very large. (3) A precise definition forDGavg , which plays an important role throughout the paper, should be given using a simple equation. Similarly, define the Z-score using an equation instead of words because it seems to be not the standard definition in statistics (on first reading I could not determine if the Z-score is dimensionless) – you provide the distribution of normalized cooperativity in Fig. 2D.

Accuracy of mHDX-MS: As I understand it, the advance here is the development of multiplexed HDX-MS, which is illustrated in Fig. 1D and described briefly in one of the pages in the Methods. It would be good for the general reader to explain using a flow charr or an expanded version of Fig 1D and shown examples of the signal (m/z at different times). Also clarify what advance helped them to do this a high throughput manner. Extended Fig. 1 helps but it deals with analysis but mot the experimental protocol.

The number of domains validated using HDX-NMR is limited. While 13 domains were examined to benchmark the mHDX-MS results, this represents only a small fraction of the 5,778 domains analyzed. It is unclear whether the method's accuracy generalizes

across all domain families and stability regimes. I recognize that validating more domains via HDX-NMR is experimentally demanding, but the limited validation still presents a concern, especially given the advertised novelty of the method and the scale.

But even if we consider only these 13 domains, clarification is needed for the selection of the 13 validated domains. Were they chosen to represent the full range of stability, cooperativity, or sequence types observed in the dataset? Understanding the criteria for their inclusion would help assess the robustness and generalizability of the validation. Cooperativity: Although the notion of cooperativity plays an important role, there no crisp definition or clean connection to the measurements. There they introduce "normalized cooperativity" (Fig. 2D for example) and "family-normalized cooperativity" – please define using equations. I am insisting on these because (i) It plays a central role in the paper (use this idea in protein design); (ii) In the literature different papers use different definitions. Here it seems to be related to Z-score.

What can one make out of data in Fig. 2F for the two families? Naively might think there should be a relation at all between normalized cooperativity and DGunfold but Fig.2F seem indicate there is. Maybe they could calculate the distribution of the normalized cooperativity and some kind of normalized distribution DGunfold and then compute Kulback-Leibler divergence. Also, what the interpretation of the normalized cooperativity? Meaning of positive and negative values? Also, what about the other families in Fig. 2E?

Predictive models for opening cooperativity remain weak, implying that much of the landscape remains unexplained. The reliance on regularized linear models to predict folding stability and cooperativity is understandable, but alternative modeling strategies could offer deeper insights. For example, decision tree–based methods may better capture complex, nonlinear, and conditional relationships between structural or sequence features and experimental outcomes. These models are also interpretable, offering feature importance metrics that can reveal biophysically meaningful patterns and guide hypothesis generation more intuitively than linear coefficients.

Fig. 3: This figure deals primarily with the localization of stability. This information comes only from HDX NMR (Fig. 3C – is the size of the proteins the same?), which can be used to identify the identity and location of the residues in the 3D structure (Example Englander Cyt C and Marqusee T4 lysozyme). Both these studies show that unstable residues are localized in specific region. As I understand it, this information cannot be determined from mHDX MS. If so, could they not use the data in Fig. 3C to understand the data in Fig. 3B quantitatively and assess how the mHDX MS can provide structural information of the excited states? In the last paragraph of that section, they state that low opening cooperativity is in instable elements but not always. Does that mean one cannot be sure of what one learns from low cooperativity?

Fig. 4: This figure deals with structural features of cooperativity, which itself as I state a few times requires clarification. This figure and the associated discussion rely a lot on computational models. The PCCs are not impressive (A and B). What can be made of that? Also, how are DGunfold calculated using AlphaFold?

Fig. 5: The objective is to increase opening cooperativity in certain proteins using mutations using family-specific models. It is fine to create such mutants (Fig. S16). Once again, it is only the traditional HDX -NMR that is used to show increase in DGopen. Does nor not mean that the mHXD MS is not useful is designing or even understanding how to create high cooperative proteins?

The RMSD of the mutants from the WT in Figs A and D (predicted by AlphaFold) seems small. Is the case? Is that the reason for the inaccuracy of the machine learning results?

Methods: There are several things that were unclear. Let me focus on two. Consider the first equation (see also 2 in Fig. E). (1) Write an equation for the three variables on the RHS after labelling the equation. (2) It appears the LHS is taken from Englander and is independent of the residue identity. If this is so, state it and remove the subscript I from the LHS. (3) In Fig. S4 (are there not more than 11 proteins?) the correlation log kchem and log kHX is poor for most proteins as re the data in Fig S3. What are normalized define normalized values? The data are from HDX NMR? (3) Any rationale for the equation?

There is another equation that used in analysis. There are 5 parameters whose meaning is totally unclear to me. Of these it that DGunfoldD2O (Eq. 5 in Fig. E – I suppose

DGunfoldD2O = DGunfold used throughout the paper) from measurements via Fig 1E. I have asked for clarification (see above). The method for determining the parameters is not simple, making it difficult to understand the basis in which such choice (functional form was made).

After computing the parameters, the cooperativity is defined as the z-score of the residual between DGavg and DGavg,expected (the 5-parameter equation). Again, write an equation for z-score. Is = SDGopen,i/N?

Summary: I appreciate the need to get need for high throughput measurements. They must be accurate. As I noted, when they really need accurate measurements, they resort to the gold standard. In addition, the use of the mHDS MS data for any application is not clear expect for speculations in the Abstract and Discussion. In contrast, NMR relaxation experiments (far from being high throughput) (Science 336:(6079):362–6 (2012)) computations (JMB 426, 2653–2666 (2014)) have underscored the important of excited states and the structures in protein aggregation. I would be good if the authors could illustrate the knowledge of excited states could be used in predicting aggregation of proteins or other applications.

(Remarks on code availability)

Referee #4

(Remarks to the Author)
The manuscript by Ferrari et al. seeks to develop a novel method for multiplexed characterization of protein energy landscapes. To this end they employed hydrogen deuterium exchange mass spectrometry (HDX-MS). To enable multiplexed energy landscape characterization, this work employed DNA oligo pool libraries for the production of custom proteomes and developed a computational pipeline for automated analysis of the isotopic distribution for each domain at each exchange timepoint. These novel contributions enabled the development of the multiplex HDX-MS (mHDX-MS) method, employed in this paper to study variation in the energy landscapes of several natural and designed protein families. The authors determined this method to be efficacious given the correlation between data collected by mHDX-MS and HDX Nuclear Magnetic Resonance (HDX-NMR) spectroscopy, which is considered the gold standard for measurement of hydrogen deuterium exchange rates.

In summation, there are two aspects to this manuscript. First, it provides the methodology for analyzing the position-specific stabilities and segmental cooperativity of a wide array of proteins. Second, it provides a means of identifying organizing principles within and between different protein domains. With regard to the first aspect, I believe that the mHDX-MS method will have a significant impact on the field of protein biophysics due to its ability to test the generality of theories regarding the relationship between a protein’s energy landscape and its function. However, I find the second aspect somewhat wanting, because while the authors searched for protein features that correlated with protein folding cooperativity, none of the trends identified in this section were particularly convincing, leaving this reviewer somewhat underwhelmed, as I find myself falling back to the notion that for a method to be a breakthrough it needs to be able to reveal something about proteins that was previously not known, and here I don’t feel like I have learned much that the field didn’t already know. Maybe that there are no strong general rules is a discovery, but that’s not particularly satisfying. In addition to this shortfall, I have other major concerns.

Major concern 1:

Given the high-number of sequences that were unable to be analyzed because of their failures at different stages, how can the authors be sure that the cooperativity profiles that are measured are being uniformly sampled from the distribution of all possible cooperativity profiles? In other words, how can you be confident that the method isn’t selecting for proteins with energy landscapes that are not representative of the true distribution of energy landscapes across all proteins?

Major concern 2:

Given the weak correlations in Fig. 4 it appears that proteins with relatively extreme examples of each feature have been selected. For example, in the case of proline count the sequence chosen to represent 0 prolines has the lowest value of family-normalized cooperativity. Given the weak correlation and the extreme value chosen, what are the percent identities between the sequences chosen as representatives for panels b-h? Are the large differences in family-normalized cooperativity in these selected cases primarily explained by the feature of interest in that panel (i.e. in panels e and f are the sequences similar except for the number of prolines between them) or are there large differences in sequence between representatives. Additionally, how do the percent identities of these representatives compare to the average pairwise percent identity of their family?

Major concern 3:

When trying to identify structural determinants of cooperativity the authors state that the low Pearson correlation coefficients between family-normalized-cooperativity and individual features indicate the cooperativity is influenced by many factors;

however, when attempting to predict stability and cooperativity using multi-feature models the improvement in R2 appeared marginal relative to single feature predictors. For different families single features had a maximum R2 of 0.05-0.14, while multi-feature models had R2 of 0.16 to 0.24. This data doesn't necessarily support the conclusion that cooperativity is influenced by many factors given the relatively poor R2 for both single feature and multi-feature models, and instead suggests that the features chosen may simply be poor predictors of cooperativity.

Minor comment/question:

Minor comment 1: Are the correlations significant for the features used when examining structural determinants of cooperativity?

(Remarks on code availability)

Version 1:

Reviewer comments:

Referee #2

(Remarks to the Author)
The authors have addressed my questions and concerns and the article is ready for publication.

(Remarks on code availability)

Referee #3

(Remarks to the Author)

(Remarks on code availability)

Referee #4

(Remarks to the Author)
Upon evaluation of the revised manuscript I am still left underwhelmed. This manuscript is certainly an experimental achievement in terms of the scale that it achieves. It is clear that the path forward in terms of training language models is to develop experimental strategies that can provide a broad sequence/structure/energy space to train the model. This approach is a step in that direction. In the previous review, I listed some major concerns, which I will not repeat here. However, I will comment on how well the revised version addresses my concerns.

In short the fundamental shortcoming is that given the number of features they explored without finding any subset that explained much variance in cooperativity, I maintain my concern that perhaps the cooperativity metric isn't capturing anything meaningful about the behavior of proteins i.e. it's not actually reflective of differences in the folding cooperativity of proteins being studied.

Major concern 1: This concern still has not been addressed because while I agree with the authors' claim that they observe a wide variation in global stability and cooperativity, Fig. S10 highlights substantial variation in the success rate of mHDX-MS for different protein families. This suggests a selection bias, and the cause of this bias has not been identified. This apparent bias and overall low success rate prompts one to wonder whether the causes of these failures may also influence the ΔGopen values for those domains that could be experimentally characterized.

Major concern 2: I would still like to know the pairwise identities between the representative domains selected in Fig. 4 in addition to average pairwise identities in those families. While the authors have now added a line indicating that the examples in the figure are illustrative and that the differences in cooperativity do not owe solely to the highlighted feature, displaying the pairwise percent identities between selected variants would further emphasize this point.

Major concern 3: I feel that the edit made by the authors sufficiently addresses this concern.

(Remarks on code availability)

Open Access This Peer Review File is licensed under a Creative Commons Attribution 4.0 International License, which permits use, sharing, adaptation, distribution and reproduction in any medium or format, as long as you give appropriate credit to the original author(s) and the source, provide a link to the Creative Commons license, and indicate if changes were made.
In cases where reviewers are anonymous, credit should be given to 'Anonymous Referee' and the source.
The images or other third party material in this Peer Review File are included in the article's Creative Commons license, unless indicated otherwise in a credit line to the material. If material is not included in the article's Creative Commons license and your intended use is not permitted by statutory regulation or exceeds the permitted use, you will need to obtain permission directly from the copyright holder.
To view a copy of this license, visit https://creativecommons.org/licenses/by/4.0/

Referee #1:

This is a beautiful (I would say work of art!) paper that describes a tour-de-force effort to characterize small protein domains that are both designed and naturally occurring for their conformational heterogeneity (i.e. dynamic fluctuations). A very large number of domains (many thousands!) from 10 different families were analyzed. The authors were able to obtain high quality data on individual proteins in the large libraries, which is really new. The libraries were screened for stability towards proteolysis (developed in previous work) and amide H/D exchange (HDX). A really new development, and I would say the crux of this manuscript, is the high-throughput analysis of protein libraries by HDX-Mass spectrometry (HDX-MS). The workflow and subsequent HDX-MS data analysis was really impressive. The authors use ion mobility separation and analyze whole proteins over pH and time so as to achieve a complete exchange profile for each protein. They have deeply considered, and controlled for, all of the various issues that might plague such an analysis. I was especially impressed with the tensor factorization approach in which the retention time (RT), drift time (DT), and m/z dimensions of data are represented as a 3D tensor. They then apply an iterative rank-decomposition approach which disentangles the signals even when two of the three variables are overlapping. Finally, they implement a path optimizer which selects the most coherent set of isotopic clusters to generate a time-resolved mass profile. We see that the same protein is isolated because this analysis of the pH 6 experiments and the pH 9 experiments overlap in the middle (best seen in Figs S2 and S11). The HDX-MS data is also benchmarked for 13 proteins by NMR-based HDX experiments to characterize the exchange of each amide in the protein, with excellent agreement with the "whole protein" HDX-MS.

The authors use these data to determine global stability from the few most slowly exchanging amides. Their domains span a wide range of stabilities from less than 2 kcal/mol for many of the naturally-occurring domains (these are small domains) to almost 10 kcal/mol for the most stable designs. To me, the most exciting result from this work is the characterization of the conformational fluctuations, which occur at lower energies than the unfolding energy. HDX-MS data is uniquely capable of revealing these fluctuations, but since HDX-MS has been done one protein at a time, it was never possible to find "rules" that govern this behavior. The authors built an empirical model to predict the average energy of these conformational fluctuations, ΔGavg, based on ΔGunfold, hydrogen bonding, and protein net charge which was able to explain 89% of the variance in ΔGavg. They then analyze the residuals to discover which domains have less than the average fluctuations (positive residual) or more than the average (negative residual). They interpret these results as higher cooperativity or lower cooperativity in the fluctuations. The results of this analysis reveal a large variation in cooperativity within each family indicating that sequence is the main determinant of cooperativity, not fold-type. They further analyzed some of the low cooperativity domains by HDX-NMR to reveal that the low stability residues were clustered, mostly into a single secondary structural element. One of the five examples did not have the low stability residues clustered. The results also showed that highly stable small domains can still have a low stability element present within them. These are very important results that deeply define protein folding landscapes, and that should be in all Biophysical Chemistry textbooks.

The manuscript ends with implementation of a machine learning model that, although not quantitative, will improve protein design workflows by making somewhat better predictions of improvement mutations.

I found the Discussion section underwhelming. It reads like a 'potential pitfalls" section of a grant. I think the authors should rewrite their discussion focusing on what they learned rather than on mostly red-herring issues of the approach. To me, the key take-home messages are: 1) ion mobility uniquely enables library-scale HDX-MS analysis; 2) overall protein stability and conformational fluctuations are not thermodynamically linked; 3) many factors contribute to conformational fluctuations, which are still not predictable, whereas it is pretty easy now-a-days to predict overall protein stability and how to increase it.

Thank you for these suggestions to improve the discussion section. We significantly revised the first paragraph of the discussion, breaking into four key points. This includes adding the reviewer's point #2 that stability and conformational fluctuations were not necessarily linked, and being more explicit about the reviewer's point #3 that fluctuations were difficult to predict. The first paragraph of the discussion now reads:

It is widely appreciated that proteins are constantly in motion, continuously shifting between numerous conformational states on their energy landscapes. Yet the energetic details of these states — and the sequence features mediating them — remain almost entirely unknown. The mHDX-MS method makes it possible to experimentally analyze these fluctuations on a far larger scale than was previously possible, enabling new approaches to understand and predict fluctuations across sequence space. Several key findings emerge. First, our experiments revealed hidden variation in energy landscapes across 3,590 designed and natural domains, including substantial variation between related sequences. In fact, the variation within each fold family due to differences in sequence was often larger than the average difference between fold families (**Fig. 2E**). Second, we found that low cooperativity domains in our set often had entire pieces of secondary structure that were much less stable than the overall fold (**Fig. 3**). These domains were not especially unusual: they represent the lowest 25%ile (HHH_rd4_0518), 22% ile (EEHEE_rd4_0871), and 6%ile (LysM_0873) opening cooperativity scores in their families. Hundreds of domains in our set showed similarly low opening cooperativity, and these instabilities remain largely invisible to methods that predict native protein structures. Third, we found that global stability and local stability were partially uncoupled even in these small, compact domains: the domains with the highest folding stability (ΔG_{unfold}) in our set were not necessarily the most stable throughout their entire structures, as indicated by ΔG_{open} distributions that "cross over" in **Fig. 2D** and **3B.** Finally, we found that conformational fluctuations remain challenging to predict even given substantial experimental data. Although we discovered general protein properties with statistically significant correlations with cooperativity, our best models could predict only a limited fraction (16-24%) of the variance in opening cooperativity. Given experimental noise, we would expect perfect models to show correlations of R^2=0.74-0.78, indicating how much remains to be discovered about the determinants of conformational fluctuations.

Although our analysis employed ion mobility, we are not actually sure how critical this factor was, so we chose not to highlight it in the discussion.

The second paragraph of the discussion does discuss limitations of the work, and we agree that many of these issues are somewhat "red herrings" and do not affect the major analysis or conclusions. Still, we wanted to be explicit about these limitations in the discussion to ensure these limitations are fully understood by other researchers who might re-analyze or re-use these data and to be fully transparent about what our analysis can and cannot show.

Some minor corrections/comments

What is the dashed line in Fig. S8 supposed to represent? Please comment in the figure legend.

Thank you for pointing out this omission. The black dashed line corresponds to y = x, i.e. where the ΔG_{unfold} values measured by multiplex HDX-MS equal those obtained from cDNA-display proteolysis. We have added this explanation to the legend in the revised Fig. S8.

I think the first time the authors use RMSE (p 5) they should define this abbreviation.

The authors occasionally report RMSE values without units. RMSE values should always be reported with units - the same as the units of the variable being predicted (the target variable).

Thank you for catching both issues. We have made the following edits:

L190: RMSE is defined

L1165: Add clarification distributions are normalized to unit height to indicate RMSE is dimensionless

Added kcal/mol to the RMSE column in Table S2.

Referee #1 (Remarks on code availability):

The code looks very well done. It does include a README.

We appreciate the positive feedback on the code and documentation.

Referee #2:

Ferrari et al. introduce a new biophysical model that multiplexes hydrogen-exchange experiments of many proteins in order to measure the distribution of local folding energies (as defined as the log probability of the local unfolding of the secondary structure). The technology does not resolve site-specific information but instead obtains an unordered set of local folding free energies for all the amino acids of the protein, which can be ordered by magnitude and then

represent a "fingerprint" of the protein's energy landscape. The authors also perform NMR-based hydrogen-exchange studied of a few selected proteins to correlated these fingerprints with site-specific information. The new method method represents a very interesting and exciting new trade-off between biophysical detail and scale of data. The contribution is extremely creative and original, and I believe it will be a valuable addition to a set of methods that can be used to obtain quantitatively reliable biophysical data for many proteins, and this is extremely important to power the next generation of biomolecular AI models. I strongly support the publication of this article in Nature. Please find below a few comments and suggestions on the writing and presentation of results.

- In various places the article discusses protein energy landscapes. I think it's fair to say that the method introduced here - as exciting as it is - doesn't really measure the energy landscape. Many energy landscapes map to the same fingerprint that's being measured.

We agree that mHDX-MS, like any biophysical assay, measures a projection rather than a full reconstruction of the multidimensional energy landscape. We reviewed each use of the term "energy landscape" in the manuscript and removed references to directly measuring energy landscapes. In many cases, we now refer to "profiling energy landscapes" instead of "revealing" them. We made the following changes:

L48: "Our dataset enables new machine learning-based analysis of protein energy landscapes, and our experimental approach promises to profile these landscapes at unprecedented scale."
L206: "Profiling energy landscapes across domain families"
L228: "Figure 1: Multiplex hydrogen-deuterium exchange mass spectrometry (mHDX-MS) to profile energy landscapes"
L538: "This highlights the relative difficulty of predicting fine differences in opening energy profiles"

In other cases, we kept statements such as "our experiments revealed hidden variation in energy landscapes" (Discussion, L639) unchanged. We believe the diversity of opening energy profiles really does reveal a diversity of energy landscapes, even though (as the reviewer points out) many possible energy landscapes can correspond to the same opening energy profile).

- "However, these high-throughput methods do not yet have the ability to resolve the details of conformational fluctuations or identify the range of excited states populated by each protein sequence." --> Why not? These models probably can resolve these states, especially models emulating the structure distribution such as BioEmu. They may just lack reliable data. Maybe the distinction here is between model and model parameters / model version?

Thank you for pointing out the ambiguity. Our intent was to refer to the experimental high-throughput assays, not computational modeling approaches. We revised the introduction sentence to clarify this:

L86: "However, these high-throughput experimental methods do not yet have the ability to resolve the details of conformational fluctuations or identify the range of excited states populated by each protein sequence."

- "These ΔGopen distributions also reveal the global stability (ΔGunfold) of each domain based on the opening energy of the most stable residue (Huyghues-Despointes, Scholtz, and Pace, 1999; Bai et al., 1994);" --> This may be my ignorance, but why is the opening energy of the most stable residue the global stability? Most methods that measure something like a global stability, e.g. via a melting curve, require some interpretation of the raw experimental data, often in the form of a two-state model. The stability obtained via such a measurement does not necessarily correspond to the stability of the most stable residue. In fact I find it plausible that the most stable residues are still formed in what most experiments would measure as unfolded state.

Thank you for pointing out the need to clarify this section. Just like a melting curve is interpreted in terms of a two-state model, the raw HDX data (an exchange rate) are also interpreted using a two-state model. In this model, residues are in equilibrium between an exchange-incompetent closed state and an exchange-competent open-state where exchange occurs at the rate k_{chem}. The references in this section (Huyghues-Despointes and Bai) make the empirical observation that for the most stable residue in most proteins, the exchange-competent "open" state is thermodynamically identical to the globally unfolded state observed by other methods. However, as the reviewer points out, it is possible that protein unfolded states observed by traditional methods (not HDX) may include some minor residual structure that would still protect against H-D exchange, leading to thermodynamic differences in measured unfolding energies. We added a line and a reference clarifying that these effects are likely rare and small in magnitude:

L159: "These ΔG_{open} distributions also reveal the global stability (ΔG_{unfold}) of each domain based on the opening energy of the most stable residue (Huyghues-Despointes, Scholtz, and Pace 1999; Bai et al. 1994); to reduce noise we average the five most stable residues (**Fig. 1E:** Eq. 5 and **Fig. S4**). Note that residual HDX protection in the unfolded state may bias these estimates, though empirically this effect is small (<1 kcal/mol, Sosnick & Baxa 2025). Our experiments and analysis protocol produce reproducible $k_{HX,}$ ΔG_{open}, and ΔG_{unfold} measurements when the same domains are analyzed in different libraries (**Fig. 1G, Fig. S5**)."

- In "Structural determinants of cooperativity" the authors tried correlating cooperativity with several protein features, and no strong correlation is found. My gut feeling is cooperativity must be at least a pairwise, if not many-body feature. Maybe it's worth trying to check correlation between the AlphaFold Evoformer pair embeddings and cooperativity, or trying to train a simple neural net from the pair embeddings to cooperativity.

Thank you for the suggestion. We extracted the pair representation tensor from the AlphaFold2 Evoformer module and applied both mean and max pooling across residue pairs to generate per-residue features. These were then used as input to a neural network trained using the same architecture and evaluation protocol as in our benchmarking analyses. When trained across

topologies, models based on Evoformer pair embeddings achieved performance comparable to that of protein language models for ββαββ topology, but generally underperformed for other topologies. Notably, models trained within individual topologies using these features failed to generalize effectively across topologies. These results suggest that while pairwise structural representations do contain relevant information, they may not offer an advantage over sequence-based models that have been pretrained on broader evolutionary diversity, such as ESM2 or SaProt. It may also be that advantages of the pairwise structural models are difficult to establish using limited amount of data we were able to obtain.

We revised Supplemental Figure S17B to include the results from AF2-Evoformer. Overall, we don't believe that we have fully exhausted the machine learning possibilities of our dataset, but additional analysis is beyond the scope of this work. Publishing the full experimental dataset promptly will enable the full community to apply a broad range of approaches to discover how best to predict these differences in energy landscapes.

- Similarly, the attempts to predict cooperativity from various features do not lead to high prediction accuracy. Can we be certain that the notion of cooperativity obtained from the measured fingerprints is accurate? For example, a completely cooperative 2-state folder would give to a nearly constant set of opening energies, but likewise would a completely uncooperative folder where all opening energies are the same range. I wonder what the uncertainty of this notion of cooperativity is - perhaps there is too much noise in the observable to fit a good model?

We agree with the reviewer that a completely uncooperative protein with opening energies in the same range would appear fully cooperative to our analysis. However, in this study we are exclusively examining single domain proteins that form compact structures. Within this limited scope, we are unaware of any protein domain that has ever been characterized to be "completely uncooperative", suggesting that our interpretation of cooperativity from our opening energy profiles is reasonable.

Thank you as well for pointing out the need to clarify the uncertainty in our cooperativity metric. Due to the already long paragraph explaining normalized cooperativity in the results section, we felt the best place to describe the measurement error was the caption of Fig 2. This caption now notes:

L355: "Scale bars show experimental mean absolute deviations (MAD) between replicates measured in different libraries (0.2 kcal/mol for stability and 0.3 standard deviations for normalized cooperativity)."

Referee #3:

The goal of this paper is to elucidate the conformational fluctuations in proteins under native conditions as they explore excited states. Clearly, such states could be (and are) very different from the globally unfolded states. Their characterization is important for several biological functions. As authors note in the text and through citations of literature, the idea that the free energies associated with the fluctuations span a very broad spectrum. They are protein dependent and could vary among proteins with similar stability, which implies that stability alone is insufficient to map out the energy landscape. HDX-MS is not a new method but the use of mHDX-MS that allows them to get data for hundreds of proteins appears to a new methodological development. With this new method they generated massive amount of data (high throughput), which I see as the major important highlight of the work. The results are potentially very useful. But as currently written, the paper leaves a lot to be desired.

We thank the reviewer for the thoughtful assessment. We are pleased that they highlight (i) the biological importance of conformational fluctuations that lie beyond global unfolding, (ii) the decades-long difficulty of characterizing these heterogeneous excited states at scale, and (iii) the contribution of our mHDX-MS workflow, which now delivers high-throughput, protein-wide exchange landscapes profiles for hundreds of domains in a single experiment. These points underscore the significance of our study. Below, we address each of the reviewer's specific questions and clarify the areas that caused confusion.

Having stated this, there are many issues, both technical and conceptual, that the authors must address. The paper is very hard to read, period. (1) For starters there are no page numbers (Journal at some stage will fix it but does not help now) and equations are not numbered. Some equations are in in the figures – please move them to the text and explain the terms clearly using mathematical notation. (2) To analyze the data several techniques and computational methods are used. It is unclear how accurate are these methods separately and when combined. This will have an impact on the inferred DGopen,i for instance (Fig 1C for example). One does not get a feel for the possible errors at all.

We appreciate the reviewer's concerns about readability and transparency and have revised the manuscript to clarify these points: (A) Page/line numbering: the revised PDF now includes continuous line numbers so the reviewer can quickly locate every reference cited below. (B) Equation identification: all core expressions are shown in Fig. 1E, are labelled Eq. 1–5, and every variable is defined in the same caption (L240-249). The formula for the average opening free energy ($\Delta \boldsymbol{G}_{\text{avg}}$) is introduced in the manuscript at lines 272, and the terms for the multivariate model in Fig. 2B are likewise defined in that figure's caption (L349-351) and discussed in the main text (lines 283–306). Detailed derivations of the normalized- and family-normalized cooperativity metrics are provided in the Methods section "Deriving normalized cooperativity and family-normalized cooperativity" (lines 1184–1254). Overall and considering other similar papers in Nature, we felt the main text would be overly lengthy with each equation described in the main text. (C) Accuracy assessment: reproducibility is shown in Fig. 1G. Based on the reviewers suggestion, we expanded the main text description on this point:

L164: "Our experiments and analysis protocol reproducible $k_{HX,}$ ΔG_{open}, and ΔG_{unfold} measurements when the same domains are analyzed in different libraries, with opening energy profiles typically reproducible to within 0.2 kcal/mol mean average deviation (MAD, **Fig. 1G, Fig. S5**, and see Methods for a full discussion of error analysis)."

Along with the reproducibility of our approach, the accuracy compared with other measurements is also discussed in the section "**Multiplexed HDX-MS measurements are accurate".**

Big picture: The question that keeps coming to mind is the following: What does one learn from the finding that all folded proteins make excursions to excited states in all proteins under native conditions? Conceptually, this is not new and is expected in finite systems, leading to the so what question. The authors correctly point out that the HDX NMR, slow but accurate method that can be used to determine the identity of residues, (seemed to be gold standard) has already shown that this is the case (pioneering works by Englander, Marqusee and others).

We agree with the reviewer that the existence of conformational excursions to excited states is well established, and that this is not an important contribution of our study. However, we respectfully argue that our study is unique in quantifying these fluctuations across thousands of sequences (>500-fold larger than previous studies), observing wide variability, characterizing sequence contributions to this variability that hold across hundreds of sequences and were previously unknown, and applying these findings as a proof of concept in protein design. The analysis in Figure 2 comparing thousands of sequences in different families, the feature-based analysis in Figure 4, and the data-driven protein design in Figure 5 would all be impossible using the previously existing experimental data on conformational fluctuations. Furthermore, merely identifying simple domains with energy landscapes as distinct as those shown in Figure 3 would be very challenging without a rapid method for examining each domain. Beyond these findings, our new experimental approach now introduces the capability to expand large-scale studies of fluctuations even further.

The authors suggest (in the Abstract and Discussion) that the data could improve machine learning and "physics-based" modeling. Besides being speculative, I do not know what "physics-based" please explain) means, except it seems to pertain to MD simulations. The connection to aggregation, stated a few times, is not flushed out although this has been demonstrated on a limited basis using low throughput experiments (L. Kay and Clore Huntington) and simulations (for example Phys Rev Lett 105, 218101 (2010)). I think some relation between excited states to something concrete (aggregation or immunogenicity as they suggest) will be useful and possibly needed.

We agree with the reviewer that the boundary between "physics based" models and other types of modeling is not well defined. Still, we find this term commonly used in the literature to refer to models where the mathematical structure of the model (and often the parameters themselves) reflect specific physical assumptions and approximations, such as in the case of classical force fields used in molecular dynamics simulations. Ultimately, for length we decided not to expand further on this in the text.

As the reviewer notes, conformational fluctuations have been linked in the literature with both aggregation and immunogenicity (references Dumoulin et al. 2005; Codina et al. 2019; Volz et al. 2025; Pal and Udgaonkar 2024a, 2024b, de Taeye et al. 2015; Calvaresi et al. 2024).

Definitions and computations: (1) It seems that the three free energy scale that relevant are in this work are: DGunfold is complicated (Eq. 5 in Fig. 1E). Is there any relation to the standard to GU - GN difference between the free energies of the folded and unfolded states measured by titration using denaturants for example? In 2023 in a Nature paper published a method to measure stability of proteins. They could determine the relation between stability and DGunfold. Somewhere in Methods they state DGunfold "as the average across five more stable residues". What does this phrase mean? (2) The lengths of proteins studied are small (28-64). Fig. 1C shows that DGopen can be as large as 10 kcal/mol (roughly 16 kBT), which seems very large. (3) A precise definition forDGavg , which plays an important role throughout the paper, should be given using a simple equation. Similarly, define the Z-score using an equation instead of words because it seems to be not the standard definition in statistics (on first reading I could not determine if the Z-score is dimensionless) – you provide the distribution of normalized cooperativity in Fig. 2D.

The relationship between $\Delta \boldsymbol{G}_{unfold}$ and the most stable residues is described in the literature, as stated In L159: "These $\Delta \boldsymbol{G}_{open}$ distributions also reveal the global stability ($\Delta \boldsymbol{G}_{unfold}$) of each domain based on the opening energy of the most stable residue (Huyghues-Despointes, Scholtz, and Pace 1999; Bai et al. 1994); to reduce noise we average the five most stable residues (Fig. 1E: Eq. 5 and Fig. S4)."

We thank the reviewer for pointing to the need for explicit definitions for $\Delta \boldsymbol{G}_{avg}$ and z-score. We revised the manuscript to define $\Delta \boldsymbol{G}_{avg}$ in Figure 2A. We also define $\Delta \boldsymbol{G}_{avg}$ in **Quantifying opening cooperativity using opening free energy distributions** (L262) and in the Methods section **Deriving normalized cooperativity and family-normalized cooperativity** (L1184). For further clarity, we revised the main text (L297): "We define each domain's normalized residual (a z-score in units of standard deviations, see Methods) as its ***normalized cooperativity***" and now explicitly include the corresponding equation in the Methods section (L292)**.**

Accuracy of mHDX-MS: As I understand it, the advance here is the development of multiplexed HDX-MS, which is illustrated in Fig. 1D and described briefly in one of the pages in the Methods. It would be good for the general reader to explain using a flow charr or an expanded version of Fig 1D and shown examples of the signal (m/z at different times). Also clarify what advance helped them to do this a high throughput manner. Extended Fig. 1 helps but it deals with analysis but mot the experimental protocol.

The number of domains validated using HDX-NMR is limited. While 13 domains were examined to benchmark the mHDX-MS results, this represents only a small fraction of the 5,778 domains analyzed. It is unclear whether the method's accuracy generalizes across all domain families and stability regimes. I recognize that validating more domains via HDX-NMR is experimentally

demanding, but the limited validation still presents a concern, especially given the advertised novelty of the method and the scale.

But even if we consider only these 13 domains, clarification is needed for the selection of the 13 validated domains. Were they chosen to represent the full range of stability, cooperativity, or sequence types observed in the dataset? Understanding the criteria for their inclusion would help assess the robustness and generalizability of the validation.

Based on the reviewers comments, we revised Figure 1D to clarify that all colonies go into one flask (Fig 1D part 2) and now show actual examples of signals (m/z at different times) in Fig 1D part 4. For length, we didn't expand the main text further on what enabled multiplexed HDX-MS, but this is described in lines 702-837, most notably the combination of pooled expression of synthetic libraries and LC-IMS-MS analysis that enable us to record intact-protein exchange profiles for hundreds of domains in a single run.

We also agree with the reviewer that residue-resolved NMR is demanding because it requires uniformly 13C/15N-labelled samples and extensive assignment work. We are unaware of any published papers in any journal that include HDX NMR data on this many unique constructs. Based on the reviewers comment, we revised the main text in the section **Multiplexed HDX-MS measurements are accurate** to note "These 13 domains were chosen for NMR analysis to span a range of topologies, folding stabilities, and ΔG_{open} distributions.". Specifically, this set spans a broad range of properties: ΔG_{unfold} = 4–9 kcal/mol and family-normalized cooperativity from -2 s.d. to +2 s.d. across five different folds - making it a good cross-section of the 3,590 landscapes in our full dataset.

Cooperativity: Although the notion of cooperativity plays an important role, there no crisp definition or clean connection to the measurements. There they introduce "normalized cooperativity" (Fig. 2D for example) and 'family-normalized cooperativity" – please define using equations. I am insisting on these because (i) It plays a central role in the paper (use this idea in protein design); (ii) In the literature different papers use different definitions. Here it seems to be related to Z-score. What can one make out of data in Fig. 2F for the two families? Naively might think there should be a relation at all between normalized cooperativity and DGunfold but Fig.2F seem indicate there is. Maybe they could calculate the distribution of the normalized cooperativity and some kind of normalized distribution DGunfold and then compute Kulback-Leibler divergence. Also, what the interpretation of the normalized cooperativity? Meaning of positive and negative values? Also, what about the other families in Fig. 2E?

Normalized cooperativity is defined in the methods section **Deriving normalized cooperativity and family-normalized cooperativity**. The formula of the z-score function is now included in the same methods section (L1202). The meaning of positive and negative values is described in the section **Quantifying opening cooperativity using opening free energy distributions**:

L289: "Domains with a negative residual (**Fig. 2A** and **2B**, blue) have low energy fluctuations (low ΔG_{open}) across many residues that lead to a low ΔG_{avg}, compared to what our model

expects given ΔG_{unfold} and hydrogen bonding. We define each domain's normalized residual (a z-score in units of standard deviations, see Methods) as its ***normalized cooperativity***; a metric reflecting the level of conformational fluctuations in the domain compared to the typical domain with similar stability in our dataset."

In the section **Quantifying opening cooperativity using opening free energy distributions**, we describe the findings in Figure 2F: (L331) "Although normalized cooperativity is (intentionally) nearly orthogonal to stability across all 3,590 stable domains (**Fig. 2D**), certain domain families showed clear relationships between stability and cooperativity within the family (**Fig. 2F**)."

Predictive models for opening cooperativity remain weak, implying that much of the landscape remains unexplained. The reliance on regularized linear models to predict folding stability and cooperativity is understandable, but alternative modeling strategies could offer deeper insights. For example, decision tree–based methods may better capture complex, nonlinear, and conditional relationships between structural or sequence features and experimental outcomes. These models are also interpretable, offering feature importance metrics that can reveal biophysically meaningful patterns and guide hypothesis generation more intuitively than linear coefficients.

We agree that more expressive, non-linear modeling strategies - such as decision tree–based approaches - could provide additional insights, particularly as the dataset continues to grow. While we explored some degree of feature expansion in the current manuscript (see **Methods, Training of machine learning models**), a comprehensive evaluation of alternative architectures is beyond the scope of this work.

Our primary aim here is to establish the dataset, provide a reproducible analysis pipeline, and offer a baseline set of interpretable models for cooperativity. By making freely available both the data and the accompanying code, we hope to enable the broader research community to explore a range of modeling strategies that may uncover additional mechanistic insights.

Fig. 3: This figure deals primarily with the localization of stability. This information comes only from HDX NMR (Fig. 3C – is the size of the proteins the same?), which can be used to identify the identity and location of the residues in the 3D structure (Example Englander Cyt C and Marqusee T4 lysozyme). Both these studies show that unstable residues are localized in specific region. As I understand it, this information cannot be determined from mHDX MS. If so, could they not use the data in Fig. 3C to understand the data in Fig. 3B quantitatively and assess how the mHDX MS can provide structural information of the excited states? In the last paragraph of that section, they state that low opening cooperativity is in instable elements but not always. Does that mean one cannot be sure of what one learns from low cooperativity?

We agree that mHDX-MS lacks the spatial resolution of HDX NMR and cannot directly localize stability to specific residues. This highlights an inherent trade-off between the biophysical detail of HDX NMR and the throughput and scale achievable with mHDX-MS. Nevertheless, the free energy profiles extracted from mHDX-MS provide unique and important information about the

variation in opening energy throughout a protein's structure. As a simple demonstration, the local instabilities highlighted in the domains in Fig. 3 could not have been discovered and identified in the first place without the efficient screening made possible by mHDX-MS.

As noted in the text (L407) "Overall, these results show that low opening cooperativity typically results from specific unstable elements of the structure - though not always. Bimodal ΔG_{open} distributions like those seen for HHH_rd4_0518, EEHEE_rd4_0871, and LysM_0873 may be evidence of spatial clustering of stability, but this need not always be the case."

Fig. 4: This figure deals with structural features of cooperativity, which itself as I state a few times requires clarification. This figure and the associated discussion rely a lot on computational models. The PCCs are not impressive (A and B). What can be made of that? Also, how are DGunfold calculated using AlphaFold?

We agree that the Pearson-correlation coefficients in Fig. 4A–B are modest; we note this in the text (L446): "No individual feature dominated the correlations (maximum |PCC| = 0.38 ± 0.07 for ααα and 0.27 ± 0.09 for ββαββ; mean ± 95 % CI from bootstrapping), indicating that cooperativity is influenced by many factors." To clarify, we do not use AlphaFold to calculate dGunfold, but rather correlate the ΔG_{unfold} measurements from mHDX-MS with different AlphaFold metrics (pLDDT and PAE), as shown in Fig 4A-B.

Fig. 5: The objective is to increase opening cooperativity in certain proteins using mutations using family-specific models. It is fine to create such mutants (Fig. S16). Once again, it is only the traditional HDX -NMR that is used to show increase in DGopen. Does nor not mean that the mHXD MS is not useful is designing or even understanding how to create high cooperative proteins?

The RMSD of the mutants from the WT in Figs A and D (predicted by AlphaFold) seems small. Is the case? Is that the reason for the inaccuracy of the machine learning results?

To clarify, the change in ΔG_{open} distributions is first shown by mHDX-MS (Fig. 5C, D) and then afterwards shown with improved spatial resolution by HDX NMR. While it may have been possible to discover cooperativity-increasing mutants by other methods, in this instance we successfully used the mHDX-MS data and machine learning models to discover these mutants. The impact of the data is shown by the different outcomes of the designed mutants compared to randomly selected double mutants (Fig. 5B, C).

Methods: There are several things that were unclear. Let me focus on two. Consider the first equation (see also 2 in Fig. E). (1) Write an equation for the three variables on the RHS after labelling the equation. (2) It appears the LHS is taken from Englander and is independent of the residue identity. If this is so, state it and remove the subscript I from the LHS.

Thank you for the suggestion to clarify these equations. We revised the caption of Fig. 1E to note that i refers to the residue index; this helps clarify that each residue has its own k_{HX}.

(3) In Fig. S4 (are there not more than 11 proteins?) the correlation log kchem and log kHX is poor for most proteins as re the data in Fig S3. What are normalized define normalized values? The data are from HDX NMR? (3) Any rationale for the equation?

Please see Methods, **Chemical intrinsic rates approximation in mHDX-MS analysis**: (L1064) "To improve on this approximation, we examined the empirical relationship between $\boldsymbol{k}_{\text{chem},\boldsymbol{i}}$ and $\boldsymbol{k}_{\text{HX},\boldsymbol{i}}$ using site-resolved HDX NMR data on 11 proteins (all from **Fig. S7** except double mutants HHH_rd4_0518_R35D_G45L and EEHEE_rd4_0871_K31L_E36V)." We note the quality of the correlations in Fig. S4 does not critically affect the results of the paper; our approach to inferring $\boldsymbol{k}_{\text{chem}}$ values is simply a small improvement compared with assuming the median $\boldsymbol{k}_{\text{chem}}$ for all residues.

There is another equation that used in analysis. There are 5 parameters whose meaning is totally unclear to me. Of these it that DGunfoldD2O (Eq. 5 in Fig. E – I suppose DGunfoldD2O = DGunfold used throughout the paper) from measurements via Fig 1E. I have asked for clarification (see above). The method for determining the parameters is not simple, making it difficult to understand the basis in which such choice (functional form was made).

After computing the parameters, the cooperativity is defined as the z-score of the residual between DGavg and DGavg,expected (the 5-parameter equation). Again, write an equation for z-score. Is = SDGopen,i/N?

Please refer to sections in the main text (L262) **Quantifying opening cooperativity using opening free energy distributions** and (L1184) Methods: **Deriving normalized cooperativity and family-normalized cooperativity** for a comprehensive motivation for building the 5-parameter model, its implications, and the Bayesian regression model description.

We revised the manuscript to include the z-score function in the same methods section (L1202).

Referee #4:

The manuscript by Ferrari et al. seeks to develop a novel method for multiplexed characterization of protein energy landscapes. To this end they employed hydrogen deuterium exchange mass spectrometry (HDX-MS). To enable multiplexed energy landscape characterization, this work employed DNA oligo pool libraries for the production of custom proteomes and developed a computational pipeline for automated analysis of the isotopic distribution for each domain at each exchange timepoint. These novel contributions enabled the development of the multiplex HDX-MS (mHDX-MS) method, employed in this paper to study variation in the energy landscapes of several natural and designed protein families. The authors determined this method to be efficacious given the correlation between data collected by mHDX-MS and HDX Nuclear Magnetic Resonance (HDX-NMR) spectroscopy, which is considered the gold standard for measurement of hydrogen deuterium exchange rates.

In summation, there are two aspects to this manuscript. First, it provides the methodology for analyzing the position-specific stabilities and segmental cooperativity of a wide array of proteins. Second, it provides a means of identifying organizing principles within and between different protein domains. With regard to the first aspect, I believe that the mHDX-MS method will have a significant impact on the field of protein biophysics due to its ability to test the generality of theories regarding the relationship between a protein's energy landscape and its function. However, I find the second aspect somewhat wanting, because while the authors searched for protein features that correlated with protein folding cooperativity, none of the trends identified in this section were particularly convincing, leaving this reviewer somewhat underwhelmed, as I find myself falling back to the notion that for a method to be a breakthrough it needs to be able to reveal something about proteins that was previously not known, and here I don't feel like I have learned much that the field didn't already know. Maybe that there are no strong general rules is a discovery, but that's not particularly satisfying. In addition to this shortfall, I have other major concerns.

Major concern 1:

Given the high-number of sequences that were unable to be analyzed because of their failures at different stages, how can the authors be sure that the cooperativity profiles that are measured are being uniformly sampled from the distribution of all possible cooperativity profiles? In other words, how can you be confident that the method isnt selecting for proteins with energy landscapes that are not representative of the true distribution of energy landscapes across all proteins?

We agree with the reviewer that the cooperativity profiles we measure are not uniformly sampled from the distribution of all possible cooperativity profiles. Indeed we are not sure how one could define the underlying true distribution of protein sequences. It is very difficult to state what is representative of "most" proteins, so we avoid claiming our domains are representative. Still, empirically we observe a wide variation in both global stability and in cooperativity among our domains (i.e. Fig. 2D), so if our selection is biased, it is not overly restrictive about which domains are able to be characterized. We note that our domains may not be representative at the end of the section **Structural determinants of cooperativity**:

"These correlations provide insight into the biophysical determinants of opening cooperativity, but they depend on the domains in our dataset, which are not necessarily representative of all folded domains or all possible sequences."

Major concern 2:

Given the weak correlations in Fig. 4 it appears that proteins with relatively extreme examples of each feature have been selected. For example, in the case of proline count the sequence chosen to represent 0 prolines has the lowest value of family-normalized cooperativity. Given the weak correlation and the extreme value chosen, what are the percent identities between the

sequences chosen as representatives for panels b-h? Are the large differences in family-normalized cooperativity in these selected cases primarily explained by the feature of interest in that panel (i.e. in panels e and f are the sequences similar except for the number of prolines between them) or are there large differences in sequence between representatives. Additionally, how do the percent identities of these representatives compare to the average pairwise percent identity of their family?

The examples in this figure are primarily to illustrate (1) how differences in features appear in three-dimensional protein structures, and (2) how widely ΔGopen distributions can vary even between different sequences sharing the same fold. We do not mean to claim that the differences in cooperativity within these examples owe entirely (or primarily) to the highlighted features. Hopefully this is clear from the overall modest strength of the features correlations (as pointed out by the reviewer). To clarify this further, we revised the caption of Figure 4 to note:

Examples in this figure are illustrative; the differences in cooperativity do not owe purely to one highlighted feature.

Major concern 3:

When trying to identify structural determinants of cooperativity the authors state that the low Pearson correlation coefficients between family-normalized-cooperativity and individual features indicate the cooperativity is influenced by many factors; however, when attempting to predict stability and cooperativity using multi-feature models the improvement in R2 appeared marginal relative to single feature predictors. For different families single features had a maximum R2 of 0.05-0.14, while multi-feature models had R2 of 0.16 to 0.24. This data doesnt necessarily support the conclusion that cooperativity is influenced by many factors given the relatively poor R2 for both single feature and multi-feature models, and instead suggests that the features chosen may simply be poor predictors of cooperativity.

Although the absolute R^2 values are modest, combining features increases the median cross-validated R^2 by a statistically significant ten-percentage-point gain. Still, to address this comment, we revised this statement on line 448:

This suggests that cooperativity is influenced by many factors, but could indicate that critical determinants of cooperativity are missing from our feature set.

Minor comment/question:

Minor comment 1: Are the correlations significant for the features used when examining structural determinants of cooperativity?

We revised the main text to note significance in the section **Structural determinants of cooperativity**:

The large number of features makes it likely that some correlations would be observed by chance, but many observed correlations were stronger than those found using permuted data, indicating statistical significance (the ranked observed correlations are outside the 95%ile confidence interval of correlations expected by chance, **Fig. S15**).

This email has been sent through the Springer Nature Manuscript Tracking System NY-610A-SN&MTS

Reviewer #4

Upon evaluation of the revised manuscript I am still left underwhelmed. This manuscript is certainly an experimental achievement in terms of the scale that it achieves. It is clear that the path forward in terms of training language models is to develop experimental strategies that can provide a broad sequence/structure/energy space to train the model. This approach is a step in that direction. In the previous review, I listed some major concerns, which I will not repeat here. However, I will comment on how well the revised version addresses my concerns.

In short the fundamental shortcoming is that given the number of features they explored without finding any subset that explained much variance in cooperativity, I maintain my concern that perhaps the cooperativity metric isn't capturing anything meaningful about the behavior of proteins i.e. it's not actually reflective of differences in the folding cooperativity of proteins being studied.

We agree that our models built from a large number of features show only a modest ability to explain normalized cooperativity (R^2=0.16-0.24). However, it does not logically follow that the normalized cooperativity metric isn't capturing anything meaningful about proteins. Normalized cooperativity can be measured with high reproducibility (replica error bars shown in Fig. 2D, 3B, and 5C-D). Importantly, many critical biophysical properties of proteins can be measured accurately but not predicted by any machine learning methods. Because our work takes the first step toward characterizing highly complex energy landscapes on a large scale, we believe that the modest correlations are more likely to result from insufficiently descriptive features and the limited data size (still >500x larger than previous efforts). Our work will catalyze further machine learning efforts (and new data generation) to improve prediction in this area. The difficulty of predicting normalized cooperativity highlights the frontier challenge of understanding and predicting protein conformational fluctuations.

The "meaningfulness" of normalized cooperativity is shown by the differences in opening energy landscapes in Fig. 2B and 3B. The proteins with low normalized cooperativity clearly possess conformational fluctuations measurable by HDX that are much lower in energy than their global folding stabilities. For most of these proteins with low normalized cooperativity, these fluctuations occur over entire secondary structural elements, such as the C-terminal hairpin in EEHEE_rd4_0871, C-terminal helix in HHH_rd4_0518, and helix 2 in LysM_0873. These fluctuations have never been measurable on a large scale prior to our study, and the normalized cooperativity metric distinguishes these proteins from superficially similar structures with differences in their HDX-measurable fluctuations.

As an additional, independent approach to analyzing our HDX data, we now include a new dimensionality-reduction analysis based on principal component analysis (PCA). When PCA is applied to our opening energy profile measurements within protein families, the first principal component is strongly correlated with global folding stability, and the second principal component is strongly correlated with the family-normalized cooperativity metric that we defined (Pearson r=0.61-0.80). This correspondence indicates that normalized cooperativity captures the dominant axis of variation in opening energy profiles after accounting for global stability. We have added this analysis to the manuscript and expanded the discussion to clarify the scope and interpretation of the cooperativity metric.

L322: "By defining normalized cooperativity as relative to a typical domain with the same stability using our empirical model, we decouple normalized cooperativity from stability (**Fig. 2D**), enabling us to analyze how protein features directly influence cooperativity independently from stability. This approach simplifies the multidimensional opening energy profiles down to two

nearly orthogonal dimensions: global folding stability (ΔG_{unfold}) and normalized cooperativity (**Fig. 2D**).

An alternative approach to simplify the multidimensional opening energy profiles is to apply principal component analysis (PCA). Naively applying PCA to our opening energy profiles (without considering protein length, hydrogen-bonding, or net charge) results in one dominant principal component which strongly correlates with ΔG_{unfold} (r = 0.92, **Fig. S13A**). When applying PCA within families of domains, the first principal component correlates with ΔG_{unfold} (r = 0.89-0.96 for the four largest families LysM, αββα, ααα, ββαββ, **Fig. S13B**), and the second principal component is strongly correlated with normalized cooperativity (r = 0.61-0.80, **Fig. S13B**). This indicates that normalized cooperativity captures the dominant variation between opening energy profiles, after removing the variation in ΔG_{unfold}. Whereas PCA analysis does not consider the biophysical origins of variation between opening energy profiles, normalized cooperativity isolates the differences in conformational fluctuations between proteins by removing variation caused by differences in protein length, hydrogen bonding, and net charge that do not relate to conformational fluctuations."

Major concern 1: This concern still has not been addressed because while I agree with the authors' claim that they observe a wide variation in global stability and cooperativity, Fig. S10 highlights substantial variation in the success rate of mHDX-MS for different protein families. This suggests a selection bias, and the cause of this bias has not been identified. This apparent bias and overall low success rate prompts one to wonder whether the causes of these failures may also influence the ΔGopen values for those domains that could be experimentally characterized.

We thank the reviewer for this concern regarding the variation in success rates across protein families. To accurately describe the possible reasons for different success rates between different families, we identified several quantifiable technical and biophysical indicators that partially explain this variation. These factors are now presented in the updated Fig. S10 panels D–I to identify technical and biophysical drivers of this variation. While we cannot fully predict the success rate of an individual sequence, we did identify several important factors:

1. Technical constraints. Different proteins were measured in different size libraries, and library size had a strong correlation with the success of individual proteins. We observe a significant negative correlation between the initial library size and the success rate for both protein identification (r = -0.60, Fig. S10D) and HDX mapping (r = -0.73, Fig. S10E). Mechanistically, this occurs because larger protein libraries have greater spectral crowding and increased ion suppression in the mass spectrometer. As library size increases, we also observed a decrease in the mean number of charge states identified per protein (r = -0.79, Fig. S10H). Crucially, the mean number of charge states identified is a near-perfect predictor of HDX success (r = 0.92, Fig. S10I). This demonstrates that many "failures" result from overcrowding in our larger protein libraries.

2. Biophysical differences: Using independent (non-HDX) measurements of folding stability from cDNA display proteolysis (Fig. S8), we find that the success rates of different protein families correlate positively with the median global stability ΔG_{unfold} of each family (r = 0.63 for MS ID and r = 0.60 for successful HDX; Fig. S10F–G). This indicates that proteins with higher baseline stability are more robust to the experimental workflow.

These features explain some of the differences in success rates between different families. As in traditional experiments on individual proteins, proteins with higher folding stability are more likely to produce successful results in our experiments, and larger library size leads to lower

success due to interference between different proteins. Still, even with these potential biases, our work captures wide variation in energy landscapes across thousands of different sequences for the first time, enabling machine learning based analysis of energy landscapes and successful redesign to minimize conformational fluctuations (Fig. 5).

Lastly, our manuscript does not claim that the domains we study are “representative” from a distribution of domains, and there is no well-defined “true distribution” of protein domains. The key interesting aspect of our study comes from the variation in fluctuations seen across the domains that we studied, enabling us to examine the sequence origins of this variation. Finally, it is important to note that this field has traditionally studied protein dynamics and conformational landscapes one protein at a time for individually selected proteins. These traditional single protein studies likely exhibit far more “selection bias” in which energy landscapes are measured and published than our new large-scale approach.

Major concern 2: I would still like to know the pairwise identities between the representative domains selected in Fig. 4 in addition to average pairwise identities in those families. While the authors have now added a line indicating that the examples in the figure are illustrative and that the differences in cooperativity do not owe solely to the highlighted feature, displaying the pairwise percent identities between selected variants would further emphasize this point.

We have now added the average pairwise identities to Fig. 4 to emphasize this point.

Major concern 3: I feel that the edit made by the authors sufficiently addresses this concern.

Reviewer #3

In the revised paper, the authors have clarified some aspects of the paper in response to the comments. However, some of the comments were ignored or only partially addressed. It appears that they have made only small changes to the paper to address the questions posed or concerns expressed. As I stated in the previous report (echoed by others) getting at the excited states of proteins on a large scale, as has been attempted for small proteins (less than 65 residues) is important. For this reason, I think that study has merit for sure.

However, questions remain for this Reviewer to be convinced that this article goes beyond methodological advance. Although several questions come to mind, which really is a sign of a thought-provoking paper, I will focus on a few. In no order I have the following additional comments, which I trust the authors will address. (In red color I quote questions raised in the previous report).

Big picture: The question that keeps coming to mind is the following: What does one learn from the finding that all folded proteins make excursions to excited states in all proteins under native conditions?

The authors did not address this question at all.

Respectfully, we are unsure why the reviewer felt we did not address this question in our previous response. Below, we include our previous response noting that this question misstates the primary finding and significance of our study.

“We agree with the reviewer that the existence of conformational excursions to excited states is well established, and that this is not an important contribution of our study. **However, we respectfully argue that our study is unique in quantifying these fluctuations across thousands of sequences (>500-fold larger than previous studies), observing wide variability, characterizing sequence contributions to this variability that hold across hundreds of sequences and were previously unknown, and applying these findings as a proof of concept in protein design.** The analysis in Figure 2 comparing thousands of sequences in different families, the feature-based analysis in Figure 4, and the data-driven protein design in Figure 5 would all be impossible using the previously existing experimental data on conformational fluctuations. Furthermore, merely identifying simple domains with energy landscapes as distinct as those shown in Figure 3 would be very challenging without a rapid method for examining each domain. Beyond these findings, our new experimental approach now introduces the capability to expand large-scale studies of fluctuations even further.”

We agree with the reviewer’s point and that the mere existence of excited states under native conditions is well established and not, by itself, a conceptual advance. As we emphasized in our initial response, this is not the primary claim of our work. Our work goes beyond observing fluctuations to observe how different sequences have highly differing levels of fluctuations. By scaling fluctuation measurements to thousands of domains, we uncover (i) large, previously unappreciated variability in native-state energy landscapes even among similarly folded proteins (Fig. 2–3), (ii) sequence features that systematically modulate these fluctuations across hundreds of homologs (Fig. 4), and (iii) the ability to deliberately reshape conformational landscapes through data-driven design (Fig. 5). Furthermore, our own machine learning analysis only scratches the surface of what is possible using our dataset. Our unprecedented large scale, quantitative data creates a foundation that enables the whole research community to investigate the origins and sequence-dependence of conformational fluctuations in far greater depth than ever before, using the full panoply of modeling and machine learning tools available for modeling proteins.

They suggested in the Introduction potential applications to among other things protein aggregation among other things. It would have been good if they had tried at argue how this could be done. I mentioned a paper in PRL and alluded to a study by L. Kay. They ignored both. Let me be more specific. (1) The importance of excited states in the context of aggregation was shown in Curr. Opin. Strut. Biol. 13: 146 (03). Scenarios for proteins whose ground states are folded (PrPC like the one studied by Pal and Udgaonkar – quoted in the current paper) were outlined. I think for historical accuracy and scholarly presentation the authors should be accurate in the citations. I was surprised that they ignored the suggestions. (2) More importantly, the important paper by study (Science, 336: 362 (2012)) in which the role of a lowly populated excited state in as triple mutant of Fyn SH3 aggregation was investigated. Using relaxation dispersion NMR measurements, they determined the structure of the excited state. It would be insightful if the authors could make a connection to this study. Given that SH3 is protein that the present authors consider. Clearly, such precision experiments cannot be carried out on large scale, but it would be comforting if the authors could make a connection to the L. Kay study.

Our introduction notes the previously established relationship between protein fluctuations and protein aggregation, although the goal of the current study is not specifically to analyze or protect against protein aggregation. We fully agree that the work by Neudecker, Kay, and colleagues (Science, 2012) is a landmark study that provides a high-resolution structural precedent for the thermodynamic states we are characterizing at scale and provides extra context to the role of excited states in protein aggregation. We have included this citation in the Introduction (along with the other important fundamental studies already mentioned: Dumoulin

et al. 2005; Codina et al. 2019; Volz et al. 2025; Pal and Udgaonkar 2024a, 2024b, de Taeye et al. 2015; Calvaresi et al. 2024). We also extended the Discussion:

L698: “Importantly, our goal is not to replace high-resolution NMR studies that can characterize individual excited states with residue-level precision (Neudecker et al. 2012), but rather to provide a scalable framework for mapping conformational fluctuation landscapes across sequence space, thereby contextualizing and prioritizing targets for detailed structural investigation.”

We believe this clarification more clearly positions our method as complementary to relaxation-dispersion NMR: while experiments such as those of Neudecker et al. provide atomic-resolution insight into individual aggregation-prone excited states, our approach aims to identify and compare fluctuation propensities across many sequences, thereby motivating and guiding where such detailed structural studies may be most informative.

In many cases, (A for example. 40-42 residues – an example of IDP) the ground state is disordered. Such proteins aggregate or undergo phase separation, possibly by population low population excited states. How would the current technique deal with IDPs? I should have asked this before, and I apologize.

The sizes of proteins that the authors cite for aggregation are greater than the lengths probed here. What are the prospects of extension to larger proteins? It is not necessary to do new experiments but would be nice to know what the authors think.

Although I am not entirely satisfied with the answers to the technical questions, they are good enough. However, they ought to make clear (preferably in the Discussion) that what is measured are some sort of exchange rates, which are them manipulated to obtain fluctuations and opening free energies (like Equation in line 1078). The quation in that line too ad hoc for my taste.

We have revised the Discussion to explicitly clarify the applicability of the approach to intrinsically disordered proteins (IDPs), the prospects for extending the method to larger proteins, and the scope of the measurements:

1) Applicability to intrinsically disordered proteins (IDPs)

We now explicitly state in the Discussion that proteins whose ground states are predominantly disordered typically fall outside the dynamic range of the current experiments:

“Proteins whose ground states are predominantly disordered are typically outside the dynamic range of the current experiments, as their backbone amides exchange faster than our shortest labeling times. For such systems, as intrinsically disordered proteins (IDPs), rapid HDX implementations are required to access the relevant exchange kinetics (Chow et al. 2023). Importantly, such extensions would require modifications at the instrumentation level but are fully compatible with the computational analysis and modeling framework described here.”

2) Prospects for extension to larger proteins

We have expanded the Discussion to clarify that larger proteins should be amenable to our approach and our perspective on the future directions on that topic:

“Larger domains should be amenable to our approach as well (Dumoulin et al. 2005). Extending the approach to larger proteins will primarily require evaluating how the number and spacing of exchange timepoints affect the accuracy and identifiability of inferred exchange rates as the

number of exchanging sites increases. Systematic benchmarking of time-course sampling density versus rate inference accuracy will therefore be an important direction for future work."

3) Clarification that exchange rates are measured and free energies are inferred

To address this point directly, we have revised the Discussion to clearly state the nature of what is measured and what is derived from our experiments:

"Our inferred rates depend on approximations about back exchange and the validity of combining data measured at different pH. Our inferred opening energies are approximations because they are calculated from measured exchange rate constants and approximate chemical exchange rates using an explicit kinetic model (Fig. 1E: Eq. 2), rather than being directly observed thermodynamic quantities."

We hope these revisions adequately address the reviewer's concerns and improve the clarity of the manuscript.
